# Supplementary material for: The clinical academic workforce of the future: a cross-sectional study of factors influencing career decision-making among clinical PhD students at two research-intensive UK universities
Source: BMJ Open. 2017 Aug 28;7(8):e016823. doi: 10.1136/bmjopen-2017-016823 (PMC5724086; doi:10.1136/bmjopen-2017-016823)
Supplement: Supplementary file 1 [file bmjopen-2017-016823supp001.pdf]

The clinical academic workforce of the future: a cross-sectional study of factors influencing career decision-making among clinical PhD students at two research-intensive UK universities

SUPPLEMENTARY INFORMATION

Box 1 – STROBE diagrams: a) Oxford census survey; b) UCL census survey

Box 2 – Questionnaire development and questions used

Box 3 – Analysis of open questions

Box 4 – Additional information about respondents: a) Gender and fee status of clinical doctoral students: percentages by university and for all respondents; b) Specialty of respondents: percentages by university and for all respondents

Box 5 – Respondents' predoctoral involvement in medical research and reason for the doctorate: percentages by gender and for all respondents

Box 6 – Variables not associated with wanting a clinical academic career (CAC) *vs.* having other plans or being undecided: statistically *non*-significant X^2 tests

Box 7 – Factors influencing respondents' thinking about their long-term career: percentages for those who want a clinical academic career (CAC) and those who do not/are undecided, and for all respondents

Box 8 – Variables not associated with likelihood of seeking a CL: statistically *non*-significant X^2 tests

Box 9 – Sources of information about possible career paths: by gender for those who want a clinical academic career (CAC) and those who have other plans/are undecided, and for all respondents

Box 1 – STROBE diagrams

A. Oxford census survey

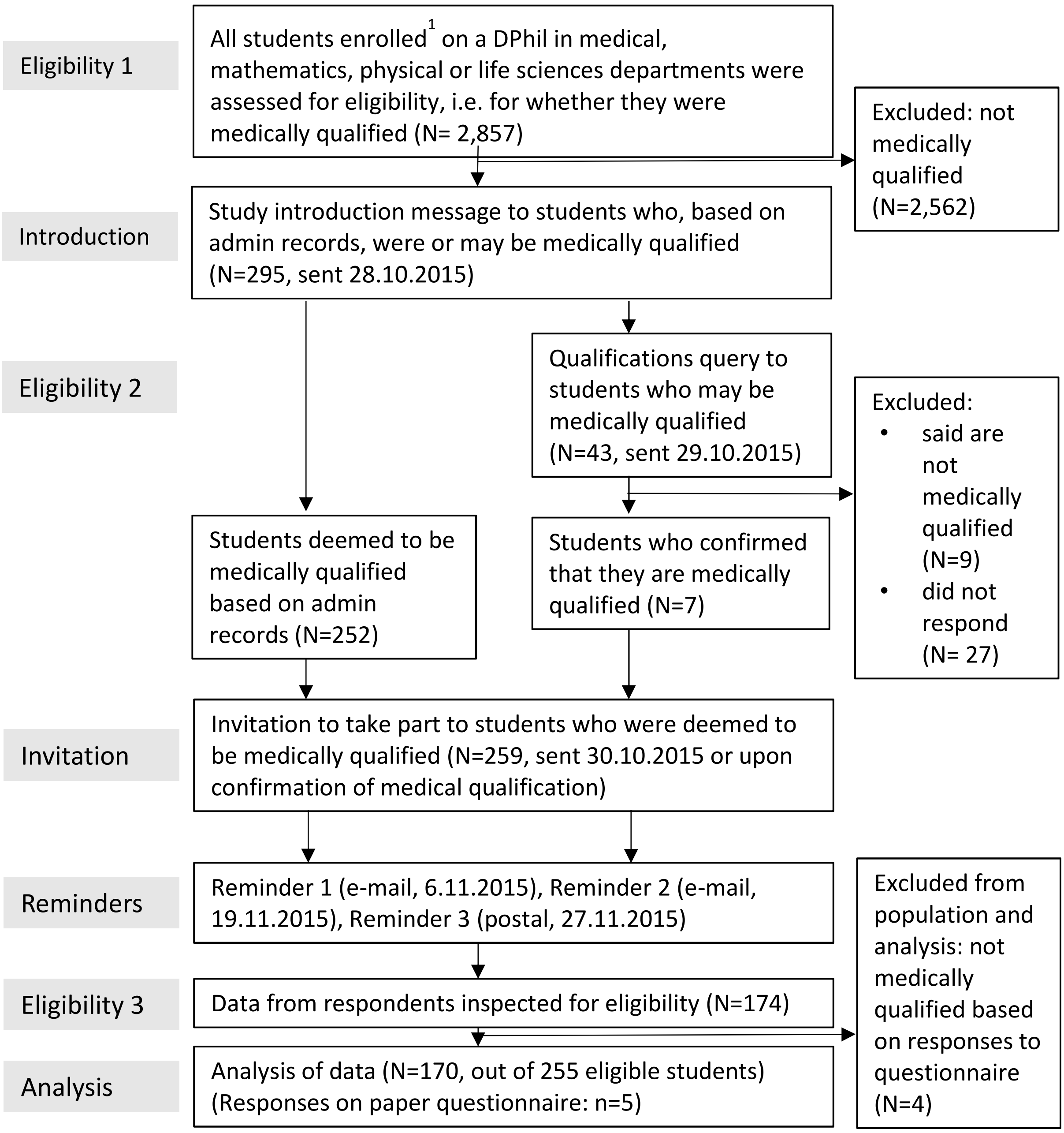

Box 1 (cont.)

B. UCL census survey

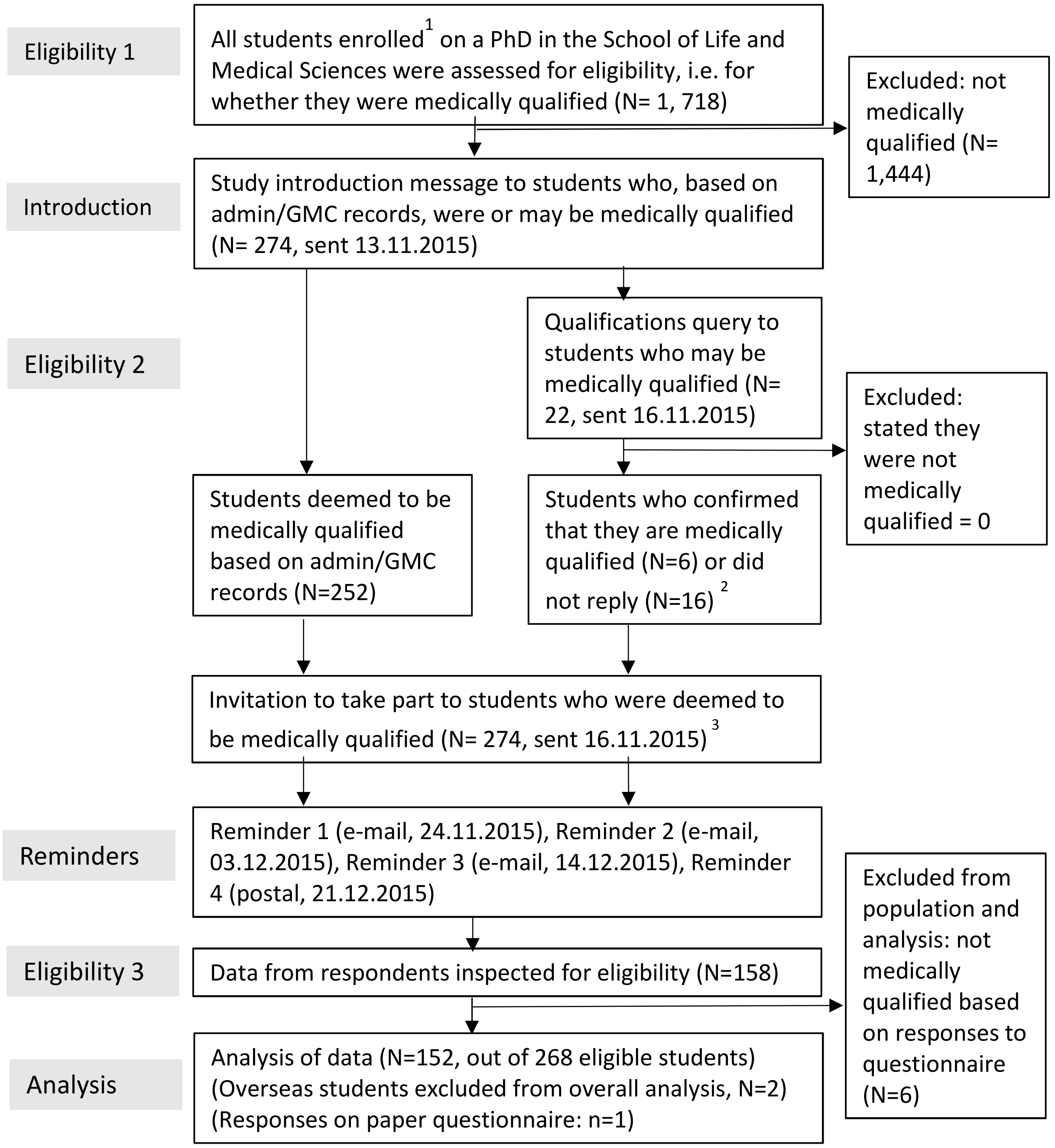

Notes

[1] All DPhil students who were enrolled on a full-time or part-time programme were assessed for eligibility (although at Oxford there were no part-time students). Suspended students were excluded as well as those who had already submitted their thesis.

[2] Both those who confirmed that they were medically qualified and those who did not reply were retained in this census survey.

[3] Due to administrative constraints, a number of students (N=33) were invited on 18.01.16 and data collection finished on 3.03.16.

Box 2 – Questionnaire development and questions used

OUCAGS developed the questionnaire, as part of its Clinical DPhil Paths longitudinal study (2013-2018) of the career plans of clinical doctoral students, as follows:

1) JL developed some of the questions below and adapted others from existing questionnaires.
2) The face validity of the questions was then checked by DB and CP, followed by question refinement by JL, DB and CP.
3) Further face validation was performed in a pilot exercise with three volunteer clinical lecturers who had completed PhDs. (Two volunteers completed the questionnaire online and one on paper.)
4) Questions were revised to address any wording issues which pilot volunteers raised.

Ahead of the 2015 data collection, and following analysis of the Oxford 2014 focus-group data, the question about knowledge of clinical lectureships was added. Also, the wording of the questions about qualifications was reviewed in discussion with UCL and small adjustments were made. For its survey, UCL also made minor adjustments to the wording of questions, such as using the term ''PhD'' instead of ''DPhil''.

0. Qualifications

0.A. What academic qualifications do you hold? (*Tick all that apply)*

1. BSc
2. BMedSci
3. BA
4. MB ChB (or equivalent)
5. MD (taught)
6. MD by research
7. MSc (taught)
8. MSc by research
9. MRes
10. MPhil
11. Other (please specify):

0.B. What fields are your qualifications in? (*Tick all that apply)*

1. Medicine
2. Dentistry
3. Biomedical field
4. Non-medical life, mathematical or physical science (e.g. zoology, chemistry, computer science)
5. Any other field(s) (e.g. humanities, social sciences)

Note: These questions were used to establish whether respondents were medically qualified.

Box 2 (cont.)

1. Medical training

1.A. Which level of training are you currently in? (*Tick one*)

1. Foundation programme (or equivalent) **[→ SKIP Questions 1.B. & 1.C.]**
2. Core training (or equivalent) **[→ SKIP Questions 1.B. & 1.C.]**
3. Acute Care Common Stem (or equivalent) **[→ SKIP Questions 1.B. & 1.C.]**
4. Specialty training (or equivalent) **[→ Go to Question 1.C.]**
5. NOT APPLICABLE: I am between levels of training **[→ Go to Question 1.B.]**
6. NOT APPLICABLE: I have left training and do not plan to return **[→ Go to Question 1.B.]**
7. NOT APPLICABLE: I have fully completed my training **[→ SKIP Questions 1.B. & 1.C.]**

1.B. What is the highest stage of clinical training which you have FULLY COMPLETED so far? (*Tick one*)

1. Medical School **[→ SKIP Question 1.C.]**
2. Foundation Programme (or equivalent) **[→ SKIP Question 1.C.]**
3. Core Training (or equivalent) **[→ SKIP Question 1.C.]**
4. Acute Care Common Stem (or equivalent) **[→ SKIP Question 1.C.]**
5. Specialty Training (or equivalent) **[→ SKIP Question 1.C.]**

1.C. Please specify your Specialty Training level. (*Enter ST or SpR, followed by a number 1 to 10*):
[text box]

1.D. What is, or will be, your medical specialty? *If you have not yet decided, please enter 'not applicable'.*
[text box]

Box 2 (cont.)

2. Prior involvement in research

2.A. What involvement in medical research did you have prior to your [DPhil/PhD]? (*Tick all that apply*)

1. No involvement
2. Intercalated degree
3. Research rotation as an Academic Foundation Programme doctor
4. Research block or regular release as an Academic Clinical Fellow (England, Wales, Northern Ireland)
5. Research block or regular release as a Clinical Research Fellow (Scotland)
6. Other post with time for research
7. Other (please specify)

3. DPhil / PhD

3.A. What was your MAIN reason for choosing to do a [DPhil/PhD]? (*Tick one*)

1. Interest in my area of research
2. Improving my prospects for an academic/research career
3. Improving my prospects for a clinical career outside academia
4. Other reason (please specify)

Note: This question is adapted from Hodsdon and Buckley (2011) – see References.

3.B. Overall, how much encouragement do you receive from your [DPhil/PhD] supervisor(s) to reflect on your career development needs? (*Tick one*)
Response options: A great deal; A lot; A moderate amount; A little; None at all

Box 2 (cont.)

4. Career paths

4.A. In your long-term career, in what type of posts do you mainly intend to work? (*Tick one*)

1. Clinical academic posts **[→ Skip Question 4.B.]**
2. Clinical service posts with some teaching and research
3. Clinical service posts with some research
4. Clinical service posts with some teaching
5. Clinical service posts without teaching or research
6. Research-only posts **[→ Skip Question 4.B.]**
7. Undecided
8. Other (please specify)

Note: This question is adapted from Goldacre *et al.* (2011) – see References.

4.B. How much would each of the following be a reason for excluding a clinical academic career from your long-term career plans? (*Tick one per row*)
Response options: A great deal; A lot; A moderate amount; A little; Not at all; Not applicable to me

1. The difficulty of obtaining research grants
2. The small number of senior academic appointments available
3. The competing pressures from service, teaching, and research
4. The limited future financial prospects compared to alternative careers
5. My perception of less favourable conditions of employment compared with NHS colleagues (in respects other than salary)
6. The possibility of a significantly longer training period to completion of training compared with NHS colleagues
7. Lack of exit paths if pursuing an academic career path and then changing my mind
8. Needing work arrangements that enable me to meet caring responsibilities (for children and/or adults)
9. Family circumstances making me unwilling to move for work
10. Realising that research is not for me
11. Other reasons (please specify)

Note: This question is adapted from Goldacre *et al.* (2011) – see References.

Box 2 (cont.)

4.C. How much does each of the following factors influence your thinking about your long-term career? (*Tick one per row*)
Response options: A great deal; A lot; A moderate amount; A little; Not at all

1. Wanting a career that fits my domestic circumstances
2. Future financial prospects
3. Wanting a career with acceptable working hours/working conditions
4. Advice from others
5. Wanting intellectual stimulation
6. Wanting to make a difference to patients
7. Lack of posts in other possible career paths
8. Wanting to do what I really want to do (enthusiasm/commitment)
9. Self-appraisal of own skills/aptitudes
10. Career promotion and prospects
11. Exposure to role models
12. Other (please give details)

Note: This question is adapted from Goldacre *et al.* (2011) – see References.

4.D. What might make a clinical academic career more attractive to you?
[text box]

4.E. How well do you feel your [DPhil/PhD] programme will/would prepare you for a clinical academic career, if this is/was your chosen career path? (*Tick one*)
Response options: Extremely well; Very well; Moderately well; Slightly well; Not at all well

4.F. How much information do you get about possible career paths from each of the following? (*Tick one per row*)
Response options: A great deal; A lot; A moderate amount; A little; None at all; Not applicable to me

1. My academic/research supervisor(s)
2. Senior academics in my department
3. My peers undertaking a [DPhil/PhD] at the [University of Oxford / UCL]
4. [OUCAGS / ACO]
5. Mentor(s)
6. Newsletters/publications from medical bodies (e.g. GMC, NIHR)
7. Other (please specify)

Box 2 (cont.)

5. Clinical Lectureships

5.A. Please tell us how accurately the following statement describes you: 'If I wanted, I could become a Clinical Lecturer in England, at an appropriate point after completing my [DPhil/PhD]'. (*Tick one*)
Response options: Extremely accurately; Very accurately; Moderately accurately; Slightly accurately; Not at all accurately

Note 1: Respondents who said that, in their long-term career, they intended to work mainly in ''clinical service posts without teaching or research''@ Question 4.A. →**SKIP Question 5.B..**

Note 2: This question is intended to operationalise self-efficacy as described in Ajzen (1991) – see References.

5.B. How likely are you to seek a Clinical Lecturer post in England, at an appropriate point after you complete your [DPhil/PhD]? (*Tick one*)
Response options: Extremely likely; Very likely; Moderately likely; Slightly likely; Not at all likely

Note: This question is intended to operationalise intention (of seeking a CL post) as described in Ajzen (1991) – see References.

5.C. How much do you feel that you know about Clinical Lecturer posts in England (including how to apply and what the posts entail)? (*Tick one*)
Response options: A great deal; A lot; A moderate amount; A little; Nothing at all

6. About respondents

6.A. What is your sex? (*Tick one*)
Response options: Male; Female

Note: To check whether respondents were representative of the whole population, we used administrative data about the whole population of clinical doctoral students invited to respond to our questionnaire. At the University of Oxford, only administrative data about clinical doctoral students' sex (collected as ''male'' or ''female'') was available for use in our study. Data on gender was not available. To ensure that data from our questionnaire would be comparable to the administrative data, we asked participants about their sex rather than their gender. However, in the presentation and discussion of results, we opted to refer to data on whether respondents ticked themselves as being male or female as ''gender'' because this, not their biological sex, is the relevant issue.

6.B. In which year were you born?
[text box: ''19_______'']

Box 2 (cont.)

6.C. Do you have a mentor, or mentors, who you meet regularly? By mentor we mean someone who is not your line manager or supervisor, and who helps you develop your knowledge, work or thinking. (*Tick one*)

1. I have a mentor/mentors, as part of a formalised mentoring relationship
2. I have an informal mentor/mentors
3. I do not have a mentor, but would like to find one
4. I do not have a mentor and do not feel the need, or have the time, for one
5. Other (please specify)

6.D. For fees purposes, what is your normal place of residence registered as? (*Tick one*)
Response options: Home (UK); Other European Union; Non-European Union

6.E. In which calendar year did you start your [DPhil/PhD] programme? (*Tick one*)
Response options: 2011 or earlier; 2012; 2013; 2014; 2015

Box 3 – Analysis of open questions

A. A number of closed questions had a final open item inviting participants to add their own response. However, none of these open items yielded recurrent themes when responses were reviewed by JL (Oxford data) and VR (UCL data).

B. The question "what might make a clinical academic career more attractive to you?" did yield recurrent themes and analysis was as follows:

1) JL produced a coding frame based on the Oxford data.
2) In a face-to-face meeting, JL and VR revised the coding frame with reference to data from both universities, and then used it to double-code anonymised responses from both Oxford and UCL. Any differences in coding between the researchers were discussed and resolved jointly.

Responses centred around two themes:

- **better post availability/a better career pathway** (mentioned by 38%, n=120 of all respondents), for example:

  "*Clearer pathways for balancing clinical training and research. As it is, it is very disruptive to either part of the career to pursue a clinical academic pathway.*" (Oxford participant)

  "*Availability of suitable posts, allowing for my (slightly unusual) combination of clinical specialty and research interests.*" (Oxford participant)

  "*Clear pathway for post-doctoral funding e.g buffer period between PhD completion and establishing independence*." (UCL participant)

  "*If there were more opportunities created by universities to fund part time research integrated with clinical consultant roles; as one nears the end of training and a senior lectureship seems very very unlikely, the choice is either struggle on and ultimately fail or drop research*." (UCL participant)

- **greater career/funding security** (mentioned by 28%, n=90 of all participants), for example:

  "*Availability of longer term funding and job security*" (Oxford participant)

  "*More financial and job security - applying for grants every 5 years or losing your job is not appealing. NHS consultants have far more job security once appointed*." (UCL participant)

Box 4 – Additional information about respondents

A. Gender and fee status of clinical doctoral students: percentages by university and for all respondents

	Oxford (N=170)		UCL (N=150)		Total (N=320)	
**Gender**	**n**	***%***	**n**	***%***	**n**	***%***
Male	108	***64***	66	***44***	174	***54***
Female	61	***36***	81	***54***	142	***44***
No response	1	***1***	3	***2***	4	***1***
**Fee status**	**n**	***%***	**n**	***%***	**n**	***%***
UK/EU	131	***77***	148	***99***	279	***87***
Overseas	33	***19***	-	**-**	33	***10***
No response	6	***4***	2	***1***	8	***3***

Note: Due to rounding, percentages may not add to 100.

B. Specialty of respondents: percentages by university and for all respondents

	Oxford (N=170)		UCL (N=150)		Total (N=320)	
**Specialties§**	**n**	***%***	**n**	***%***	**n**	***%***
Medicine	70	***41***	80	***53***	150	***47***
Surgery	30	***18***	11	***7***	41	***13***
Other	54	***32***	53	***35***	107	***33***
None	16	***9***	6	***4***	22	***7***

Note: Due to rounding, percentages may not add to 100.
§ Specialties were grouped under "medicine" and "surgery" according to the groupings used *in* GMC. *National training survey 2013: undermining*. London: General Medical Council, 2013;13-14 http://www.gmc-uk.org/NTS_2013_autumn_report_undermining.pdf_54275779.pdf (accessed 11 May 2017). In addition, "infectious diseases and microbiology" was classified as "medicine"; any sub-type of "surgery" not listed in the above document (e.g. "transplant surgery" or "paediatric surgery") was classed as "surgery".

Box 5 – Respondents' predoctoral involvement in medical research and reason for the doctorate: percentages by gender and for all respondents

	Malesǂ (N=174)		Femalesǂ (N=142)		Total (N=320)	
	**n**	**%**	**n**	**%**	**n**	**%**
**Previous involvement in medical researchǂǂ**						
High involvement level *(intercalated degree and post(s) with research*, and possibly "other" experience, too)*	34	20	28	20	62	19
Some involvement *(intercalated degree or post(s) with research*, possibly with other experience, or "other" experience)*	112	64	91	64	206	64
No involvement	22	13	15	11	38	12
No response	6	3	8	6	14	4
**Main reason for doctorate**						
Interest in area of research	85	49	60	42	146	46
Improving prospects for an academic/research career	70	40	61	43	134	42
Improving prospects for a clinical career outside academia	16	9	17	12	33	10
Other	3	2	4	3	7	2

Note: Due to rounding, column percentages may not add to 100.

ǂFour respondents did not provide their gender.

ǂǂ More than one answer could be given to the question about involvement in medical research prior to the DPhil/PhD – see Box 2, Question 2.A..

*The following response options were considered to be 'posts with research': research rotation as an Academic Foundation Programme doctor; research block or regular release as an Academic Clinical Fellow (England, Wales, Northern Ireland); research block or regular release as a Clinical Research Fellow (Scotland); other post with time for research.

Box 6 – Variables not associated with wanting a clinical academic career (CAC) *vs.* having other plans or being undecided: statistically *non*-significant X^2 tests

		Respondents wanting a CAC		X^2 test		
**Variables**	**Groupings**	**%**	**n / N**	**df**	**X^2**	**p**
**Gender**	Male	55	96 / 174	1	3.430	.064
	Female	45	63 / 141			
**Fee status**	UK/EU	50	139 / 278	1	.027	.869
	Non-EU (Overseas)	52	17 / 33			
**Age**	<30 years	49	22 / 45	2	3.370	.185
	30-35 years	55	96 / 174			
	36 years and over	40	19 / 47			
**Training stage**	Pre-specialty	63	24 / 38	2	3.072	.215
	In specialty	48	108 / 224			
	Completed	47	27 / 57			
**Encouragement from DPhil/PhD supervisor(s) to reflect on career development needs**	A great deal/A lot	53	79 / 150	2	.927	.629
	A moderate amount	47	44 / 94			
	A little/None at all	48	36 / 75			
Influences on long-term career thinking:						
**Wanting a career that fits my domestic circumstances**	A great deal/A lot	51	89 / 175	2	.733	.693
	A moderate amount	51	53 / 105			
	A little/Not at all	43	16 / 37			
**Wanting to make a difference to patients**	A great deal/ A lot	52	140 / 267	2	3.951	.139
	A moderate amount	38	15 / 40			
	A little/ Not at all	36	4 / 11			
**Wanting a career with acceptable working hours/conditions**	A great deal/A lot	46	80 / 174	2	2.494	.287
	A moderate amount	55	60 / 109			
	A little/Not at all	54	19 / 35			
**Future financial prospects**	A great deal/A lot	51	62 / 122	2	.457	.796
	A moderate amount	51	64 / 125			
	A little/Not at all	47	33 / 71			
**Advice from others**	A great deal/A lot	57	39 / 69	2	3.602	.165
	A moderate amount	51	84 / 164			
	A little/Not at all	42	35 / 84			
**Exposure to role models**	A great deal/A lot	55	85 / 156	2	2.668	.263
	A moderate amount	46	48 / 105			
	A little/Not at all	45	25 / 56			

Box 6 *(continued)*

		Respondents wanting a CAC		X^2 test		
**Variables**	**Groupings**	**%**	**n / N**	**df**	X^2	**p**
Sources of information about possible career paths (amount of information received):						
**Academic/research supervisor(s)**	A great deal/A lot	55	57 / 104	2	2.123	.346
	A moderate amount	51	55 / 109			
	A little/None at all/N/A	45	47 / 105			
**Senior academics in department**	A great deal/A lot	57	36 / 63	2	5.557	.062
	A moderate amount	56	53 / 94			
	A little/None at all/N/A	44	70 / 161			
**Peers at same university**	A great deal/A lot	55	36 / 66	2	1.388	.500
	A moderate amount	52	56 / 108			
	A little/None at all/N/A	47	67 / 144			
**Newsletters /publications**	A great deal/A lot	54	15 / 28	2	2.589	.274
	A moderate amount	59	37 / 63			
	A little/None at all/N/A	48	107 / 225			
Mentoring:						
**Formal/informal mentor, regularly met**	Yes	51	91 / 180	1	.052	.820
	No	49	67 / 136			

Box 7 – Factors influencing respondents' thinking about their long-term career: percentages for those who want a clinical academic career (CAC) and those who do not/are undecided, and for all respondents

	Respondents wanting a CACǂ				Respondents *not* wanting a CACǂ				Total (N=320)	
	Males (N=96)		Females (N=63)		Males (N=78)		Females (N=78)			
	n	*%*	*n*	*%*	*n*	*%*	*n*	*%*	n	*%*
**Enthusiasm/commitment**										
A great deal/A lot	94	***98***	62	***98***	71	***91***	70	***90***	301	***94***
(A great deal)*	(67)	***(70)***	(40)	***(64)***	(38)	***(49)***	(43)	***(55)***	(191)	***(60)***
A moderate amount	2	***2***	1	***2***	6	***8***	7	***9***	17	***5***
A little/Not at all	0	***0***	0	***0***	0	***0***	1	***1***	1	***<1***
No response	-	-	-	-	1	***1***	-	-	1	***<1***
**Wanting intellectual stimulation**										
A great deal/A lot	95	***99***	62	***98***	61	***78***	63	***81***	285	***89***
(A great deal)*	(60)	***(63)***	(40)	***(64)***	(28)	***(36)***	(31)	***(40)***	(160)	***(50)***
A moderate amount	1	***1***	1	***2***	15	***19***	14	***18***	32	***10***
A little/Not at all	0	***0***	0	***0***	1	***1***	1	***1***	2	***1***
No response	-	-	-	-	1	***1***	-	-	1	***<1***
**Wanting to make a difference to patients**										
A great deal/A lot	82	***85***	58	***92***	59	***76***	65	***83***	268	***84***
(A great deal)*	(52)	***(54)***	(29)	***(46)***	(30)	***(39)***	(36)	***(46)***	(148)	***(46)***
A moderate amount	11	***12***	4	***6***	14	***18***	10	***13***	40	***13***
A little/Not at all	3	***3***	1	***2***	4	***5***	3	***4***	11	***3***
No response	-	-	-	-	1	***1***	-	-	1	***<1***
**Self-appraisal of own skills/aptitudes**										
A great deal/A lot	63	***66***	44	***70***	37	***47***	46	***59***	192	***60***
A moderate amount	23	***24***	15	***24***	32	***41***	25	***32***	98	***31***
A little/Not at all	10	***10***	4	***6***	8	***10***	7	***9***	29	***9***
No response	-	-	-	-	1	***1***	-	-	1	***<1***
**Wanting a career that fits my domestic circumstances**										
A great deal/A lot	48	***50***	41	***65***	38	***49***	46	***59***	176	***55***
A moderate amount	36	***38***	17	***27***	25	***32***	26	***33***	105	***33***
A little/Not at all	11	***12***	5	***8***	14	***18***	6	***8***	37	***12***
No response	1	***1***	-	-	1	***1***	-	-	2	***1***
**Wanting a career with acceptable working hours/conditions**										
A great deal/A lot	42	***44***	38	***60***	38	***49***	54	***69***	174	***54***
A moderate amount	41	***43***	19	***30***	28	***36***	19	***24***	110	***34***
A little/Not at all	13	***14***	6	***10***	11	***14***	5	***6***	35	***11***
No response	-	-	-	-	1	***1***	-	-	1	***<1***

Box 7 (continued)

	Respondents wanting a CAC‡				Respondents *not* wanting a CAC‡				Total (N=320)	
	Males (N=96)		Females (N=63)		Males (N=78)		Females (N=78)			
	n	*%*	*n*	*%*	*n*	*%*	*n*	*%*	n	*%*
**Exposure to role models**										
A great deal/A lot	52	***54***	33	***52***	34	***44***	35	***45***	157	***49***
A moderate amount	29	***30***	19	***30***	27	***35***	28	***36***	105	***33***
A little/Not at all	14	***15***	11	***18***	16	***21***	15	***19***	56	***18***
No response	1	***1***	-	-	1	***1***	-	-	2	***1***
**Career promotion and prospects**										
A great deal/A lot	53	***55***	32	***51***	35	***45***	25	***32***	146	***46***
A moderate amount	28	***29***	23	***37***	26	***33***	38	***49***	119	***37***
A little/Not at all	15	***16***	8	***13***	16	***21***	15	***19***	54	***17***
No response	-	-	-	-	1	***1***	-	-	1	***<1***
**Future financial prospects**										
A great deal/A lot	41	***43***	21	***33***	33	***42***	25	***32***	122	***38***
A moderate amount	39	***41***	25	***40***	26	***33***	34	***44***	126	***39***
A little/Not at all	16	***17***	17	***27***	18	***23***	19	***24***	71	***22***
No response	-	-	-	-	1	***1***	-	-	1	***<1***
**Advice from others**										
A great deal/A lot	23	***24***	16	***25***	19	***24***	11	***14***	69	***22***
A moderate amount	51	***53***	33	***52***	34	***44***	42	***54***	164	***51***
A little/Not at all	21	***22***	14	***22***	24	***31***	25	***32***	84	***26***
No response	1	***1***	-	-	1	***1***	-	-	3	***1***
**Lack of posts in other possible career paths**										
A great deal/A lot	7	***7***	1	***2***	8	***10***	13	***17***	30	***9***
A moderate amount	9	***9***	10	***16***	15	***19***	19	***24***	53	***17***
A little/Not at all	80	***83***	52	***83***	54	***69***	46	***59***	236	***74***
(Not at all)*	(47)	***(49)***	(43)	***(68)***	(29)	***(37)***	(26)	***(33)***	(147)	***(46)***
No response	-	-	-	-	1	***1***	-	-	1	***<1***

Notes: Due to rounding, column percentages may not add to 100. A minority of respondents rated "other" factors as part of the final, open item of the question; as no recurrent themes were identified, this item is not included in the table.
‡Base: All respondents in group who provided their gender and responded to the question about their career plans (N=315).
*Some variables were considerably skewed. Where the mode for the whole group of respondents (total) was either ''a great deal'' or ''not at all'', figures for the modal response-only are provided in brackets.

Box 8 – Variables not associated with likelihood of seeking a CL: statistically *non*-significant X^2 tests

		Respondents extremely/very likely to seek a CL		X^2 test		
**Variables**	**Groupings**	**%**	**n / N**	**df**	**X^2**	**p**
**Gender**	Male	46	78 / 171	1	.788	.375
	Female	41	56 / 138			
**Encouragement from DPhil/PhD supervisor(s) to reflect on career development needs**	A great deal/A lot	48	70 / 147	2	2.832	.243
	A moderate amount	37	34 / 92			
	A little/None at all	41	30 / 74			
Sources of information about possible career paths (amount of information received):						
**Peers**	A great deal/A lot	44	29 / 66	2	2.626	.269
	A moderate amount	49	51 / 105			
	A little/None at all/N/A	38	54 / 141			
**Newsletters/publications**	A great deal/A lot	50	14 / 28	2	1.312	.519
	A moderate amount	47	29 / 62			
	A little/None at all/N/A	41	90 / 220			
Mentoring:						
**Formal/informal mentor, regularly met**	Yes	44	77 / 177	1	.013	.910
	No	43	57 / 133			

Box 9 – Sources of information about possible career paths: by gender for those who want a clinical academic career (CAC) and those who have other plans/are undecided, and for all respondents

	Respondents wanting a CACǂ				Respondents *not* wanting a CACǂ				Total (N=320)	
	Males (N=96)		Females (N=63)		Males (N=78)		Females (N=78)			
	n	%	*n*	*%*	*n*	*%*	*n*	*%*	n	*%*
**Academic/research supervisor(s)**										
Great deal/A lot	39	**41**	*18*	**29**	23	***30***	*23*	***30***	105	***33***
A moderate amount	29	**30**	*26*	**41**	32	***41***	*21*	***27***	109	***34***
A little/None/N/A	28	**29**	*19*	**30**	23	***30***	*33*	***42***	105	***33***
No response	-	-	-	-	-	-	*1*	***1***	1	***<1***
**Senior academics in my department**										
Great deal/A lot	27	**28**	9	**14**	17	***22***	9	***12***	63	***20***
A moderate amount	29	**30**	24	**38**	22	***28***	18	***23***	94	***29***
A little/None/N/A	40	**42**	30	**48**	39	***50***	50	***64***	162	***51***
No response	-	-	-	-	-	-	1	***1***	1	***<1***
**Peers undertaking a doctorate at Oxford/UCL**										
Great deal/A lot	29	**30**	7	**11**	19	***24***	11	***14***	66	***21***
A moderate amount	33	**34**	23	**37**	24	***31***	27	***35***	108	***34***
A little/None/N/A	34	**35**	33	**52**	35	***45***	39	***50***	145	***45***
No response	-	-	-	-	-	-	1	***1***	1	***<1***
**OUCAGS/ACO**										
Great deal/A lot	13	**14**	9	**14**	3	***4***	2	***3***	27	***8***
A moderate amount	21	**22**	12	**19**	11	***14***	7	***9***	52	***16***
A little/None/N/A	61	**64**	42	**67**	64	***82***	65	***83***	236	***74***
(Not applicable) *	(4)	**(4)**	(6)	**(10)**	(12)	***(15)***	(4)	***(5)***	(26)	***(8)***
No response	1	**1**	-	-	-	-	4	***5***	5	***2***
**Mentors**										
Great deal/A lot	28	**29**	22	**35**	18	***23***	10	***13***	78	***24***
A moderate amount	28	**29**	12	**19**	25	***32***	16	***21***	81	***25***
A little/None/N/A	39	**41**	29	**46**	35	***45***	49	***63***	157	***49***
(Not applicable) *	(15)	**(16)**	(15)	**(24)**	(11)	***(14)***	(17)	***(22)***	(60)	***(19)***
No response	1	**1**	-	-	-	-	3	***4***	4	***1***
**Newsletters/publications from medical bodies**										
Great deal/A lot	11	**12**	4	**6**	6	***8***	6	***8***	28	***9***
A moderate amount	23	**24**	14	**22**	11	***14***	14	***18***	64	***20***
A little/None/N/A	62	**65**	45	**71**	61	***78***	55	***71***	225	***70***
No response	-	-	-	-	-	-	3	***4***	3	***1***

Note: Due to rounding, column percentages may not add to 100.
ǂBase: All respondents in group who provided their gender and responded to the question about their career plans (N=315).
**Question attracted ''not applicable'' responses from over 3% of respondents, so ''not applicable''-only responses are provided separately in brackets.*
