# Supplementary material for: The clinical academic workforce of the future: a cross-sectional study of factors influencing career decision-making among clinical PhD students at two research-intensive UK universities
Source: BMJ Open. 2017 Aug 28;7(8):e016823. doi: 10.1136/bmjopen-2017-016823 (PMC5724086; doi:10.1136/bmjopen-2017-016823)
Supplement: Author's manuscript [file bmjopen-2017-016823.draft_revisions.pdf]

BMJ Open

BMJ Open is committed to open peer review. As part of this commitment we make the peer review history of every article we publish publicly available.

When an article is published we post the peer reviewers' comments and the authors' responses online. We also post the versions of the paper that were used during peer review. These are the versions that the peer review comments apply to.

The versions of the paper that follow are the versions that were submitted during the peer review process. They are not the versions of record or the final published versions. They should not be cited or distributed as the published version of this manuscript.

BMJ Open is an open access journal and the full, final, typeset and author-corrected version of record of the manuscript is available on our site with no access controls, subscription charges or pay-per-view fees (http://bmjopen.bmj.com).

If you have any questions on BMJ Open's open peer review process please email info.bmjopen@bmj.com

BMJ Open

The clinical academic workforce of the future: a cross-sectional study of factors influencing career decision-making amongst clinical PhD students at two research-intensive UK universities

Journal:	*BMJ Open*
Manuscript ID	bmjopen-2017-016823
Article Type:	Research
Date Submitted by the Author:	13-Mar-2017
Complete List of Authors:	Lopes, Joana; University of Oxford Medical Sciences Division, Oxford University Clinical Academic Graduate School<br>Ranieri, Veronica; University College London, Academic Careers Office, School of Life & Medical Sciences; University College London, Department of Applied Health Research<br>Lambert, Trevor; Oxford University, Public Health<br>Pugh, Chris; University of Oxford Medical Sciences Division, Oxford University Clinical Academic Graduate School<br>Barratt, Helen; University College London, Department of Epidemiology and Public Health<br>Fulop, Naomi; UCL, Applied Health Research<br>Rees, Geraint; UCL, Faculty of Life Sciences<br>Best, Denise; University of Oxford Medical Sciences Division, Oxford University Clinical Academic Graduate School
**Primary Subject Heading**:	Medical education and training
Secondary Subject Heading:	Evidence based practice
Keywords:	EDUCATION & TRAINING (see Medical Education & Training), MEDICAL EDUCATION & TRAINING, Human resource management < HEALTH SERVICES ADMINISTRATION & MANAGEMENT

SCHOLARONE™ Manuscripts

The clinical academic workforce of the future: a cross-sectional study of factors influencing career decision-making amongst clinical PhD students at two research-intensive UK universities

Joana Lopes[1], Veronica Ranieri[2 3], Trevor Lambert[4], Chris Pugh[1], Helen Barratt[3], Naomi J Fulop[3], Geraint Rees[2], Denise Best[1]

[1]Oxford University Clinical Academic Graduate School, Room 3600 Medical Sciences Divisional Office, Level 3, John Radcliffe Hospital (Main Hospital), Oxford, OX3 9DU, UK
[2]School of Life and Medical Sciences, University College London, Gower Street, London WC1E 6BT, UK
[3] Department of Applied Health Research, University College London, Gower Street, London WC1E 6BT, UK
[4]Nuffield Department of Population Health, University of Oxford, Oxford OX3 7LF, UK

Correspondence to: Joana Lopes; joana.lopes@medsci.ox.ac.uk; T: 01865 220 412 (from end March: 01865 289 465)

Word count: 3,983

For peer review only

ABSTRACT

Objectives To examine clinical doctoral students' demographic and training characteristics, career intentions, career preparedness, and what influences them as they plan their future careers.

Design and setting Online cross-sectional census surveys at two research-intensive medical schools in England in 2015-16.

Participants All medically qualified doctoral students (N=523) enrolled at the University of Oxford and University College London were invited to participate. We report on data from 320 participants (54% male and 44% female), who were representative by gender of the invited population.

Main outcome measures Career intentions.

Results Respondents were mainly in specialty training, including close to training completion (25%, n=80), and 18% (n=57) had completed training. Half (50%, n=159) intended to pursue a clinical academic career (CAC) and 62% (n=198) were at least moderately likely to seek a clinical lectureship (CL). However, 51% (n=163) had little or no knowledge about CL posts. Those wanting a CAC tended to have the most predoctoral medical research experience [X^2 (2, N=305) = 22.19, p=.0005]. Key reasons cited for not pursuing a CAC were the small number of senior academic appointments available, the difficulty of obtaining research grants, and work-life balance.

Conclusions Findings suggest that urging predoctoral clinicians to gain varied research experience whilst ensuring availability of opportunities, and introducing more flexible recruitment criteria for CL appointments, would foster CACs. As CL posts are often only open to those still in training, the many postdoctoral clinicians who have completed training, or nearly done so, do not currently gain the opportunity the post offers to develop as independent researchers. Better opportunities should be accompanied by enhanced career support for clinical doctoral students (e.g. to increase knowledge of CLs). Finally, ways to increase the number of senior clinical academic appointments should be explored since their lack seems to significantly influence career decisions.

Key words: clinical academic, academic medicine, clinical lectureship, physician scientist, career development

For peer review only - http://bmjopen.bmj.com/site/about/guidelines.xhtml

For peer review only

Strengths and limitations of this study

- This is the first UK study to describe the career aspirations of medically qualified PhD students and what influences them as they make their postdoctoral career plans.
- Other studies about the retention of postdoctoral clinical academics are predominantly North American and reliant on retrospective data.
- The study identifies factors which are associated with clinical PhD students' decision to apply for clinical lectureships (a pivotal post in clinical academic training in England).
- As it is a cross-sectional study, causality cannot be inferred from the associations we found between career plans and other variables.
- It is not possible to say whether respondents' intentions will be implemented as planned and findings may not generalise beyond research-intensive universities in England.

For peer review only

INTRODUCTION

Clinical academics (also known internationally as physician scientists) are vital to educating medical students and, as they bring a clinical perspective to research, to discovering and realising new insights into human disease.[1] Establishing a stable pipeline that develops clinical academics,[2] continues to be of global concern, for example in the US and Australia.[3,4] In the UK anxieties date back at least to the Savill report[1] in 2000 and the subsequent Modernising Medical Careers report.[5] The establishment of the National Institute of Health Research (NIHR) in 2006, and similar developments in Scotland, Wales, and Northern Ireland, supported the formation of an integrated clinical academic training (IAT) pathway[6] designed to tackle fears of dwindling clinical academic numbers by providing clinical training posts with protected research time. In England clinical academic training opportunities start with academic foundation posts, continuing to predoctoral academic clinical fellowships (ACFs), doctoral training fellowships and, finally, postdoctoral clinical lectureships (CLs).[6] Despite these efforts, the latest report from the Medical Schools Council[7] states that more than half of UK medical schools report difficulties in recruitment to some clinical academic posts. Of additional concern is the small proportion of women who pursue clinical academic careers (CACs).[8]

A pivotal point common to clinical academic training pathways irrespective of country is the PhD.[6] Interestingly, recent career tracking of doctoral clinical training fellowships awarded by major UK research funders[9] suggests that only around a third of holders progress to a junior postdoctoral CL or the more senior clinician scientist post. (CLs are open to doctors in specialty training, whereas clinician scientists have completed their training and developed research independence.) A range of barriers and enablers have been identified as contributing to this issue, including matters relating to work-life balance, funding and mentorship.[9-11] Notwithstanding, there has been no work in the UK examining the characteristics of doctors undertaking PhDs or how they view their future career options, including why those wanting a CAC may not consider applying for a postdoctoral CL.[10,12]

In 2013, the Oxford University Clinical Academic Graduate School (OUCAGS) began a longitudinal study of its clinical doctoral students to help understand these issues. The part of the study reported here was undertaken in 2015-16 in collaboration with University College London's (UCL) Academic Careers Office (ACO). Our aims were to: describe the demographic and clinical-training characteristics of both institutions' clinical doctoral students; determine their long-term career plans and factors influencing these; examine their views regarding CL posts; and explore their preparedness for making career decisions.

For peer review only - http://bmjopen.bmj.com/site/about/guidelines.xhtml

METHOD

Design and procedures

We conducted a census survey of all medically qualified doctoral students enrolled, at the beginning of autumn 2015, at two universities in England: the University of Oxford (hereafter ‘‘Oxford’’) and UCL. Each had ethical approval from its home institution and collected its data via its own online questionnaire between November 2015 and February 2016. Participants received: an e-mail from senior academics introducing the study; an e-mail invitation which offered an £8 Amazon voucher for participation; two e-mail reminders at Oxford, three at UCL; and one postal reminder inviting them to complete either the online questionnaire or a paper version. The target population was identified from administrative records. Where we were unclear about students’ qualifications, after the introductory e-mail we sent an e-mail checking qualifications. Only those who confirmed that they were medically qualified were invited to take part. A total of 523 students were asked to participate.

Instruments

OUCAGS developed the questionnaire in 2013, as part of its Clinical DPhil Paths longitudinal study (2013-2018) of the career plans of clinical doctoral students. (‘‘DPhil’’ is the term for an Oxford PhD.) Questions were either newly created or adapted from existing questionnaires.[13,14] They covered respondents’ characteristics and career plans, including: demographic variables, medical training, predoctoral involvement in research, reason for entering the doctorate, doctoral experience, intention to pursue a CAC and a CL in England (where the NIHR funds a large number of posts), and views regarding CL posts (including how attainable they think these posts are[15]). (See Box 1 in Supplement for the questionnaire questions reported below, and for minor adaptations by UCL (e.g. use of ‘‘PhD’’ instead of ‘‘DPhil’’)).

Analysis

We merged anonymised Oxford and UCL datasets and analysed the data using SPSS 22. We used frequencies to profile respondents (e.g. age, fee status) as well as to compare respondents by gender and intention towards a CAC. For fee status, we grouped UK and EU respondents together as both were eligible for ‘‘home’’ fees and have equal employment rights within the UK. This also preserved EU respondents’ anonymity in file sharing as their number was small (n= 12 and 9 respondents, from Oxford and UCL, respectively).

We analysed responses to open questions thematically, using coding frames derived from the data. All responses were double-coded, and any coding inconsistencies were discussed and resolved jointly between the two universities.

For peer review only - http://bmjopen.bmj.com/site/about/guidelines.xhtml

As a first step, the data were examined in detail. Then variables with data collected on five- or six-point scales were summarised into three categories (e.g. ‘‘a great deal/a lot’’, ‘‘a moderate amount’’, ‘‘a little/none at all/not applicable’’ – see Results Tables), with the full set of response categories referred to where relevant (e.g. for very skewed variables). Using three categories meant that data could be more clearly presented and, in most cases, items from a same question could be entered into Chi-square tests with responses categorised in the same manner. Categories were further collapsed, where necessary, to meet the requirements of Chi-square tests for expected frequencies.[16]

We conducted Chi-square tests of association to determine which of a range of background, doctoral experience, and attitude variables were associated (at the .05 level of statistical significance) with two key career outcomes:

a) intention to pursue a CAC in the long-term (respondents saying CAC *vs.* those with other plans or undecided), and
b) likelihood of seeking a CL in England (extremely or very likely to seek such a post *vs.* not at all to moderately likely).

For statistically significant Chi-square tests, we referred to adjusted standardised residuals to assist with interpreting results. The higher a residual’s value, the more a cell contributes to the Chi-square value.

For peer review only

RESULTS

Characteristics of clinical doctoral students

Response rates

Response rates were 67% and 57% for the Oxford and the UCL surveys, respectively, giving 322 respondents in total. Only two Overseas (i.e. non-UK/EU) students responded to the UCL questionnaire and, to preserve their anonymity, their data were not shared with Oxford. This paper therefore reports on data from 320 clinical doctoral students, of which 170 (53%) were enrolled at Oxford and 150 (47%) at UCL.

Demographics and training

Of the 320 respondents, 54% (n=174) were male, 44% (n=142) female, and 1% (n=4) did not specify. Their breakdown by gender makes them representative of the combined populations of clinical doctoral students of Oxford and UCL as, based on administrative data, 57% (n=299) of students invited to complete the questionnaire were male and 43% (n=224) were female.

Most respondents were aged 30-35 years (Table 1). Figures for UK/EU respondents only (N=279) are comparable to figures for all respondents as 52% (n=146) were male and only 10% (n=28) were under 30 years. However, amongst the Overseas (Oxford) students (N=33) more respondents were male (70%, n=23) and under 30 (49%, n=16) (see Box 2 in Supplement for additional information).

Most participants were in specialty training years 3 to 8 (ST3-8), i.e. had done four or more years of clinical training after medical school and entered a specialty; this included 80 respondents (25% of the total) in years 6 to 8 (ST6-8), who were close to completing training. Around one-fifth had completed their training and some were in pre-specialty training (Table 1).

The majority (84%, n=268) of participants had been involved in medical research prior to their doctorate, but over one-tenth (12%, n=38) had no involvement (see Box 3 in Supplement for additional information). This was similar at Oxford and UCL. The most common type of predoctoral medical research experience was an intercalated degree, that is, a research degree taken during the undergraduate years (48% of respondents, n=154). Also, over one-fifth (21%, n=67) of participants had held a predoctoral fellowship (i.e. an ACF in England, Wales, or Northern Ireland (n=65), or a clinical research fellowship (CRF) in Scotland (n=2)). Additionally, some respondents had: completed an academic foundation programme (19%, n=60), held other posts with time for research (12%, n=37), and/or had "other" research-related experiences, such as research towards an MSc thesis or a research assistantship (31%, n=100).

For peer review only - http://bmjopen.bmj.com/site/about/guidelines.xhtml

Reason for entering a doctoral programme

Respondents' main reason for undertaking a doctorate centred upon interest in their area of research (46%, n=146) or improving their prospects for an academic/research career (42%, n=134). However, improving their prospects for a clinical career *outside* academia was also mentioned by some (10%, n=33) (see Box 3 in Supplement for additional information). Respondents who had completed their specialist training were particularly likely to cite interest in their area of research (56%, n=32), although over one-third (35%, n=20) specifically wanted to improve their prospects for an academic/research career.

For peer review only

Table 1 – Respondents' age, training level and long-term career plans: percentages by gender and for all respondents

	All respondentsǂ		
	Males (N=174) %	Females (N=142) %	Total (N=320) %
**Age**			
<30	17	11	14
30-35	56	54	55
36+	14	16	15
No response	13	20	17
**Highest training stage completed or underway***			
Pre-Foundation, in Foundation, or post-Foundation programme	14	10	12
In ST1-2, CT or ACCS, *or* completed ST1-2, CT or ACCS	12	8	10
In ST3-8	56	57	56
ST level not specified	3	4	4
Completed specialist training	16	21	18
**Long-term career plans**			
Clinical academic posts	55	44	50
Clinical service posts with some teaching and research	23	23	23
Clinical service posts with some research	9	9	9
Clinical service posts with some teaching	2	1	2
Clinical service posts *without* teaching or research	1	1	1
Research-only posts	2	3	3
Undecided	8	15	11
Other	1	4	2
No response	-	1	<1

Note: Due to rounding, column percentages may not add to 100.
ǂFour respondents did not provide their gender.
*In the UK, following medical school and a two-year Foundation programme, doctors enter specialist training (ST). The first two years of specialty training are usually referred to as ST1-2, but some specialties have, before specialist training, a core training (CT) or acute care common stem (ACCS) programme. However, this is highly dependent on specialty.

Long-term career planning

Career plans

Half of all respondents expressed a desire to pursue a CAC in the long term, and around a third intended to work in clinical posts with some research, or some teaching and research (Table 1). The figure for respondents wanting a CAC was higher amongst former ACFs/CRFs (63%, n=42). Over one-tenth of respondents, including 13% (n=9) of former ACFs/CRFs and 12% (n=27) of doctors in specialty training, were undecided about their long-term career, although very few wanted purely

For peer review only - http://bmjopen.bmj.com/site/about/guidelines.xhtml

clinical posts or clinical posts with teaching. The proportion of participants undecided or with each type of career plan was similar across doctoral year groups (Year 1 to Year 4 and above).

Chi-square tests showed that greater predoctoral involvement in medical research, and pursuing a doctorate mainly to improve academic/research-career prospects, were each associated with wanting a CAC (Table 2). In contrast, there was no statistically significant association between whether a respondent was planning a CAC and: gender, fee status (UK/EU *vs.* Overseas), age, training stage, or specialty (Box 4 in Supplement).

For peer review only

For peer review only - http://bmjopen.bmj.com/site/about/guidelines.xhtml

Table 2 – Variables associated with wanting a clinical academic career (CAC) *vs.* having other plans or being undecided: statistically significant X^2 tests

		Respondents wanting a CAC			X^2 test		
**Variables**	**Groupings**	**%**	**n / N**	**Adjusted residuals‡**	**df**	**X^2**	**p**
**Predoctoral involvement in medical research**	**No involvement**	26	10 / 38	-3.0	2	22.193	.0005
	**Some involvement** *(intercalated degree or post(s) with research, possibly with other experience, or "other" experience)*§	46	95 / 205	-1.4			
	**High involvement level** *(intercalated degree and post(s) with research, and possibly "other" experience, too)*§	73	45 / 62	4.1			
**Main reason for doctorate**	Interest in my area of research	49	71 / 146	-.4	2	25.515	.0005
	Improving prospects for an academic/research career	61	82 / 134	3.5			
	Other	15	6 / 39	-4.6			
Influences on long-term career thinking:							
**Wanting intellectual stimulation***	A great deal/A lot	55	157 / 284	5.4	1	29.640	.0005
	A moderate amount/A little/Not at all	6	2 / 34	-5.4			
**Enthusiasm /commitment***	A great deal/ A lot	52	156 / 300	2.9	1	8.480	.004
	A moderate amount/ A little/ Not at all	17	3 / 18	-2.9			
**Self-appraisal of own skills/aptitudes**	A great deal/A lot	56	107 / 191	2.6	2	7.743	.021
	A moderate amount	39	38 / 98	-2.7			
	A little/Not at all	48	14 / 29	-.2			
**Career promotion and prospects**	A great deal/A lot	58	85 / 146	2.7	2	7.300	.026
	A moderate amount	43	51 / 118	-1.9			
	A little/Note at all	43	23 / 54	-1.2			
**Lack of posts in other possible career paths**	A great deal/A lot	27	8 / 30	-2.7	2	14.357	.001
	A moderate amount	36	19 / 53	-2.3			
	A little/Not at all	56	132 / 235	3.7			
CL posts:							
**CL knowledge**	A great deal/A lot	73	27 / 37	3.0	2	19.198	.0005
	A moderate amount	59	69 / 118	2.3			
	A little/Nothing at all	39	63 / 162	-4.1			
**CL attainability (accuracy of statement that CL is attainable)**	Extremely/very accurate	63	60 / 96	2.9	2	16.079	.0005
	Moderately accurate	54	57 / 105	1.1			
	Slightly/Not at all accurate	36	42 / 117	-3.8			
Sources of information about possible career paths (amount of information received):							
**OUCAGS/ACO**	A great deal/A lot	82	22 / 27	3.4	2	18.917	.0005
	A moderate amount	65	33 / 51	2.2			
	A little/None at all/N/A	44	103 / 236	-4.1			
**Mentor(s)**	A great deal/A lot	64	50 / 78	2.8	2	8.778	.012
	A moderate amount	49	40 / 81	-.2			
	A little/None at all/N/A	44	68 / 156	-2.3			

‡ For explanation and interpretation see Method.
§The following were considered "posts with research": Academic Foundation Programme posts; Academic Clinical Fellow (England, Wales, Northern Ireland); Clinical Research Fellow (Scotland); and responses of "other post with time for research".
*Due to the small numbers selecting "A moderate amount", "A little" and "Not at all", these categories were merged in order to meet X^2 test assumptions (see Method).

For peer review only - http://bmjopen.bmj.com/site/about/guidelines.xhtml

Enthusiasm and commitment to a given career path, as well as wanting intellectual stimulation and to make a difference to patients, were major influencing factors in respondents' long-term career planning (Table 3, and Box 6 in Supplement for further details). In addition, having acceptable working hours and conditions was especially important to respondents who were undecided about their long-term career, with 78% (n=28) of this group saying this had a great deal or a lot of importance.

In Chi-square tests the following were positively associated with wanting a CAC (Table 2): wanting intellectual stimulation, enthusiasm/commitment, self-appraisal of own skills/aptitudes, and career promotion and prospects. Also, a perceived lack of posts in other possible career paths was negatively associated with planning a CAC, possibly as respondents wanting a CAC can always return to a full-time clinical career. There was no statistically significant association between planning a CAC and other long-term career planning factors examined (Box 4 in Supplement).

Table 3 – Factors influencing respondents' thinking about their long-term career: percentage saying ''a great deal'' or ''a lot'', by whether respondents want a clinical academic career (CAC) *vs.* having other plans or being undecided, and for all respondents

	**Respondents wanting a CACǂ** (N=159) **%**	**Respondents *not* wanting a CACǂ** (N=160) **%**	**Total** (N=320) **%**
Enthusiasm/commitment<br>*(A great deal)**	98<br>*(67)*	90<br>*(52)*	94<br>*(60)*
Wanting intellectual stimulation<br>*(A great deal)**	99<br>*(63)*	79<br>*(38)*	89<br>*(50)*
Wanting to make a difference to patients<br>*(A great deal)**	88<br>*(51)*	79<br>*(41)*	84<br>*(46)*
Self-appraisal of own skills/aptitudes	67	53	60
Wanting a career that fits my domestic circumstances	56	54	55
Wanting a career with acceptable working hours/conditions	50	59	54
Exposure to role models	54	44	49
Career promotion and prospects	54	38	46
Future financial prospects	39	38	38
Advice from others	25	19	22
Lack of posts in other possible career paths	5	14	9

ǂBase: All participants (N=319) who responded to the question about their career plans.
*Some variables were considerably skewed. Where the mode for the whole group of respondents (total) was ''a great deal'', figures for the modal response-only are provided in brackets.

For peer review only - http://bmjopen.bmj.com/site/about/guidelines.xhtml

Drawbacks of a clinical academic career

Respondents not intending to work in either clinical academia or research-only posts (N=153) were asked how much each of a range of factors might be a reason for excluding a CAC from their long-term career plans. This group included over a third (37%, n=25) of former ACFs/CRFs. Nearly two-thirds of the group (66%, n=101) said that the difficulty of obtaining research grants had a great deal or a lot of weight in their planning. Other factors with considerable weight were (% saying ‘‘a great deal’’ or ‘‘a lot’’):

- the small number of senior academic appointments available (50%, n=76)
- the competing pressures from service, teaching and research (47%, n=72)
- the limited future financial prospects compared to alternative careers (34%, n=52)
- needing work arrangements that are compatible with their caring responsibilities (30%, n=46, including 45%, n=34, of women but only 15%, n=11, of men), and
- family circumstances making them unwilling to move for work (27%, n=42).

Responses to the open question “what might make a clinical academic career more attractive to you?”, asked of all participants, confirmed that issues around obtaining research grants, securing posts, and career progression are of particular concern when considering a CAC, as responses centred around these themes.

Clinical lectureships

Over three-fifths of participants (Table 4), including 65% (n=181) of UK/EU respondents, were at least moderately likely to seek a CL post in England at an appropriate point after completing their doctoral studies. This figure rose to 78% (n=52) for former ACFs/CRFs, and to 98% (n=41) amongst former ACFs/CRFs wanting a CAC. In addition, nearly two-thirds of all participants, and over four-fifths of former ACFs/CRFs (82%, n=55), perceived CL posts to be at least moderately attainable for them (Table 4). Although overall women were more cautious about the attainability of CL posts, amongst respondents wanting a CAC similar proportions of men (74%, n=71) and women (73%, n=46) felt they were at least moderately attainable.

Respondents in speciality training, and those aged 30-35, were the most likely to seek a CL post in England. This is supported by Chi-square tests (Table 5), which also showed that there were positive associations between likelihood of seeking a CL in England and: wanting a CAC; being from the UK/EU; predoctoral involvement in medical research; knowledge of CLs; and feeling that a CL is attainable. However, there were no statistically significant associations between likelihood of

For peer review only - http://bmjopen.bmj.com/site/about/guidelines.xhtml

seeking a CL post and gender or specialty (Box 7 in Supplement.) Chi-square tests also showed a positive association between wanting a CAC and perceiving that CL posts are attainable (Table 2).

Table 4 – Respondents' likelihood of seeking a CL, and views on CL attainability and own knowledge about CLs: percentages by gender and for all respondents

	All respondentsǂ		
	Males %	Females %	Total %
**Likelihood of seeking a CL***	*N=173**	*N=141**	*N=318**
Extremely/very likely	45	40	42
Moderately likely	17	23	20
Slightly/Not at all likely	36	36	36
No response	1	2	2
**CL attainability** *Accuracy of statement that respondent could become a CL in England, if wanted*	*N=174*	*N=142*	*N=320*
Extremely/Very accurate	37	22	30
Moderately accurate	29	36	33
Slightly/Not at all accurate	33	42	37
No response	1	-	<1
**Knowledge of CL** *Amount known*	*N=174*	*N=142*	*N=320*
A great deal/A lot	12	11	12
A moderate amount	40	34	37
A little/Nothing at all	48	54	51
No response	-	1	1

Note: Due to rounding, column percentages may not add to 100.
ǂFour respondents did not provide their gender.
*The question about likelihood of seeking a CL was not asked of two participants who stated that they intended to work in clinical posts without teaching or research.

Although many respondents thought that they may successfully apply for a CL, only a minority (Table 4) felt that they knew a great deal or a lot about CL posts in England. In fact, just over half, including one-quarter (25%, n=17) of former ACFs/CRFs, felt that they had little or no knowledge (Table 4). Also, although Chi-square tests showed a positive association between wanting a CAC and knowledge of CL posts (Table 2), two-fifths (40%, n=63) of those planning a CAC nevertheless had little or knowledge of these CL posts.

For peer review only - http://bmjopen.bmj.com/site/about/guidelines.xhtml

Table 5 – Variables associated with likelihood of seeking a CL: statistically significant X^2 tests

		Respondents extremely/very likely to seek a CL			X^2 test		
**Variables**	**Groupings**	**%**	**n / N**	**Adjusted residuals‡**	**df**	**X^2**	**p**
**Fee status**	UK/EU	47	128 / 273	3.4	1	11.383	.001
	Non-EU (Overseas)	16	5 / 32	-3.4			
**Age**	<30 years	27	12 / 44	-2.5	2	11.534	.003
	30-35 years	51	89 / 173	3.4			
	36 years and over	32	14 / 44	-1.8			
**Training stage**	Pre-specialty	22	8 / 36	-2.7	2	12.149	.002
	In specialty	49	108 / 221	3.4			
	Completed	32	18 / 56	-1.8			
**Predoctoral involvement in medical research**	**No involvement**	21	8 / 38	-2.9	2	24.502	.0005
	**Some involvement** *(intercalated degree or post(s) with research, possibly with other experience, or "other" experience)* §	39	77 / 199	-1.9			
	**High involvement level** *(intercalated degree and post(s) with research, and possibly "other" experience, too)* §	68	42 / 62	4.5			
**Long-term career plans**	CAC	67	105 / 158	8.5	3	72.557	.0005
	Clinical service with research (possibly also teaching)	18	18 / 99	-6.0			
	Undecided	23	8 / 35	-2.5			
	Other plans	15	3 / 20	-2.6			
**CL knowledge**	A great deal/ A lot	65	24 / 37	2.9	2	31.140	.0005
	A moderate amount	57	66 / 116	3.8			
	A little/Nothing at all	28	44 / 158	-5.5			
**CL attainability (accuracy of statement that CL is attainable)**	Extremely/very accurate	70	66 / 95	6.3	2	52.138	.0005
	Moderately accurate	44	46 / 105	.2			
	Slightly/Not at all accurate	20	22 / 112	-6.2			
Sources of information about possible career paths (amount of information received):							
**Academic/ research supervisor(s)**	A great deal/A lot	48	49 / 103	1.2	2	6.443	.040
	A moderate amount	48	52 / 108	1.3			
	A little/None at all/N/A	33	33 / 101	-2.5			
**Senior academics**	A great deal/A lot	50	31 / 62	1.3	2	9.356	.009
	A moderate amount	53	48 / 91	2.2			
	A little/None at all/N/A	35	55 / 159	-3.0			
**OUCAGS/ACO**	A great deal/A lot	59	16 / 27	1.8	2	17.804	.0005
	A moderate amount	65	33 / 51	3.5			
	A little/None at all/N/A	36	82 / 230	-4.2			
**Mentor(s)**	A great deal/A lot	57	43 / 76	2.7	2	10.053	.007
	A moderate amount	46	37 / 81	.6			
	A little/None at all/N/A	35	53 / 152	-2.9			

‡ For explanation and interpretation see Method.
§The following were considered "posts with research": Academic Foundation Programme posts; Academic Clinical Fellow (England, Wales, Northern Ireland); Clinical Research Fellow (Scotland); and responses of "other post with time for research".

For peer review only - http://bmjopen.bmj.com/site/about/guidelines.xhtml

Career preparedness

Most respondents (64%, n=204) felt that their doctoral programme was preparing them extremely or very well for a possible future CAC, although this figure was considerably higher for men (72%, n=125) than for women (55%, n=78).

A proportion (23%, n=75) of respondents had little or no encouragement from their PhD supervisor(s) to reflect on their career development needs, but most (56%, n=180) had a formal or informal mentor they met regularly. However, only 50% (n=71) of women had such a mentor, compared to 62% (n=107) of men. Also, over a third (34%, n=109) of all respondents stated that they did not have a mentor but would like to find one, and this figure increased to 41% (n=58) amongst women (*vs.* 29%, n=50, amongst men).

Many respondents received at least moderate amounts of information about possible careers from their academic/research supervisor(s) (67%, n=214), peers (54%, n=174), mentors (50%, n=159), and senior academics in their department (49%, n=157). Respondents who wanted a CAC were more likely to say that they get a great deal or a lot of information from mentors (31%, n=50 *vs.* 18%, n=28 for those who had other plans or were undecided). However, over three-fifths of women *not* intending to pursue a CAC received little or no information from senior academics (64%, n=50 *vs.* 50%, n=39, of men) and mentors (63%, n=49 *vs.* 45%, n=35, of men). (Box 8 in Supplement contains additional data.)

Men and women obtained similar amounts of information from OUCAGS/UCL's ACO, with 28% (n=48) of men and 22% (n=31) of women receiving at least moderate amounts of information from this source. Moreover receiving information about careers from OUCAGS/ACO and mentor(s) each had a positive relationship both with wanting a CAC and likelihood of seeking a CL (Tables 2 and 5). The latter also was positively associated with receiving information about careers from academic/research supervisor(s) and senior academics. Interestingly, there were no statistically significant relationships between either wanting a CAC or likelihood of seeking a CL and: having a mentor, encouragement from supervisors to reflect on career needs, or information from peers and newsletters (Boxes 4 and 7 in Supplement).

For peer review only - http://bmjopen.bmj.com/site/about/guidelines.xhtml

DISCUSSION

The clinical doctoral students in our study are aged mainly between 30-35 years, in specialty training (with a quarter close to completion), and gender balanced once Overseas students are accounted for. Encouragingly, half of our clinical doctoral students want to pursue a CAC, with women as likely as men to express this career goal. Amongst those who have already completed their clinical specialist training (nearly one-fifth of our clinical doctoral students) there is also interest in CACs. Indeed, training level does not seem to affect long-term plans, and neither do age or clinical specialty. Greater predoctoral involvement in medical research is strongly associated with wanting a CAC but, unexpectedly, less than two-thirds of former ACFs/CRFs, i.e. doctoral students already on the clinical academic path, are planning to pursue a CAC. Given that our cohort, who secured doctorates at Oxford/UCL, might be regarded as ‘‘high-achievers’’, it is surprising that a higher proportion do not want a CAC. On a more positive note, many doctoral students, even if not planning a CAC, *are* interested in a career that combines clinical work and research, with very few wanting purely clinical posts. In common with others,[9-11] we found that uncertainties around funding and career progression influence career plans, and are perceived as drawbacks of CACs. Respondents not planning a CAC, including former ACFs/CRFs, are particularly worried about the small number of senior academic appointments available, as well as the difficulty of obtaining research grants and work-life balance. Whilst the desire for a career that fits domestic circumstances is not statistically associated with career plans, these issues are given more weight by women than men. Such factors may go on to influence women's actual postdoctoral career choices,[17] as women may evaluate barriers encountered differently to men, leading to a change in their career intentions.[18]

We were curious about the place of CL posts in career plans. These posts might be considered a logical step in a postdoctoral CAC but a recent report[9] suggests their take-up is lower than expected. In light of this, our data are encouraging as around three-fifths of respondents were at least moderately likely to apply for a CL post – a greater proportion than were planning a CAC. This might indicate that some view CL posts as valuable for career progression, whether as part of a CAC or not. Concomitantly, however, progression to a CL may be affected by the low levels of knowledge about the posts. Additionally, we found some issues with career preparedness (also reported by others[9]). These were more prominent amongst women, who were more likely not to have a mentor but want one and, if not wanting a CAC, received less guidance from senior academics. Exposure to role models, often suggested as important[19,20] in career development, did not appear to be significant amongst our respondents, but the positive association between receiving

For peer review only - http://bmjopen.bmj.com/site/about/guidelines.xhtml

information from OUCAGS/ACO and wanting a CAC is reassuring as it supports the benefit of such infrastructure.

Strengths and limitations of the study

Retention of postdoctoral clinical academics is crucial to the progress of academic medicine. However, the relevant literature is predominantly North American[8] and also tends to rely on retrospective data.[9] Ours is the first UK study to describe, based on data from two universities, the career aspirations of medically qualified PhD students and what influences them as they make their career plans. As it is a cross-sectional study, causality cannot be inferred from the associations we found between career plans and other variables. It is also not possible to say whether respondents' intentions will be implemented as planned and our findings may not generalise to other institutions as our data are from two research-intensive universities in England.

Implications of findings

Our novel study, which adds the perspective of UK clinical doctoral students to the existing literature, suggests four areas of action that will support the development of a stable pipeline of clinical academics. Firstly, we suggest promoting increased numbers of flexible opportunities for doctors to gain medical research experience, starting from their undergraduate studies. Indeed, we found that those with the most research experience were more likely to want a CAC and, although research experience may not directly influence that choice, it may contribute to an informed career decision. Secondly, institutions should offer comprehensive career support for their clinical doctoral students. This could be via delivery of a targeted careers package by a local institutional unit similar to OUCAGS/ACO which could: address knowledge about CL posts and other postdoctoral opportunities; facilitate mentors and doctoral students to develop individualised career plans; meet the unfulfilled need for mentors; and brief senior academics on current CAC opportunities to enable them to provide up-to-date guidance. Thirdly, we suggest a need to increase the flexibility of CAC paths even further than at present. CL posts are the first postdoctoral position to afford clinicians the opportunity to develop as independent research leaders, and without this opportunity progressing further academically may be difficult. We have identified that family mobility is an issue for our clinical doctoral students, but identifying a CL post in the right specialty, in the right place and at the right time (e.g. as the doctorate comes to an end) is challenging, and sometimes impossible. This is exacerbated by eligibility criteria, particularly for NIHR CL posts, requiring that applicants have at least one year of training remaining (the post ceases at completion of training), since, as we found, many clinical doctoral students have completed, or nearly completed, their training. We therefore suggest that criteria for CL posts be relaxed. Various postdoctoral points of entry into CL posts could be allowed (including after training completion) and the posts should not

For peer review only - http://bmjopen.bmj.com/site/about/guidelines.xhtml

be specialty-specific so as to afford enhanced opportunities to all postdoctoral clinicians. Increased flexibility might also attract back postdoctoral clinicians who had originally not pursued a CAC, such as when family circumstances change and work-life balance may seem more achievable. Such changes could help medical schools overcome the recruitment challenges that have been reported.[7] Finally, more senior posts are required since their lack seems to significantly influence career decisions. Universities are unlikely to be able to create sufficient fully funded senior posts, so formalising research time in a subset of NHS consultant posts, and establishing them as joint university appointments, could cost-effectively expand the definition of a CAC. Ensuring the visibility of these posts would demonstrate a clear career trajectory to encourage postdoctoral retention. Although current NHS service and funding pressures might make this seem low priority, we would argue that such joint appointments are essential as research activity is centrally positioned in the NHS constitution[21] and, crucially, it improves patient care and service quality.[22,23]

Conclusions and further research

Our findings suggest that many doctoral students with the potential to be the clinical academics of the future may never tread this path, some (former ACFs/CRFs) may choose to abandon it, and some may be excluded by inflexible eligibility criteria for postdoctoral posts. It is therefore important for those promoting CACs to provide sufficient, flexible opportunities to engage in clinical academia to all postdoctoral clinicians. This should include tackling issues around the career pathway beyond the clinical training period and building career preparedness and resilience. Our findings also suggest that an additional approach to increasing the numbers of clinical academics might be to ensure predoctoral doctors have ample opportunities to gain research experience. Further research should investigate whether clinical doctoral students' intentions will be implemented as planned (e.g. will equal numbers of men and women actually develop CACs?) and seek to identify which factors contribute to actually pursing a CAC. The Clinical DPhil Paths longitudinal study is currently collecting data on this in the UK.

For peer review only - http://bmjopen.bmj.com/site/about/guidelines.xhtml

FOOTNOTES

Acknowledgements: We thank the clinical doctoral students who took part in our study and Abigail Hipkin (Research Education and Training Administrator, OUCAGS), who assisted with data collection at Oxford. We are also grateful to the University of Oxford and UCL administrative services and the UCL graduate tutors who helped us identify the students to invite to participate in our study.

Contributors: JL, DB and CP developed the Clinical DPhil Paths questionnaire. JL collected the Oxford data and VR collected the UCL data. All authors conceived the data analysis plan and made substantive contributions to the interpretation of findings. JL conducted the quantitative data analysis and reporting, with assistance from VR. JL and VR undertook the qualitative data analysis. JL and DB wrote the first draft. All authors contributed to further drafts and all approved the final version. All authors had full access to all of the data (including statistical reports and tables) in the study and can take responsibility for the integrity of the data and the accuracy of the data analysis. The study guarantors are DB (for Oxford) and GR (for UCL).

Funding: Oxford – This research was funded by OUCAGS, which is part of the University of Oxford Medical School. UCL – This research was funded by the National Institute for Health Research University College London Hospitals Biomedical Research Centre. NJF and HB were (in part) supported by the National Institute for Health Research (NIHR) Collaboration for Leadership in Applied Health Research and Care (CLAHRC) North Thames at Bart's Health NHS Trust. The views expressed are those of the author(s) and not necessarily those of the NHS, the NIHR or the Department of Health. The authors conducted the study with no involvement from the funders.

Competing interests: All authors have completed the ICMJE uniform disclosure form at http://www.icmje.org/coi_disclosure.pdf and declare: JL, CP and DB declare that JL and DB are employed by, and CP directs, OUCAGS, which exists to promote and advance clinical academic careers; CP declares that his salary is in part paid for by Health Education England in the Thames Valley; VR and GR declare that the former works for, and the latter manages, UCL's Academic Careers Office, which is funded by UCLH's Biomedical Research Centre, and thus the National Institute for Health Research; no other financial relationships with any organisations that might have an interest in the submitted work in the previous three years besides the support provided for the submitted work by the organisations described above under ''funding''; no other relationships or activities that could appear to have influenced the submitted work.

Ethical approval: Oxford survey approved by the University of Oxford's Medical Sciences Inter-Divisional Research Ethics Committee (reference MSD-IDREC-C1-2014-149). UCL survey approved by

For peer review only - http://bmjopen.bmj.com/site/about/guidelines.xhtml

the UCL Research Ethics Committee (application 7335/002). All participants gave informed consent to take part.

Data sharing: No additional data available.

Transparency: The study guarantors affirm that this manuscript is an honest, accurate, and transparent account of the study being reported; that no important aspects of the study have been omitted; and that any discrepancies from the study as planned have been explained.

Licence: The Corresponding Author has the right to grant on behalf of all authors and does grant on behalf of all authors, a worldwide licence (http://www.bmj.com/sites/default/files/BMJ%20Author%20Licence%20March%202013.doc) to the Publishers and its licensees in perpetuity, in all forms, formats and media (whether known now or created in the future), to i) publish, reproduce, distribute, display and store the Contribution, ii) translate the Contribution into other languages, create adaptations, reprints, include within collections and create summaries, extracts and/or, abstracts of the Contribution and convert or allow conversion into any format including without limitation audio, iii) create any other derivative work(s) based in whole or part on the on the Contribution, iv) to exploit all subsidiary rights to exploit all subsidiary rights that currently exist or as may exist in the future in the Contribution, v) the inclusion of electronic links from the Contribution to third party material where-ever it may be located; and, vi) licence any third party to do any or all of the above. All research articles will be made available on an Open Access basis (with authors being asked to pay an open access fee—see http://www.bmj.com/about-bmj/resources-authors/forms-policies-and-checklists/copyright-open-access-and-permission-reuse). The terms of such Open Access shall be governed by a Creative Commons licence—details as to which Creative Commons licence will apply to the research article are set out in our worldwide licence referred to above.

For peer review only

For peer review only - http://bmjopen.bmj.com/site/about/guidelines.xhtml

REFERENCES

1 Academy of Medical Sciences. *The tenure-track clinical scientist: a new career pathway to promote recruitment into clinical academic medicine*. London: AMS, 2000.

2 Academy of Medical Sciences. *Clinical academic medicine in jeopardy: recommendations for change.* London: AMS, 2002 https://www.acmedsci.ac.uk/viewFile/publicationDownloads/pclinaca.pdf (accessed 7 Dec 2016).

3 Milewicz DM, Lorenz RG, Dermody TS, et al. Rescuing the physician-scientist workforce: the time for action is now. *J Clin Invest* 2015;125(10):3742-47. doi:10.1172/JCI84170

4 Windsor J, Searle J, Hanney R, et al. Building a sustainable clinical academic workforce to meet the future healthcare needs of Australia and New Zealand: report from the first summit meeting. *Intern Med J* 2015;45(9):e965:e971. doi:10.1111/imj.12854

5 Modernising Medical Careers. *Medically- and dentally-qualified academic staff: recommendations for training the researchers and educators of the future. Report of the Academic Careers Sub-Committee of Modernising Medical Careers and the UK Clinical Research Collaboration*. London: UK CRC, 2005 http://www.ukcrc.org/wp-content/uploads/2014/03/Medically_and_Dentally-qualified_Academic_Staff_Report.pdf (accessed 7 Dec 2016).

6 Funston G, Cera C, Kirkham D, et al. The road to a clinical academic career. *BMJ Careers 2015*. http://careers.bmj.com/careers/advice/The_road_to_a_clinical_academic_career

7 Medical Schools Council. *A survey of staffing levels of medical clinical academics in UK medical schools, as at 31 July 2015*. London: Medical Schools Council, 2016. http://www.medschools.ac.uk/SiteCollectionDocuments/MSC-survey-2016.pdf (accessed 9 Mar 2017).

8 Edmunds LD, Ovseiko PV, Shepperd S, et al. Why do women choose or reject careers in academic medicine? A narrative review of empirical evidence. *Lancet* 2016;Apr19. doi:10.1016/S0140-6736(15)01091-0 (accessed 28 Apr 2016).

9 Medical Research Council, Academy of Medical Sciences, British Heart Foundation, et al. *A cross-funder review of early-career clinical academics: enablers and barriers to progression*. London: MRC, 2015 https://www.mrc.ac.uk/documents/pdf/review-of-early-career-clinical-academics/ (accessed 26 Jan 2017).

10 Ranieri V, Barratt H, Fulop N, et al. Factors that influence career progression among postdoctoral clinical academics: a scoping review of the literature. *BMJ Open* 2016;6:e013523. doi:10.1136/bmjopen-2016-013523

For peer review only - http://bmjopen.bmj.com/site/about/guidelines.xhtml

11 Ranieri V, Barratt H, Fulop N, et al. Clinical academics' postdoctoral career development. *BMJ* 2015;351:h6945. doi:10.1136/bmj.h6945 (Published 30 December 2015).

12 Best D, Lopes J, Pugh C. It's the duty of every doctor to get involved with research. *BMJ* Online [eLetter] 27 November 2015. doi:10.1136/bmj.h6329 http://www.bmj.com/content/351/bmj.h6329/rapid-responses (accessed 9 Mar 2017).

13 Hodsdon L, Buckley A. Postgraduate Research Experience Survey: 2011 results. Higher Education Academy 2011 www.heacademy.ac.uk/resources/detail/postgraduate/PRES_2011_report (accessed 6 Jul 2016).

14 Goldacre MJ, Lambert T, Goldacre R et al. Career plans and views of trainees in the academic clinical fellowship programme in England. *Med Teach* 2011;33:11:e637-e643. doi: 10.3109/0142159X.2011.610842

15 Ajzen I. The theory of planned behaviour. *Organ Behav Hum Decis Process* 1991;50(2):e179-211.

16 Bland M. An introduction to medical statistics. Oxford: Oxford University Press 2015:193–197.

17 Levine RB, Lin F, Kern DE, et al. Stories from early-career women physicians who have left academic medicine: a qualitative study at a single institution. *Acad Med* 2011;86:6:e752-e758.

18 Ng THW, Sorensen KL, Eby LT, et al. Determinants of job mobility: a theoretical integration and extension. *J Occup Organ Psychol* 2007;80: e363-e386. doi:10.1348/096317906X130582

19 Levinson W, Kaufman K, Clark B et al. Mentors and Role Models for Women in Academic Medicine. *West J Med* 1991;154:423-426.

20 Borges NJ, Navarro AM, Grover A, et al. How, when, and why do physicians choose careers in academic medicine? A literature review. *Acad Med* 2010;85(4):680-6. doi: 10.1097/ACM.0b013e3181d29cb9

21 Department of Health. *NHS Constitution for England*. 27 July 2015. www.gov.uk/government/publications/the-nhs-constitution-for-england (accessed 2 Nov 2016).

22 Ozdemir BA, Karthikesalingam A, Sinha S, et al. Research Activity and the Association with Mortality. *PLoS ONE* 2015;10(2):e0118253. doi:10.1371/journal.pone.0118253

23 Boaz A, Hanney S, Jones T, *et al.* Does the engagement of clinicians and organisations in research improve healthcare performance: a three-stage review. *BMJ Open* 2015;5:e009415. doi:10.1136/bmjopen-2015-009415

For peer review only - http://bmjopen.bmj.com/site/about/guidelines.xhtml

The clinical academic workforce of the future: a cross-sectional study of factors influencing career decision-making amongst clinical PhD students at two research-intensive UK universities

SUPPLEMENTARY INFORMATION

Box 1 – Questions used

Box 2 – Gender and fee status of clinical doctoral students: percentages by university and for all respondents

Box 3 – Respondents' predoctoral involvement in medical research and reason for the doctorate: percentages by gender and for all respondents

Box 4 – Variables not associated with wanting a clinical academic career (CAC) *vs.* having other plans or being undecided: statistically *non*-significant X^2 tests

Box 5 – Specialties classified as ''craft'' and ''non-craft'' specialties

Box 6 – Factors influencing respondents' thinking about their long-term career: percentages for those who want a clinical academic career (CAC) and those who do not/are undecided, and for all respondents

Box 7 – Variables not associated with likelihood of seeking a CL: statistically *non*-significant X^2 tests

Box 8 – Sources of information about possible career paths: by gender for those who want a clinical academic career (CAC) and those have other plans/are undecided, and for all respondents

For peer review only

Box 1 – Questions used

For its survey, UCL made minor adjustments to the wording of questions, such as using the term ''PhD'' instead of ''DPhil''.

1. Medical training

1.A. Which level of training are you currently in? (*Tick one*)

1. Foundation programme (or equivalent) **[→ SKIP Questions 1.B. & 1.C.]**
2. Core training (or equivalent) **[→ SKIP Questions 1.B. & 1.C.]**
3. Acute Care Common Stem (or equivalent) **[→ SKIP Questions 1.B. & 1.C.]**
4. Specialty training (or equivalent) **[→ Go to Question 1.C.]**
5. NOT APPLICABLE: I am between levels of training **[→ Go to Question 1.B.]**
6. NOT APPLICABLE: I have left training and do not plan to return **[→ Go to Question 1.B.]**
7. NOT APPLICABLE: I have fully completed my training **[→ SKIP Questions 1.B. & 1.C.]**

1.B. What is the highest stage of clinical training which you have FULLY COMPLETED so far? (*Tick one*)

1. Medical School **[→ SKIP Question 1.C.]**
2. Foundation Programme (or equivalent) **[→ SKIP Question 1.C.]**
3. Core Training (or equivalent) **[→ SKIP Question 1.C.]**
4. Acute Care Common Stem (or equivalent) **[→ SKIP Question 1.C.]**
5. Specialty Training (or equivalent) **[→ SKIP Question 1.C.]**

1.C. Please specify your Specialty Training level. (*Enter ST or SpR, followed by a number 1 to 10*)**:**
[text box]

1.D. What is, or will be, your medical specialty? *If you have not yet decided, please enter 'not applicable'.*
[text box]

For peer review only - http://bmjopen.bmj.com/site/about/guidelines.xhtml

Box 1 (cont.)

2. Prior involvement in research

2.A. What involvement in medical research did you have prior to your [DPhil/PhD]? (*Tick all that apply*)

1. No involvement
2. Intercalated degree
3. Research rotation as an Academic Foundation Programme doctor
4. Research block or regular release as an Academic Clinical Fellow (England, Wales, Northern Ireland)
5. Research block or regular release as a Clinical Research Fellow (Scotland)
6. Other post with time for research
7. Other (please specify)

3. DPhil / PhD

3.A. What was your MAIN reason for choosing to do a [DPhil/PhD]? (*Tick one*)

1. Interest in my area of research
2. Improving my prospects for an academic/research career
3. Improving my prospects for a clinical career outside academia
4. Other reason (please specify)

3.B. Overall, how much encouragement do you receive from your [DPhil/PhD] supervisor(s) to reflect on your career development needs? (*Tick one*)
Response options: A great deal; A lot; A moderate amount; A little; None at all

For peer review only

Box 1 (cont.)

4. Career paths

4.A. In your long-term career, in what type of posts do you mainly intend to work? (*Tick one*)

1. Clinical academic posts **[→ Skip Question 4.B.]**
2. Clinical service posts with some teaching and research
3. Clinical service posts with some research
4. Clinical service posts with some teaching
5. Clinical service posts without teaching or research
6. Research-only posts **[→ Skip Question 4.B.]**
7. Undecided
8. Other (please specify)

4.B. How much would each of the following be a reason for excluding a clinical academic career from your long-term career plans? (*Tick one per row*)
Response options: A great deal; A lot; A moderate amount; A little; Not at all; Not applicable to me

1. The difficulty of obtaining research grants
2. The small number of senior academic appointments available
3. The competing pressures from service, teaching, and research
4. The limited future financial prospects compared to alternative careers
5. My perception of less favourable conditions of employment compared with NHS colleagues (in respects other than salary)
6. The possibility of a significantly longer training period to completion of training compared with NHS colleagues
7. Lack of exit paths if pursuing an academic career path and then changing my mind
8. Needing work arrangements that enable me to meet caring responsibilities (for children and/or adults)
9. Family circumstances making me unwilling to move for work
10. Realising that research is not for me
11. Other reasons (please specify)

For peer review only

Box 1 (cont.)

4.C. How much does each of the following factors influence your thinking about your long-term career? (*Tick one per row*)
Response options: A great deal; A lot; A moderate amount; A little; Not at all

1. Wanting a career that fits my domestic circumstances
2. Future financial prospects
3. Wanting a career with acceptable working hours/working conditions
4. Advice from others
5. Wanting intellectual stimulation
6. Wanting to make a difference to patients
7. Lack of posts in other possible career paths
8. Wanting to do what I really want to do (enthusiasm/commitment)
9. Self-appraisal of own skills/aptitudes
10. Career promotion and prospects
11. Exposure to role models
12. Other (please give details)

4.D. What might make a clinical academic career more attractive to you?
[text box]

4.E. How well do you feel your [DPhil/PhD] programme will/would prepare you for a clinical academic career, if this is/was your chosen career path? (*Tick one*)
Response options: Extremely well; Very well; Moderately well; Slightly well; Not at all well

4.F. How much information do you get about possible career paths from each of the following? (*Tick one per row*)
Response options: A great deal; A lot; A moderate amount; A little; None at all; Not applicable to me

1. My academic/research supervisor(s)
2. Senior academics in my department
3. My peers undertaking a [DPhil/PhD] at the [University of Oxford / UCL]
4. [OUCAGS / ACO]
5. Mentor(s)
6. Newsletters/publications from medical bodies (e.g. GMC, NIHR)
7. Other (please specify)

For peer review only

Box 1 (cont.)

5. Clinical Lectureships

5.A. Please tell us how accurately the following statement describes you: 'If I wanted, I could become a Clinical Lecturer in England, at an appropriate point after completing my [DPhil/PhD]'. (*Tick one*)
Response options: Extremely accurately; Very accurately; Moderately accurately; Slightly accurately; Not at all accurately

Note: respondents who said that, in their long-term career, they intended to work mainly in ''clinical service posts without teaching or research''@ Question 4.A. →**SKIP Question 5.B..**

5.B. How likely are you to seek a Clinical Lecturer post in England, at an appropriate point after you complete your [DPhil/PhD]? (*Tick one*)
Response options: Extremely likely; Very likely; Moderately likely; Slightly likely; Not at all likely

5.C. How much do you feel that you know about Clinical Lecturer posts in England (including how to apply and what the posts entail)? (*Tick one*)
Response options: A great deal; A lot; A moderate amount; A little; Nothing at all

6. About respondents

6.A. What is your sex? (*Tick one*)
Response options: Male; Female

Note: To check whether respondents were representative of the whole population, we used administrative data about the whole population of clinical doctoral students invited to respond to our questionnaire. At the University of Oxford, only administrative data about clinical doctoral students' sex (collected as ''male'' or ''female'') was available for use in our study. Data on gender was not available. To ensure that data from our questionnaire would be comparable to the administrative data, we asked participants about their sex rather than their gender. However, in the presentation and discussion of results, we opted to refer to data on whether respondents ticked themselves as being male or female as ''gender'' because this, not their biological sex, is the relevant issue.

6.B. In which year were you born?
[text box: ''19_______'']

6.C. Do you have a mentor, or mentors, who you meet regularly? By mentor we mean someone who is not your line manager or supervisor, and who helps you develop your knowledge, work or thinking. (*Tick one*)

1. I have a mentor/mentors, as part of a formalised mentoring relationship
2. I have an informal mentor/mentors
3. I do not have a mentor, but would like to find one
4. I do not have a mentor and do not feel the need, or have the time, for one
5. Other (please specify)

For peer review only

Box 1 (cont.)

6.D. For fees purposes, what is your normal place of residence registered as? (*Tick one*)
Response options: Home (UK); Other European Union; Non-European Union

6.E. In which calendar year did you start your [DPhil/PhD] programme? (*Tick one*)
Response options: 2011 or earlier; 2012; 2013; 2014; 2015

For peer review only

Box 2 – Gender and fee status of clinical doctoral students: percentages by university and for all respondents

	Oxford (N=170)		UCL (N=150)		Total (N=320)	
**Gender**	**n**	***%***	**n**	***%***	**n**	***%***
Male	108	***64***	66	***44***	174	***54***
Female	61	***36***	81	***54***	142	***44***
No response	1	***1***	3	***2***	4	***1***
**Fee status**	**n**	***%***	**n**	***%***	**n**	***%***
UK/EU	131	***77***	148	***99***	279	***87***
Overseas	33	***19***	-	***-***	33	***10***
No response	6	***4***	2	***1***	8	***3***

Note: Due to rounding, percentages may not add to 100.

For peer review only

Box 3 – Respondents' predoctoral involvement in medical research and reason for the doctorate: percentages by gender and for all respondents

	Males⸸ (N=174)		Females⸸ (N=142)		Total (N=320)	
	n	%	n	%	n	%
**Previous involvement in medical research⸸⸸**						
High involvement level *(intercalated degree and post(s) with research*, and possibly "other" experience, too)*	34	20	28	20	62	19
Some involvement *(intercalated degree or post(s) with research*, possibly with other experience, or "other" experience)*	112	64	91	64	206	64
No involvement	22	13	15	11	38	12
No response	6	3	8	6	14	4
**Main reason for doctorate**						
Interest in area of research	85	49	60	42	146	46
Improving prospects for an academic/research career	70	40	61	43	134	42
Improving prospects for a clinical career outside academia	16	9	17	12	33	10
Other	3	2	4	3	7	2

Note: Due to rounding, column percentages may not add to 100.
⸸Four respondents did not provide their gender.
⸸⸸ More than one answer could be given to the question about involvement in medical research prior to the DPhil/PhD – see Box 1, Question 2.A..
*The following response options were considered to be 'posts with research': research rotation as an Academic Foundation Programme doctor; research block or regular release as an Academic Clinical Fellow (England, Wales, Northern Ireland); research block or regular release as a Clinical Research Fellow (Scotland); other post with time for research.

For peer review only

Box 4 – Variables not associated with wanting a clinical academic career (CAC) *vs.* having other plans or being undecided: statistically *non*-significant X^2 tests

		Respondents wanting a CAC		X^2 test		
**Variables**	**Groupings**	**%**	**n / N**	**df**	**X^2**	**p**
**Gender**	Male	55	96 / 174	1	3.430	.064
	Female	45	63 / 141			
**Fee status**	UK/EU	50	139 / 278	1	.027	.869
	Non-EU (Overseas)	52	17 / 33			
**Age**	<30 years	49	22 / 45	2	3.370	.185
	30-35 years	55	96 / 174			
	36 years and over	40	19 / 47			
**Training stage**	Pre-specialty	63	24 / 38	2	3.072	.215
	In specialty	48	108 / 224			
	Completed	47	27 / 57			
**Specialty‡**	Craft	42	25 / 60	2	3.903	.142
	Non-craft	52	123 / 235			
	Not yet selected	35	7 / 20			
**Encouragement from DPhil/PhD supervisor(s) to reflect on career development needs**	A great deal/A lot	53	79 / 150	2	.927	.629
	A moderate amount	47	44 / 94			
	A little/None at all	48	36 / 75			
Influences on long-term career thinking:						
**Wanting a career that fits my domestic circumstances**	A great deal/A lot	51	89 / 175	2	.733	.693
	A moderate amount	51	53 / 105			
	A little/Not at all	43	16 / 37			
**Wanting to make a difference to patients**	A great deal/ A lot	52	140 / 267	2	3.951	.139
	A moderate amount	38	15 / 40			
	A little/ Not at all	36	4 / 11			
**Wanting a career with acceptable working hours/conditions**	A great deal/A lot	46	80 / 174	2	2.494	.287
	A moderate amount	55	60 / 109			
	A little/Not at all	54	19 / 35			
**Future financial prospects**	A great deal/A lot	51	62 / 122	2	.457	.796
	A moderate amount	51	64 / 125			
	A little/Not at all	47	33 / 71			
**Advice from others**	A great deal/A lot	57	39 / 69	2	3.602	.165
	A moderate amount	51	84 / 164			
	A little/Not at all	42	35 / 84			
**Exposure to role models**	A great deal/A lot	55	85 / 156	2	2.668	.263
	A moderate amount	46	48 / 105			
	A little/Not at all	45	25 / 56			

For peer review only

Box 4 *(continued)*

		Respondents wanting a CAC		X^2 test		
**Variables**	**Groupings**	**%**	**n / N**	**df**	**X^2**	**p**
Sources of information about possible career paths (amount of information received):						
**Academic/research supervisor(s)**	A great deal/A lot	55	57 / 104	2	2.123	.346
	A moderate amount	51	55 / 109			
	A little/None at all/N/A	45	47 / 105			
**Senior academics in department**	A great deal/A lot	57	36 / 63	2	5.557	.062
	A moderate amount	56	53 / 94			
	A little/None at all/N/A	44	70 / 161			
**Peers at same university**	A great deal/A lot	55	36 / 66	2	1.388	.500
	A moderate amount	52	56 / 108			
	A little/None at all/N/A	47	67 / 144			
**Newsletters /publications**	A great deal/A lot	54	15 / 28	2	2.589	.274
	A moderate amount	59	37 / 63			
	A little/None at all/N/A	48	107 / 225			
Mentoring:						
**Formal/informal mentor, regularly met**	Yes	51	91 / 180	1	.052	.820
	No	49	67 / 136			

ǂSee Box 5 for details of respondents' specialties.

For peer review only

Box 5 – Specialties classified as "craft" and "non-craft" specialties

With regard to specialty, most respondents were in non-craft specialties (74%, n=236), and about a fifth (19%, n=60) in a craft specialty. The remaining respondents had not yet committed to a specialty, did not respond, or it was not possible to classify their response.

Specialties were classified as follows:

**Craft specialties**	**Non-craft specialties**	
Obstetrics and gynaecology	Acute internal medicine	Neonatal medicine
Gynaecological oncology	Allergy	Neurology
Urogynaecology	Anaesthetics	Nuclear medicine
Ophthalmology	Audio-vestibular medicine	Occupational medicine
Interventional radiology	Cardiology	Paediatric allergy, immunology and infectious diseases
Surgery (any type)	Chemical pathology	Paediatric and perinatal pathology
Otolaryngology	Child mental health	Paediatric cardiology
Urology	Clinical genetics	Paediatric clinical pharmacology and therapeutics
	Clinical neurophysiology	Paediatric diabetes and endocrinology
	Clinical oncology	Paediatric emergency medicine
	Clinical pharmacology and therapeutics	Paediatric gastro-enterology, hepatology and nutrition
	Clinical radiology	Paediatric inherited metabolic medicine
	Community child health	Paediatric intensive care medicine
	Cytopathology	Paediatric nephrology
	Dermatology	Paediatric neurodisability
	Diagnostic neuropathology	Paediatric neurology
	Emergency medicine	Paediatric oncology
	Endocrinology and diabetes mellitus	Paediatric palliative medicine
	Forensic histopathology	Paediatric respiratory medicine
	Gastro-enterology	Paediatric rheumatology
	General (internal) medicine	Paediatrics
	General practice	Palliative medicine
	Genito-urinary medicine	Pharmaceutical medicine
	Geriatric medicine	Pre-hospital emergency medicine
	Haematology	Psychiatry (any type)
	Hepatology	Public health medicine
	Histopathology	Rehabilitation medicine
	Immunology	Renal medicine
	Infectious diseases	Respiratory medicine
	Intensive care medicine	Rheumatology
	Medical microbiology	Sport and exercise medicine
	Medical microbiology and virology	Stroke medicine
	Medical oncology	Tropical medicine
	Medical ophthalmology	
	Medical psychotherapy	
	Medical virology	
	Metabolic medicine	

For peer review only

For peer review only - http://bmjopen.bmj.com/site/about/guidelines.xhtml

Box 6 – Factors influencing respondents' thinking about their long-term career: percentages for those who want a clinical academic career (CAC) and those who do not/are undecided, and for all respondents

	Respondents wanting a CAC‡				Respondents *not* wanting a CAC‡				Total (N=320)	
	Males (N=96)		Females (N=63)		Males (N=78)		Females (N=78)			
	n	*%*	*n*	*%*	*n*	*%*	*n*	*%*	n	*%*
**Enthusiasm/commitment**										
A great deal/A lot	94	***98***	62	***98***	71	***91***	70	***90***	301	***94***
(A great deal)*	(67)	***(70)***	(40)	***(64)***	(38)	***(49)***	(43)	***(55)***	(191)	***(60)***
A moderate amount	2	***2***	1	***2***	6	***8***	7	***9***	17	***5***
A little/Not at all	0	***0***	0	***0***	0	***0***	1	***1***	1	***<1***
No response	-	-	-	-	1	***1***	-	-	1	***<1***
**Wanting intellectual stimulation**										
A great deal/A lot	95	***99***	62	***98***	61	***78***	63	***81***	285	***89***
(A great deal)*	(60)	***(63)***	(40)	***(64)***	(28)	***(36)***	(31)	***(40)***	(160)	***(50)***
A moderate amount	1	***1***	1	***2***	15	***19***	14	***18***	32	***10***
A little/Not at all	0	***0***	0	***0***	1	***1***	1	***1***	2	***1***
No response	-	-	-	-	1	***1***	-	-	1	***<1***
**Wanting to make a difference to patients**										
A great deal/A lot	82	***85***	58	***92***	59	***76***	65	***83***	268	***84***
(A great deal)*	(52)	***(54)***	(29)	***(46)***	(30)	***(39)***	(36)	***(46)***	(148)	***(46)***
A moderate amount	11	***12***	4	***6***	14	***18***	10	***13***	40	***13***
A little/Not at all	3	***3***	1	***2***	4	***5***	3	***4***	11	***3***
No response	-	-	-	-	1	***1***	-	-	1	***<1***
**Self-appraisal of own skills/aptitudes**										
A great deal/A lot	63	***66***	44	***70***	37	***47***	46	***59***	192	***60***
A moderate amount	23	***24***	15	***24***	32	***41***	25	***32***	98	***31***
A little/Not at all	10	***10***	4	***6***	8	***10***	7	***9***	29	***9***
No response	-	-	-	-	1	***1***	-	-	1	***<1***
**Wanting a career that fits my domestic circumstances**										
A great deal/A lot	48	***50***	41	***65***	38	***49***	46	***59***	176	***55***
A moderate amount	36	***38***	17	***27***	25	***32***	26	***33***	105	***33***
A little/Not at all	11	***12***	5	***8***	14	***18***	6	***8***	37	***12***
No response	1	***1***	-	-	1	***1***	-	-	2	***1***
**Wanting a career with acceptable working hours/conditions**										
A great deal/A lot	42	***44***	38	***60***	38	***49***	54	***69***	174	***54***
A moderate amount	41	***43***	19	***30***	28	***36***	19	***24***	110	***34***
A little/Not at all	13	***14***	6	***10***	11	***14***	5	***6***	35	***11***
No response	-	-	-	-	1	***1***	-	-	1	***<1***

For peer review only - http://bmjopen.bmj.com/site/about/guidelines.xhtml

Box 6 (continued)

	Respondents wanting a CAC‡				Respondents *not* wanting a CAC‡				Total (N=320)	
	Males (N=96)		Females (N=63)		Males (N=78)		Females (N=78)			
	n	*%*	*n*	*%*	*n*	*%*	*n*	*%*	n	*%*
**Exposure to role models**										
A great deal/A lot	52	***54***	33	***52***	34	***44***	35	***45***	157	***49***
A moderate amount	29	***30***	19	***30***	27	***35***	28	***36***	105	***33***
A little/Not at all	14	***15***	11	***18***	16	***21***	15	***19***	56	***18***
No response	1	***1***	-	-	1	***1***	-	-	2	***1***
**Career promotion and prospects**										
A great deal/A lot	53	***55***	32	***51***	35	***45***	25	***32***	146	***46***
A moderate amount	28	***29***	23	***37***	26	***33***	38	***49***	119	***37***
A little/Not at all	15	***16***	8	***13***	16	***21***	15	***19***	54	***17***
No response	-	-	-	-	1	***1***	-	-	1	***<1***
**Future financial prospects**										
A great deal/A lot	41	***43***	21	***33***	33	***42***	25	***32***	122	***38***
A moderate amount	39	***41***	25	***40***	26	***33***	34	***44***	126	***39***
A little/Not at all	16	***17***	17	***27***	18	***23***	19	***24***	71	***22***
No response	-	-	-	-	1	***1***	-	-	1	***<1***
**Advice from others**										
A great deal/A lot	23	***24***	16	***25***	19	***24***	11	***14***	69	***22***
A moderate amount	51	***53***	33	***52***	34	***44***	42	***54***	164	***51***
A little/Not at all	21	***22***	14	***22***	24	***31***	25	***32***	84	***26***
No response	1	***1***	-	-	1	***1***	-	-	3	***1***
**Lack of posts in other possible career paths**										
A great deal/A lot	7	***7***	1	***2***	8	***10***	13	***17***	30	***9***
A moderate amount	9	***9***	10	***16***	15	***19***	19	***24***	53	***17***
A little/Not at all<br>(Not at all)*	80<br>(47)	***83***<br>***(49)***	52<br>(43)	***83***<br>***(68)***	54<br>(29)	***69***<br>***(37)***	46<br>(26)	***59***<br>***(33)***	236<br>(147)	***74***<br>***(46)***
No response	-	-	-	-	1	***1***	-	-	1	***<1***

Notes: Due to rounding, column percentages may not add to 100. A minority of respondents rated “other” factors as part of the final, open item of the question; as no recurrent themes were identified, this item is not included in the table.
‡Base: All respondents in group who provided their gender and responded to the question about their career plans (N=315).
*Some variables were considerably skewed. Where the mode for the whole group of respondents (total) was either ‘‘a great deal’’ or ‘‘not at all’’, figures for the modal response-only are provided in brackets.

For peer review only

Box 7 – Variables not associated with likelihood of seeking a CL: statistically *non*-significant X^2 tests

Variables	Groupings	Respondents extremely/very likely to seek a CL		X^2 test		
		%	n / N	df	X^2	p
**Gender**	Male	46	78 / 171	1	.788	.375
	Female	41	56 / 138			
**Specialty**	Craft	41	24 / 58	2	5.911	.052
	Non-craft	44	103 / 232			
	Not yet selected	16	3 / 19			
**Encouragement from DPhil/PhD supervisor(s) to reflect on career development needs**	A great deal/A lot	48	70 / 147	2	2.832	.243
	A moderate amount	37	34 / 92			
	A little/None at all	41	30 / 74			
Sources of information about possible career paths (amount of information received):						
**Peers**	A great deal/A lot	44	29 / 66	2	2.626	.269
	A moderate amount	49	51 / 105			
	A little/None at all/N/A	38	54 / 141			
**Newsletters/publications**	A great deal/A lot	50	14 / 28	2	1.312	.519
	A moderate amount	47	29 / 62			
	A little/None at all/N/A	41	90 / 220			
Mentoring:						
**Formal/informal mentor, regularly met**	Yes	44	77 / 177	1	.013	.910
	No	43	57 / 133			

For peer review only

Box 8 – Sources of information about possible career paths: by gender for those who want a clinical academic career (CAC) and those who have other plans/are undecided, and for all respondents

	Respondents wanting a CACǂ				Respondents *not* wanting a CACǂ				Total (N=320)	
	Males (N=96)		Females (N=63)		Males (N=78)		Females (N=78)			
	n	%	*n*	*%*	*n*	*%*	*n*	*%*	n	*%*
**Academic/research supervisor(s)**										
Great deal/A lot	39	**41**	*18*	**29**	23	***30***	*23*	***30***	105	***33***
A moderate amount	29	**30**	*26*	**41**	32	***41***	*21*	***27***	109	***34***
A little/None/N/A	28	**29**	*19*	**30**	23	***30***	*33*	***42***	105	***33***
No response	-	-	-	-	-	-	*1*	***1***	1	***<1***
**Senior academics in my department**										
Great deal/A lot	27	**28**	9	**14**	17	***22***	9	***12***	63	***20***
A moderate amount	29	**30**	24	**38**	22	***28***	18	***23***	94	***29***
A little/None/N/A	40	**42**	30	**48**	39	***50***	50	***64***	162	***51***
No response	-	-	-	-	-	-	1	***1***	1	***<1***
**Peers undertaking a doctorate at Oxford/UCL**										
Great deal/A lot	29	**30**	7	**11**	19	***24***	11	***14***	66	***21***
A moderate amount	33	**34**	23	**37**	24	***31***	27	***35***	108	***34***
A little/None/N/A	34	**35**	33	**52**	35	***45***	39	***50***	145	***45***
No response	-	-	-	-	-	-	1	***1***	1	***<1***
**OUCAGS/ACO**										
Great deal/A lot	13	**14**	9	**14**	3	***4***	2	***3***	27	***8***
A moderate amount	21	**22**	12	**19**	11	***14***	7	***9***	52	***16***
A little/None/N/A	61	**64**	42	**67**	64	***82***	65	***83***	236	***74***
(Not applicable) *	(4)	**(4)**	(6)	**(10)**	(12)	***(15)***	(4)	***(5)***	(26)	***(8)***
No response	1	**1**	-	-	-	-	4	***5***	5	***2***
**Mentors**										
Great deal/A lot	28	**29**	22	**35**	18	***23***	10	***13***	78	***24***
A moderate amount	28	**29**	12	**19**	25	***32***	16	***21***	81	***25***
A little/None/N/A	39	**41**	29	**46**	35	***45***	49	***63***	157	***49***
(Not applicable) *	(15)	**(16)**	(15)	**(24)**	(11)	***(14)***	(17)	***(22)***	(60)	***(19)***
No response	1	**1**	-	-	-	-	3	***4***	4	***1***
**Newsletters/publications from medical bodies**										
Great deal/A lot	11	**12**	4	**6**	6	***8***	6	***8***	28	***9***
A moderate amount	23	**24**	14	**22**	11	***14***	14	***18***	64	***20***
A little/None/N/A	62	**65**	45	**71**	61	***78***	55	***71***	225	***70***
No response	-	-	-	-	-	-	3	***4***	3	***1***

Note: Due to rounding, column percentages may not add to 100.
ǂBase: All respondents in group who provided their gender and responded to the question about their career plans (N=315).
**Question attracted ''not applicable'' responses from over 3% of respondents, so ''not applicable''-only responses are provided separately in brackets.*

For peer review only - http://bmjopen.bmj.com/site/about/guidelines.xhtml

STROBE Statement—Checklist of items that should be included in reports of ***cross-sectional studies***

	Item No	Recommendation
**Title and abstract**	1	(*a*) Indicate the study's design with a commonly used term in the title or the abstract [p.1 , in *Title*; p.2, in *Abstract*]
		(*b*) Provide in the abstract an informative and balanced summary of what was done and what was found [p.2, *Abstract*]
**Introduction**		
Background/rationale	2	Explain the scientific background and rationale for the investigation being reported [p.4, *Introduction*]
Objectives	3	State specific objectives, including any prespecified hypotheses [p.4, *Introduction*]
**Methods**		
Study design	4	Present key elements of study design early in the paper [p.5, *Method – Design and procedures*]
Setting	5	Describe the setting, locations, and relevant dates, including periods of recruitment, exposure, follow-up, and data collection [p.5, *Method – Design and procedures*]
Participants	6	(*a*) Give the eligibility criteria, and the sources and methods of selection of participants [p.5, *Method – Design and procedures*]
Variables	7	Clearly define all outcomes, exposures, predictors, potential confounders, and effect modifiers. Give diagnostic criteria, if applicable [p.5, *Method – Instruments*]
Data sources/ measurement	8*	For each variable of interest, give sources of data and details of methods of assessment (measurement). Describe comparability of assessment methods if there is more than one group [p.5, *Method – Instruments*]
Bias	9	Describe any efforts to address potential sources of bias [n/a: respondents are representative by gender of the population, as per p.7, *Results – Characteristics of clinical doctoral students: Demographics and training*]
Study size	10	Explain how the study size was arrived at [p.5, *Method – Design and procedures*]
Quantitative variables	11	Explain how quantitative variables were handled in the analyses. If applicable, describe which groupings were chosen and why [pp.5-6, *Method – Analysis*]
Statistical methods	12	(*a*) Describe all statistical methods, including those used to control for confounding [pp.5-6, *Method – Analysis*]
		(*b*) Describe any methods used to examine subgroups and interactions [pp.5-6, *Method – Analysis*]
		(*c*) Explain how missing data were addressed [n/a]
		(*d*) If applicable, describe analytical methods taking account of sampling strategy [n/a]
		(*e*) Describe any sensitivity analyses [n/a]

For peer review only

**Results**		
Participants	13*	(a) Report numbers of individuals at each stage of study—eg numbers potentially eligible, examined for eligibility, confirmed eligible, included in the study, completing follow-up, and analysed [p.5, *Method – Design and procedures* and p.7; *Results – Characteristics of clinical doctoral students: Response rates*]
		(b) Give reasons for non-participation at each stage [p.7, *Results – Characteristics of clinical doctoral students: Response rates*]
		(c) Consider use of a flow diagram [n/a]
Descriptive data	14*	(a) Give characteristics of study participants (eg demographic, clinical, social) and information on exposures and potential confounders [p.7, *Results – Characteristics of clinical doctoral students*]
		(b) Indicate number of participants with missing data for each variable of interest [p.7, *Results – Characteristics of clinical doctoral students: Demographics and training*; p.9, Table 1; p.14, Table 4]
Outcome data	15*	Report numbers of outcome events or summary measures [*Results*: p.9, Table 1; p.14, Table 4]
Main results	16	(*a*) Give unadjusted estimates and, if applicable, confounder-adjusted estimates and their precision (eg, 95% confidence interval). Make clear which confounders were adjusted for and why they were included [n/a]
		(*b*) Report category boundaries when continuous variables were categorized [n/a]
		(*c*) If relevant, consider translating estimates of relative risk into absolute risk for a meaningful time period [n/a]
Other analyses	17	Report other analyses done—eg analyses of subgroups and interactions, and sensitivity analyses [*Results*: p.11, Table 2; p.15, Table 5]
**Discussion**		
Key results	18	Summarise key results with reference to study objectives [p.17, *Discussion*]
Limitations	19	Discuss limitations of the study, taking into account sources of potential bias or imprecision. Discuss both direction and magnitude of any potential bias [p.18, *Discussion – Strengths and limitations of the study*]
Interpretation	20	Give a cautious overall interpretation of results considering objectives, limitations, multiplicity of analyses, results from similar studies, and other relevant evidence [pp.17-19, *Discussion*]
Generalisability	21	Discuss the generalisability (external validity) of the study results [p.18, *Discussion –Strengths and limitations of the study*]
**Other information**		
Funding	22	Give the source of funding and the role of the funders for the present study and, if applicable, for the original study on which the present article is based [p.20]

*Give information separately for exposed and unexposed groups.

For peer review only

For peer review only - http://bmjopen.bmj.com/site/about/guidelines.xhtml

Note: An Explanation and Elaboration article discusses each checklist item and gives methodological background and published examples of transparent reporting. The STROBE checklist is best used in conjunction with this article (freely available on the Web sites of PLoS Medicine at http://www.plosmedicine.org/, Annals of Internal Medicine at http://www.annals.org/, and Epidemiology at http://www.epidem.com/). Information on the STROBE Initiative is available at www.strobe-statement.org.

For peer review only

The clinical academic workforce of the future: a cross-sectional study of factors influencing career decision-making amongst clinical PhD students at two research-intensive UK universities

Journal:	*BMJ Open*
Manuscript ID	bmjopen-2017-016823.R1
Article Type:	Research
Date Submitted by the Author:	08-Jun-2017
Complete List of Authors:	Lopes, Joana; University of Oxford, Oxford University Clinical Academic Graduate School<br>Ranieri, Veronica; University College London, School of Life and Medical Sciences; University College London, Department of Applied Health Research<br>Lambert, Trevor; University of Oxford, Nuffield Department of Population Health<br>Pugh, Chris; University of Oxford, Oxford University Clinical Academic Graduate School<br>Barratt, Helen; University College London, Department of Applied Health Research<br>Fulop, Naomi; University College London, Department of Applied Health Research<br>Rees, Geraint; University College London, School of Life and Medical Sciences<br>Best, Denise; University of Oxford, Oxford University Clinical Academic Graduate School
**Primary Subject Heading**:	Medical education and training
Secondary Subject Heading:	Evidence based practice
Keywords:	EDUCATION & TRAINING (see Medical Education & Training), MEDICAL EDUCATION & TRAINING, Human resource management < HEALTH SERVICES ADMINISTRATION & MANAGEMENT

SCHOLARONE™
Manuscripts

For peer review only - http://bmjopen.bmj.com/site/about/guidelines.xhtml

The clinical academic workforce of the future: a cross-sectional study of factors influencing career decision-making amongst clinical PhD students at two research-intensive UK universities

Joana Lopes[1], Veronica Ranieri[2 3], Trevor Lambert[4], Chris Pugh[1], Helen Barratt[3], Naomi J Fulop[3], Geraint Rees[2], Denise Best[1]

[1]Oxford University Clinical Academic Graduate School, Room 3600 Medical Sciences Divisional Office, Level 3, John Radcliffe Hospital (Main Hospital), Oxford, OX3 9DU, UK
[2]School of Life and Medical Sciences, University College London, Gower Street, London WC1E 6BT, UK
[3] Department of Applied Health Research, University College London, Gower Street, London WC1E 6BT, UK
[4]Nuffield Department of Population Health, University of Oxford, Oxford OX3 7LF, UK

Correspondence to: Joana Lopes; joana.lopes@medsci.ox.ac.uk; T: 01865 289 465

Word count: 4,068

For peer review only

ABSTRACT

Objectives To examine clinical doctoral students' demographic and training characteristics, career intentions, career preparedness, and what influences them as they plan their future careers.

Design and setting Online cross-sectional census surveys at two research-intensive medical schools in England in 2015-16.

Participants All medically qualified PhD students (N=523) enrolled at the University of Oxford and University College London were invited to participate. We report on data from 320 participants (54% male and 44% female), who were representative by gender of the invited population.

Main outcome measures Career intentions.

Results Respondents were mainly in specialty training, including close to training completion (25%, n=80), and 18% (n=57) had completed training. Half (50%, n=159) intended to pursue a clinical academic career (CAC) and 62% (n=198) were at least moderately likely to seek a clinical lectureship (CL). However, 51% (n=163) had little or no knowledge about CL posts. Those wanting a CAC tended to have the most predoctoral medical research experience [X^2 (2, N=305) = 22.19, p=.0005]. Key reasons cited for not pursuing a CAC were the small number of senior academic appointments available, the difficulty of obtaining research grants, and work-life balance.

Conclusions Findings suggest that urging predoctoral clinicians to gain varied research experience whilst ensuring availability of opportunities, and introducing more flexible recruitment criteria for CL appointments, would foster CACs. As CL posts are often only open to those still in training, the many postdoctoral clinicians who have completed training, or nearly done so, do not currently gain the opportunity the post offers to develop as independent researchers. Better opportunities should be accompanied by enhanced career support for clinical doctoral students (e.g. to increase knowledge of CLs). Finally, ways to increase the number of senior clinical academic appointments should be explored since their lack seems to significantly influence career decisions.

Key words: clinical academic, academic medicine, clinical lectureship, physician scientist, career development

For peer review only - http://bmjopen.bmj.com/site/about/guidelines.xhtml

For peer review only

Strengths and limitations of this study

- This is the first UK study to describe the career aspirations of medically qualified PhD students and what influences them as they make their postdoctoral career plans.
- Other studies about the retention of postdoctoral clinical academics are predominantly North American and reliant on retrospective data.
- The study identifies factors which are associated with clinical PhD students' decision to apply for clinical lectureships (a pivotal post in clinical academic training in England).
- As it is a cross-sectional study, causality cannot be inferred from the associations we found between career plans and other variables.
- It is not possible to say whether respondents' intentions will be implemented as planned and findings may not generalise beyond research-intensive universities in England.

For peer review only

For peer review only - http://bmjopen.bmj.com/site/about/guidelines.xhtml

INTRODUCTION

Clinical academics (also known internationally as physician scientists) are vital to educating medical students and, as they bring a clinical perspective to research, to discovering and realising new insights into human disease.[1] Establishing a stable pipeline that develops clinical academics,[2] continues to be of global concern, for example in the US and Australia.[3,4] In the UK, anxieties date back at least to the Savill report[1] in 2000 and the subsequent Modernising Medical Careers report.[5] The establishment of the National Institute for Health Research (NIHR) in 2006, and similar developments in Scotland, Wales, and Northern Ireland, supported the formation of an integrated clinical academic training (IAT) pathway[6] designed to tackle concerns about dwindling clinical academic numbers by providing clinical training posts with protected research time. In England, clinical academic training opportunities start with Academic Foundation posts, continuing to predoctoral academic clinical fellowships (ACFs), doctoral training fellowships and, finally, postdoctoral clinical lectureships (CLs).[6] Despite these efforts, the latest report from the Medical Schools Council[7] states that more than half of UK medical schools report difficulties in recruitment to some clinical academic posts. Of additional concern is the small proportion of women who pursue clinical academic careers (CACs).[8]

A pivotal point common to clinical academic training pathways irrespective of country is the PhD.[6] Interestingly, recent career tracking of doctoral clinical training fellowships awarded by major UK research funders[9] suggests that only around a third of holders progress to a junior postdoctoral CL or the more senior clinician scientist post. (CLs are open to doctors in specialty training, whereas clinician scientists have completed their training and developed research independence.) A range of barriers and enablers have been identified as contributing to this issue, including matters relating to work-life balance, funding, mentorship and careers information.[9-12] Notwithstanding, there has been no work in the UK examining the characteristics of doctors undertaking PhDs or how they view their future career options, including why those wanting a CAC may not consider applying for a postdoctoral CL.[10,13]

In 2013, the Oxford University Clinical Academic Graduate School (OUCAGS) began a longitudinal study of its clinical doctoral students to help understand these issues. The part of the study reported here was undertaken in 2015-16 in collaboration with University College London's (UCL) Academic Careers Office (ACO). Our aims were to: describe the demographic and clinical-training characteristics of both institutions' clinical doctoral students; determine their long-term career plans (including how many want a CAC) and factors influencing these plans; examine their views regarding

For peer review only - http://bmjopen.bmj.com/site/about/guidelines.xhtml

CL posts, since these are the first postdoctoral posts on the established IAT pathway; and explore their preparedness for making career decisions.

For peer review only

For peer review only - http://bmjopen.bmj.com/site/about/guidelines.xhtml

METHOD

In reporting our study, we followed the STROBE guidelines.[14]

Design and procedures

We conducted a census survey of all medically qualified PhD students enrolled, at the beginning of autumn 2015, at two universities in England: the University of Oxford (hereafter ‘‘Oxford’’) and UCL. Students who were either suspended or had already submitted their thesis were excluded. MD/DM students were also excluded as their higher degree programme is often less structured and the survey questions may not apply in the same way. Each survey had ethical approval from its home institution and collected its data via its own online questionnaire between the end of October 2015 and the beginning of March 2016. Participants received: an email from senior academics introducing the study; an email invitation which offered an £8 Amazon voucher for participation; two email reminders at Oxford, three at UCL; and one postal reminder inviting them to complete either the online questionnaire or a paper version. The target population was identified from administrative records. Where we were unclear about students' qualifications, after the introductory email we sent an email checking qualifications and excluded those who responded that they were not medically qualified. A total of 523 eligible students were asked to participate (see STROBE diagrams in Supplement Box 1).

Instruments

OUCAGS developed the questionnaire in 2013, as part of its Clinical DPhil Paths longitudinal study (2013-2018) of the career plans of clinical doctoral students. (‘‘DPhil’’ is the term for an Oxford PhD.) Questions were either newly created or adapted from existing questionnaires.[15,16] They covered respondents' characteristics and career plans, including: demographic variables, medical training, predoctoral involvement in research, reason for entering the doctorate, doctoral experience, intention to pursue a CAC and a CL in England (where the NIHR funds a large number of posts), and views regarding CL posts (including how attainable they think these posts are[17]). (See Box 2 in Supplement for details of questionnaire development, the questions reported below, and minor adaptations by UCL (e.g. use of ‘‘PhD’’ instead of ‘‘DPhil’’)).

Analysis

We merged anonymised Oxford and UCL datasets and analysed the data using SPSS 22. We used frequencies to profile respondents (e.g. age, fee status) as well as to compare respondents by gender and intention towards a CAC. For fee status, we grouped UK and EU respondents together as both were eligible for ‘‘home’’ fees and have equal employment rights within the UK. This also

For peer review only - http://bmjopen.bmj.com/site/about/guidelines.xhtml

preserved EU respondents' anonymity in file sharing as their number was small (n= 12 and 9 respondents, from Oxford and UCL, respectively).

We analysed responses to open questions using conventional content analysis[18] to identify themes and counted the number of participants mentioning each recurrent theme. Responses were double-coded by JL and VR in a face-to-face meeting, and any coding inconsistencies were discussed and resolved jointly (Box 3 in Supplement).

As a first step, the data were examined in detail. Then variables with data collected on five- or six-point scales were summarised into three categories (e.g. ''a great deal/a lot'', ''a moderate amount'', ''a little/none at all/not applicable'' – see Results tables), with the full set of response categories referred to where relevant (e.g. for very skewed variables). Using three categories meant that data could be more clearly presented and, in most cases, items from a same question could be entered into Chi-square tests with responses categorised in the same manner. Categories were further collapsed, where necessary, to meet the requirements of Chi-square tests for expected frequencies.[19]

We conducted Chi-square tests of association to determine which of a range of background, doctoral experience, and attitude variables were associated (at the .05 level of statistical significance) with two key career outcomes:

a) intention to pursue a CAC in the long-term (respondents saying CAC *vs.* those with other plans or undecided), and
b) likelihood of seeking a CL in England (extremely or very likely to seek such a post *vs.* not at all to moderately likely).

For statistically significant Chi-square tests, we referred to adjusted standardised residuals to assist with interpreting results. The higher a residual's value, the more a cell contributes to the Chi-square value.

For peer review only - http://bmjopen.bmj.com/site/about/guidelines.xhtml

RESULTS

Characteristics of clinical doctoral students

Response rates

Response rates were 67% and 57% for the Oxford and the UCL surveys, respectively, giving 322 respondents in total. A more resource-intensive approach (e.g. telephone reminders/interviews) may have yielded higher response rates.[20] Only two Overseas (i.e. non-UK/EU) students responded to the UCL questionnaire and, to preserve their anonymity, their data were not shared with Oxford. This paper therefore reports on data from 320 clinical doctoral students, of which 170 (53%) were enrolled at Oxford and 150 (47%) at UCL.

Demographics and training

Of the 320 respondents, 54% (n=174) were male, 44% (n=142) female, and 1% (n=4) did not specify. Analysis for non-response bias by gender showed no bias needed to be accounted for in the analyses. Indeed, respondents were representative of the combined populations of clinical doctoral students of Oxford and UCL as, based on administrative data, 57% (n=299) of students invited to complete the questionnaire were male and 43% (n=224) were female.

Most respondents were aged 30-35 years (Table 1). Figures for UK/EU respondents only (N=279) are comparable to figures for all respondents as 52% (n=146) were male and only 10% (n=28) were under 30 years. However, amongst the Overseas (Oxford) students (N=33), more respondents were male (70%, n=23) and under 30 (49%, n=16) (see Box 4 in Supplement for additional information).

Most participants were in specialty training years 3 to 8 (ST3-8), i.e. had done four or more years of clinical training after medical school and entered a specialty; this included 80 respondents (25% of the total) in years 6 to 8 (ST6-8), who were close to completing training. Approximately one-fifth had completed their training (Table 1).

The majority (84%, n=268) of participants had been involved in medical research prior to their doctorate, but over one-tenth (12%, n=38) had no involvement (see Box 5 in Supplement for additional information). This was similar at Oxford and UCL. The most common type of predoctoral medical research experience was an intercalated degree, that is, a research degree taken during the undergraduate years (48% of respondents, n=154). Also, over one-fifth (21%, n=67) of participants had held a predoctoral fellowship (i.e. an ACF in England, Wales, or Northern Ireland (n=65), or a clinical research fellowship (CRF) in Scotland (n=2)). Additionally, some respondents had: completed an Academic Foundation Programme (19%, n=60), held other posts with time for research (12%,

For peer review only - http://bmjopen.bmj.com/site/about/guidelines.xhtml

n=37), and/or had "other" research-related experiences, such as research towards an MSc thesis or a research assistantship (31%, n=100).

Reason for entering a doctoral programme

Respondents' main reason for undertaking a doctorate centred upon interest in their area of research (46%, n=146) or improving their prospects for an academic/research career (42%, n=134). However, improving their prospects for a clinical career *outside* academia was also mentioned by some (10%, n=33) (see Box 5 in Supplement for additional information). Respondents who had completed their specialist training were particularly likely to cite interest in their area of research (56%, n=32), although over one-third (35%, n=20) specifically wanted to improve their prospects for an academic/research career.

For peer review only

For peer review only - http://bmjopen.bmj.com/site/about/guidelines.xhtml

Table 1 – Respondents' age, training level and long-term career plans: percentages by gender and for all respondents

	All respondentsǂ		
	**Males** (N=174) **%**	**Females** (N=142) **%**	**Total** (N=320) **%**
**Age**			
<30	17	11	14
30-35	56	54	55
36+	14	16	15
No response	13	20	17
**Highest training stage completed or underway***			
Pre-Foundation, in Foundation, or post-Foundation programme	14	10	12
In ST1-2, CT or ACCS, *or* completed ST1-2, CT or ACCS	12	8	10
In ST3-8	56	57	56
ST level not specified	3	4	4
Completed specialist training	16	21	18
**Long-term career plans**			
Clinical academic posts	55	44	50
Clinical service posts with some teaching and research	23	23	23
Clinical service posts with some research	9	9	9
Clinical service posts with some teaching	2	1	2
Clinical service posts *without* teaching or research	1	1	1
Research-only posts	2	3	3
Undecided	8	15	11
Other	1	4	2
No response	-	1	<1

Note: Due to rounding, column percentages may not add to 100.
ǂFour respondents did not provide their gender.
*In the UK, following medical school and a two-year Foundation programme, doctors enter specialist training (ST). The first two years of specialty training are usually referred to as ST1-2, but some specialties have, before specialist training, a core training (CT) or acute care common stem (ACCS) programme. However, this is highly dependent on specialty.

Long-term career planning

Career plans

Half of all respondents expressed a desire to pursue a CAC in the long term, and around a third intended to work in clinical posts with some research, or some teaching and research (Table 1). The figure for respondents wanting a CAC was higher amongst former ACFs/CRFs (63%, n=42). Over one-tenth of respondents, including 13% (n=9) of former ACFs/CRFs and 12% (n=27) of doctors in specialty training, were undecided about their long-term career, although very few wanted purely

For peer review only - http://bmjopen.bmj.com/site/about/guidelines.xhtml

clinical posts or clinical posts with teaching. The proportion of participants undecided or with each type of career plan was similar across doctoral year groups (Year 1 to Year 4 and above).

Chi-square tests showed that greater predoctoral involvement in medical research, and pursuing a doctorate mainly to improve academic/research-career prospects, were each associated with wanting a CAC (Table 2). In contrast, there was no statistically significant association between whether a respondent was planning a CAC and: gender, fee status (UK/EU *vs.* Overseas), age or training stage (Box 6 in Supplement).

For peer review only

Table 2 – Variables associated with wanting a clinical academic career (CAC) *vs.* having other plans or being undecided: statistically significant X^2 tests

Variables	Groupings	Respondents wanting a CAC %	n / N	Adjusted residuals‡	X^2 test df	X^2	p
**Predoctoral involvement in medical research**	**No involvement**	26	10 / 38	-3.0	2	22.193	.0005
	**Some involvement** *(intercalated degree or post(s) with research, possibly with other experience, or "other" experience)*§	46	95 / 205	-1.4			
	**High involvement level** *(intercalated degree and post(s) with research, and possibly "other" experience, too)*§	73	45 / 62	4.1			
**Main reason for doctorate**	Interest in my area of research	49	71 / 146	-.4	2	25.515	.0005
	Improving prospects for an academic/research career	61	82 / 134	3.5			
	Other	15	6 / 39	-4.6			
Influences on long-term career thinking:							
**Wanting intellectual stimulation***	A great deal/A lot	55	157 / 284	5.4	1	29.640	.0005
	A moderate amount/A little/Not at all	6	2 / 34	-5.4			
**Enthusiasm /commitment***	A great deal/ A lot	52	156 / 300	2.9	1	8.480	.004
	A moderate amount/ A little/ Not at all	17	3 / 18	-2.9			
**Self-appraisal of own skills/aptitudes**	A great deal/A lot	56	107 / 191	2.6	2	7.743	.021
	A moderate amount	39	38 / 98	-2.7			
	A little/Not at all	48	14 / 29	-.2			
**Career promotion and prospects**	A great deal/A lot	58	85 / 146	2.7	2	7.300	.026
	A moderate amount	43	51 / 118	-1.9			
	A little/Not at all	43	23 / 54	-1.2			
**Lack of posts in other possible career paths**	A great deal/A lot	27	8 / 30	-2.7	2	14.357	.001
	A moderate amount	36	19 / 53	-2.3			
	A little/Not at all	56	132 / 235	3.7			
CL posts:							
**CL knowledge**	A great deal/A lot	73	27 / 37	3.0	2	19.198	.0005
	A moderate amount	59	69 / 118	2.3			
	A little/Nothing at all	39	63 / 162	-4.1			
**CL attainability (accuracy of statement that CL is attainable)**	Extremely/Very accurate	63	60 / 96	2.9	2	16.079	.0005
	Moderately accurate	54	57 / 105	1.1			
	Slightly/Not at all accurate	36	42 / 117	-3.8			
Sources of information about possible career paths (amount of information received):							
**OUCAGS/ACO**	A great deal/A lot	82	22 / 27	3.4	2	18.917	.0005
	A moderate amount	65	33 / 51	2.2			
	A little/None at all/N/A	44	103 / 236	-4.1			
**Mentor(s)**	A great deal/A lot	64	50 / 78	2.8	2	8.778	.012
	A moderate amount	49	40 / 81	-.2			
	A little/None at all/N/A	44	68 / 156	-2.3			

‡ For explanation and interpretation see Method.
§The following were considered "posts with research": Academic Foundation Programme posts; Academic Clinical Fellow (England, Wales, Northern Ireland); Clinical Research Fellow (Scotland); and responses of "other post with time for research".
*Due to the small numbers selecting "A moderate amount", "A little" and "Not at all", these categories were merged in order to meet X^2 test assumptions (see Method).

For peer review only - http://bmjopen.bmj.com/site/about/guidelines.xhtml

Enthusiasm and commitment to a given career path, as well as wanting intellectual stimulation and to make a difference to patients, were major influencing factors in respondents' long-term career planning (Table 3, and Box 7 in Supplement for further details). In addition, having acceptable working hours and conditions was especially important to respondents who were undecided about their long-term career, with 78% (n=28) of this group saying this had a great deal or a lot of importance.

In Chi-square tests the following were positively associated with wanting a CAC (Table 2): wanting intellectual stimulation, enthusiasm/commitment, self-appraisal of own skills/aptitudes, and career promotion and prospects. Also, a perceived lack of posts in other possible career paths was negatively associated with planning a CAC, possibly as respondents wanting a CAC can always return to a full-time clinical career. There was no statistically significant association between planning a CAC and other long-term career planning factors examined (Box 6 in Supplement).

Table 3 – Factors influencing respondents' thinking about their long-term career: percentage saying ''a great deal'' or ''a lot'', by whether respondents want a clinical academic career (CAC) *vs.* having other plans or being undecided, and for all respondents

	**Respondents wanting a CAC‡** (N=159) **%**	**Respondents *not* wanting a CAC‡** (N=160) **%**	**Total** (N=320) **%**
Enthusiasm/commitment<br>*(A great deal)**	98<br>*(67)*	90<br>*(52)*	94<br>*(60)*
Wanting intellectual stimulation<br>*(A great deal)**	99<br>*(63)*	79<br>*(38)*	89<br>*(50)*
Wanting to make a difference to patients<br>*(A great deal)**	88<br>*(51)*	79<br>*(41)*	84<br>*(46)*
Self-appraisal of own skills/aptitudes	67	53	60
Wanting a career that fits my domestic circumstances	56	54	55
Wanting a career with acceptable working hours/conditions	50	59	54
Exposure to role models	54	44	49
Career promotion and prospects	54	38	46
Future financial prospects	39	38	38
Advice from others	25	19	22
Lack of posts in other possible career paths	5	14	9

‡Base: All participants (N=319) who responded to the question about their career plans.
*Some variables were considerably skewed. Where the mode for the whole group of respondents (total) was ''a great deal'', figures for the modal response-only are provided in brackets.

For peer review only - http://bmjopen.bmj.com/site/about/guidelines.xhtml

Drawbacks of a clinical academic career

Respondents not intending to work in either clinical academia or research-only posts (N=153) were asked how much each of a range of factors might be a reason for excluding a CAC from their long-term career plans. This group included over a third (37%, n=25) of former ACFs/CRFs. Nearly two-thirds of the group (66%, n=101) said that the difficulty of obtaining research grants had a great deal or a lot of weight in their planning. Other factors with considerable weight were (% saying ‘‘a great deal’’ or ‘‘a lot’’):

- the small number of senior academic appointments available (50%, n=76)
- the competing pressures from service, teaching and research (47%, n=72)
- the limited future financial prospects compared to alternative careers (34%, n=52)
- needing work arrangements that are compatible with their caring responsibilities (30%, n=46, including 45%, n=34, of women but only 15%, n=11, of men), and
- family circumstances making them unwilling to move for work (27%, n=42).

Responses to the open question “what might make a clinical academic career more attractive to you?”, asked of all participants, confirmed that issues around obtaining research grants, securing posts, and career progression are of particular concern when considering a CAC, as responses centred around these themes (Box 3 in Supplement).

Clinical lectureships

Over three-fifths of participants (Table 4), including 65% (n=181) of UK/EU respondents, were at least moderately likely to seek a CL post in England at an appropriate point after completing their doctoral studies. This figure rose to 78% (n=52) for former ACFs/CRFs, and to 98% (n=41) amongst former ACFs/CRFs wanting a CAC. In addition, nearly two-thirds of all participants, and over four-fifths of former ACFs/CRFs (82%, n=55), perceived CL posts to be at least moderately attainable for them (Table 4). Although overall women were more cautious about the attainability of CL posts, amongst respondents wanting a CAC similar proportions of men (74%, n=71) and women (73%, n=46) felt they were at least moderately attainable.

Respondents in speciality training, and those aged 30-35, were the most likely to seek a CL post in England. This is supported by Chi-square tests (Table 5), which also revealed positive associations between likelihood of seeking a CL in England and: wanting a CAC; being from the UK/EU; predoctoral involvement in medical research; knowledge of CLs; and feeling that a CL is attainable. However, there was no statistically significant association between likelihood of seeking a CL post

For peer review only - http://bmjopen.bmj.com/site/about/guidelines.xhtml

and gender (Box 8 in Supplement.) Chi-square tests also showed a positive association between wanting a CAC and perceiving that CL posts are attainable (Table 2).

Table 4 – Respondents' likelihood of seeking a CL, and views on CL attainability and own knowledge about CLs: percentages by gender and for all respondents

	All respondents‡		
	Males %	Females %	Total %
**Likelihood of seeking a CL***	*N=173**	*N=141**	*N=318**
Extremely/Very likely	45	40	42
Moderately likely	17	23	20
Slightly/Not at all likely	36	36	36
No response	1	2	2
**CL attainability** *Accuracy of statement that respondent could become a CL in England, if wanted*	*N=174*	*N=142*	*N=320*
Extremely/Very accurate	37	22	30
Moderately accurate	29	36	33
Slightly/Not at all accurate	33	42	37
No response	1	-	<1
**Knowledge of CL** *Amount known*	*N=174*	*N=142*	*N=320*
A great deal/A lot	12	11	12
A moderate amount	40	34	37
A little/Nothing at all	48	54	51
No response	-	1	1

Note: Due to rounding, column percentages may not add to 100.
‡Four respondents did not provide their gender.
*The question about likelihood of seeking a CL was not asked of two participants who stated that they intended to work in clinical posts without teaching or research.

Although many respondents thought that they may successfully apply for a CL, only a minority (Table 4) felt that they knew a great deal or a lot about CL posts in England. In fact, just over half, including one-quarter (25%, n=17) of former ACFs/CRFs, felt that they had little or no knowledge (Table 4). Also, although Chi-square tests showed a positive association between wanting a CAC and knowledge of CL posts (Table 2), two-fifths (40%, n=63) of those planning a CAC nevertheless had little or knowledge of these CL posts.

For peer review only - http://bmjopen.bmj.com/site/about/guidelines.xhtml

Table 5 – Variables associated with likelihood of seeking a CL: statistically significant X^2 tests

		Respondents extremely/very likely to seek a CL			X^2 test		
**Variables**	**Groupings**	**%**	**n / N**	**Adjusted residuals‡**	**df**	**X^2**	**p**
**Fee status**	UK/EU	47	128 / 273	3.4	1	11.383	.001
	Non-EU (Overseas)	16	5 / 32	-3.4			
**Age**	<30 years	27	12 / 44	-2.5	2	11.534	.003
	30-35 years	51	89 / 173	3.4			
	36 years and over	32	14 / 44	-1.8			
**Training stage**	Pre-specialty	22	8 / 36	-2.7	2	12.149	.002
	In specialty	49	108 / 221	3.4			
	Completed	32	18 / 56	-1.8			
**Predoctoral involvement in medical research**	**No involvement**	21	8 / 38	-2.9	2	24.502	.0005
	**Some involvement** *(intercalated degree or post(s) with research, possibly with other experience, or "other" experience)* §	39	77 / 199	-1.9			
	**High involvement level** *(intercalated degree and post(s) with research, and possibly "other" experience, too)* §	68	42 / 62	4.5			
**Long-term career plans**	CAC	67	105 / 158	8.5	3	72.557	.0005
	Clinical service with research (possibly also teaching)	18	18 / 99	-6.0			
	Undecided	23	8 / 35	-2.5			
	Other plans	15	3 / 20	-2.6			
**CL knowledge**	A great deal/ A lot	65	24 / 37	2.9	2	31.140	.0005
	A moderate amount	57	66 / 116	3.8			
	A little/Nothing at all	28	44 / 158	-5.5			
**CL attainability (accuracy of statement that CL is attainable)**	Extremely/Very accurate	70	66 / 95	6.3	2	52.138	.0005
	Moderately accurate	44	46 / 105	.2			
	Slightly/Not at all accurate	20	22 / 112	-6.2			
Sources of information about possible career paths (amount of information received):							
**Academic/ research supervisor(s)**	A great deal/A lot	48	49 / 103	1.2	2	6.443	.040
	A moderate amount	48	52 / 108	1.3			
	A little/None at all/N/A	33	33 / 101	-2.5			
**Senior academics**	A great deal/A lot	50	31 / 62	1.3	2	9.356	.009
	A moderate amount	53	48 / 91	2.2			
	A little/None at all/N/A	35	55 / 159	-3.0			
**OUCAGS/ACO**	A great deal/A lot	59	16 / 27	1.8	2	17.804	.0005
	A moderate amount	65	33 / 51	3.5			
	A little/None at all/N/A	36	82 / 230	-4.2			
**Mentor(s)**	A great deal/A lot	57	43 / 76	2.7	2	10.053	.007
	A moderate amount	46	37 / 81	.6			
	A little/None at all/N/A	35	53 / 152	-2.9			

‡ For explanation and interpretation see Method.
§The following were considered "posts with research": Academic Foundation Programme posts; Academic Clinical Fellow (England, Wales, Northern Ireland); Clinical Research Fellow (Scotland); and responses of "other post with time for research".

For peer review only - http://bmjopen.bmj.com/site/about/guidelines.xhtml

Career preparedness

Most respondents (64%, n=204) felt that their doctoral programme was preparing them extremely or very well for a possible future CAC, although this figure was higher for men (72%, n=125) than for women (55%, n=78).

A proportion (23%, n=75) of respondents had little or no encouragement from their PhD supervisor(s) to reflect on their career development needs, but most (56%, n=180) had a formal or informal mentor they met regularly. However, only 50% (n=71) of women had such a mentor, compared to 62% (n=107) of men. Also, over a third (34%, n=109) of all respondents stated that they did not have a mentor but would like to find one, and this figure increased to 41% (n=58) amongst women (*vs.* 29%, n=50, amongst men).

Respondents who wanted a CAC were more likely to say that they get a great deal or a lot of information about possible careers from mentors (31%, n=50 *vs.* 18%, n=28 for those who had other plans or were undecided). In contrast, over three-fifths of women *not* intending to pursue a CAC received little or no such information from senior academics (64%, n=50 *vs.* 50%, n=39, of men) and mentors (63%, n=49 *vs.* 45%, n=35, of men). (Box 9 in Supplement contains additional data.)

Men and women obtained similar amounts of information from OUCAGS/UCL's ACO, with 28% (n=48) of men and 22% (n=31) of women receiving at least moderate amounts of information from this source. Moreover, receiving information about careers from OUCAGS/ACO and mentor(s) each had a positive relationship with both wanting a CAC and likelihood of seeking a CL (Tables 2 and 5). The latter also was positively associated with receiving information about careers from academic/research supervisor(s) and senior academics. Interestingly, there were no statistically significant relationships between either wanting a CAC or likelihood of seeking a CL and: having a mentor, encouragement from supervisors to reflect on career needs, or information from peers and newsletters (Boxes 6 and 9 in Supplement).

For peer review only - http://bmjopen.bmj.com/site/about/guidelines.xhtml

DISCUSSION

The clinical PhD students in our study are aged mainly between 30-35 years, in specialty training (with a quarter close to completion), and gender balanced once Overseas students are accounted for. Encouragingly, half of our clinical doctoral students want to pursue a CAC, with women as likely as men to express this career goal. Amongst those who have already completed their clinical specialist training (nearly one-fifth of our clinical doctoral students) there is also interest in CACs. Indeed, training level does not seem to affect long-term plans, and neither does age. Greater predoctoral involvement in medical research is strongly associated with wanting a CAC but, unexpectedly, less than two-thirds of former ACFs/CRFs, i.e. doctoral students already on the clinical academic path, are planning to pursue a CAC. Given that our cohort, who secured doctorates at Oxford/UCL, might be regarded as ‘‘high-achievers’’, it is surprising that a higher proportion did not want a CAC. On a more positive note, many doctoral students, even if not planning a CAC, *are* interested in a career that combines clinical work and research, with very few wanting purely clinical posts. In common with others,[9-11] we found that uncertainties around funding and career progression influence career plans, and are perceived as drawbacks of CACs. Respondents not planning a CAC, including former ACFs/CRFs, are particularly worried about the small number of senior academic appointments available, as well as the difficulty of obtaining research grants and work-life balance. Whilst the desire for a career that fits domestic circumstances is not statistically associated with career plans, these issues are given more weight by women than men. Such factors may go on to influence women’s actual postdoctoral career choices,[21] as women may evaluate barriers encountered differently to men, leading to a change in their career intentions.[22]

We were curious about the place of CL posts in career plans. These posts might be considered a logical step in a postdoctoral CAC but a recent report[9] suggests their take-up is lower than expected. In light of this, our data are encouraging as around three-fifths of respondents were at least moderately likely to apply for a CL post – a greater proportion than were planning a CAC. This might indicate that some view CL posts as valuable for career progression, whether as part of a CAC or not. Concomitantly, however, progression to a CL may be affected by the low levels of knowledge about the posts. Additionally, we found some issues with career preparedness (also reported by others[9]). These were more prominent amongst women, who were more likely not to have a mentor but want one and, if not wanting a CAC, received less guidance from senior academics. Exposure to role models, often suggested as important[23,24] in career development, did not appear to be significant amongst our respondents, but the positive association between receiving information from OUCAGS/ACO and wanting a CAC is reassuring as it supports the benefit of such infrastructure.

For peer review only - http://bmjopen.bmj.com/site/about/guidelines.xhtml

Strengths and limitations of the study

Retention of postdoctoral clinical academics is crucial to the progress of academic medicine. However, the relevant literature is predominantly North American[8] and also tends to rely on retrospective data.[9] Ours is the first UK study to describe, based on data from two universities, the career aspirations of medically qualified PhD students and what influences them as they make their career plans. As it is a cross-sectional study, causality cannot be inferred from the associations we found between career plans and other variables. It is also not possible to say whether respondents' intentions will be implemented as planned and our findings may not generalise to other institutions as our data are from two research-intensive universities in England.

Implications of findings

Our study, which adds the perspective of UK clinical doctoral students to the existing literature, suggests four areas of action that will support the development of a stable pipeline of clinical academics. Firstly, we suggest promoting increased numbers of flexible opportunities for doctors to gain medical research experience, starting from their undergraduate studies. Indeed, we found that those with the most research experience were more likely to want a CAC and, although research experience may not directly influence that choice, it may contribute to an informed career decision. Secondly, institutions should offer comprehensive career support for their clinical doctoral students. This could be via delivery of a targeted careers package by a local institutional unit similar to OUCAGS/ACO which could: address knowledge about CL posts and other postdoctoral opportunities; facilitate mentors and doctoral students to develop individualised career plans; meet the unfulfilled need for mentors; and brief senior academics on current CAC opportunities to enable them to provide up-to-date guidance. Thirdly, we suggest a need to increase the flexibility of CAC paths even further than at present. CL posts are the first postdoctoral position to afford clinicians the opportunity to develop as independent research leaders, and without this opportunity progressing further academically may be difficult. We have identified that family mobility is an issue for our clinical doctoral students, but identifying a CL post in the right specialty, in the right place and at the right time (e.g. as the doctorate comes to an end) is challenging, and sometimes impossible. This is exacerbated by eligibility criteria, particularly for NIHR CL posts, requiring that applicants have at least one year of training remaining (the post ceases at completion of training), since, as we found, many clinical doctoral students have completed, or nearly completed, their training. We therefore suggest that criteria for CL posts be relaxed. Various postdoctoral points of entry into CL posts could be allowed (including after training completion) and the posts should not be specialty-specific so as to afford enhanced opportunities to all postdoctoral clinicians. Increased flexibility might also attract back postdoctoral clinicians who had originally not pursued a CAC, such as when family

For peer review only - http://bmjopen.bmj.com/site/about/guidelines.xhtml

circumstances change and work-life balance may seem more achievable. Such changes could help medical schools overcome the recruitment challenges that have been reported.[7] Finally, more senior posts are required since their lack seems to significantly influence career decisions. Universities are unlikely to be able to create sufficient fully funded senior posts, so formalising research time in a subset of NHS consultant posts, and establishing them as joint university appointments, could cost-effectively expand the definition of a CAC. Ensuring the visibility of these posts would demonstrate a clear career trajectory to encourage postdoctoral retention. Although current NHS service and funding pressures might make this seem low priority, we would argue that such joint appointments are essential as research activity is centrally positioned in the NHS constitution[25] and, crucially, it improves patient care and service quality.[26-27]

Conclusions and further research

Our findings suggest that many doctoral students with the potential to be the clinical academics of the future may never tread this path, some (former ACFs/CRFs) may choose to abandon it, and some may be excluded by inflexible eligibility criteria for postdoctoral posts. It is therefore important for those promoting CACs to provide sufficient, flexible opportunities to engage in clinical academia to all postdoctoral clinicians. This should include tackling issues around the career pathway beyond the clinical training period and building career preparedness and resilience. Our findings also suggest that an additional approach to increasing the numbers of clinical academics might be to ensure predoctoral doctors have ample opportunities to gain research experience. Further research should investigate whether clinical doctoral students' intentions will be implemented as planned (e.g. will equal numbers of men and women actually develop CACs?) and seek to identify which factors contribute to actually pursing a CAC. The Clinical DPhil Paths longitudinal study is currently collecting data on this in the UK.

For peer review only

For peer review only - http://bmjopen.bmj.com/site/about/guidelines.xhtml

FOOTNOTES

Acknowledgements: We thank the clinical doctoral students who took part in our study and Abigail Hipkin (Research Education and Training Administrator, OUCAGS), who assisted with data collection at Oxford. We are also grateful to the University of Oxford and UCL administrative services and the UCL graduate tutors who helped us identify the students to invite to participate in our study.

Contributors: JL, DB and CP developed the Clinical DPhil Paths questionnaire. JL collected the Oxford data and VR collected the UCL data. All authors conceived the data analysis plan and made substantive contributions to the interpretation of findings. JL conducted the quantitative data analysis and reporting, with assistance from VR. JL and VR undertook the qualitative data analysis. JL and DB wrote the first draft. All authors contributed to further drafts and all approved the final version. All authors had full access to all of the data (including statistical reports and tables) in the study and can take responsibility for the integrity of the data and the accuracy of the data analysis. The study guarantors are DB (for Oxford) and GR (for UCL).

Funding: Oxford – This research was funded by OUCAGS, which is part of the University of Oxford Medical School. UCL – This research was funded by the National Institute for Health Research University College London Hospitals Biomedical Research Centre. NJF and HB were (in part) supported by the National Institute for Health Research (NIHR) Collaboration for Leadership in Applied Health Research and Care (CLAHRC) North Thames at Bart's Health NHS Trust. The views expressed are those of the author(s) and not necessarily those of the NHS, the NIHR or the Department of Health. The authors conducted the study with no involvement from the funders.

Competing interests: All authors have completed the ICMJE uniform disclosure form at http://www.icmje.org/coi_disclosure.pdf and declare: JL, CP and DB declare that JL and DB are employed by, and CP directs, OUCAGS, which exists to promote and advance clinical academic careers; CP declares that his salary is in part paid for by Health Education England in the Thames Valley; VR and GR declare that the former works for, and the latter manages, UCL's Academic Careers Office, which is funded by UCLH's NIHR Biomedical Research Centre, and thus the National Institute for Health Research; no other financial relationships with any organisations that might have an interest in the submitted work in the previous three years besides the support provided for the submitted work by the organisations described above under ''funding''; no other relationships or activities that could appear to have influenced the submitted work.

Ethical approval: Oxford survey approved by the University of Oxford's Medical Sciences Inter-Divisional Research Ethics Committee (reference MSD-IDREC-C1-2014-149). UCL survey approved by

For peer review only - http://bmjopen.bmj.com/site/about/guidelines.xhtml

the UCL Research Ethics Committee (application 7335/002). All participants gave informed consent to take part.

Data sharing: No additional data available.

Transparency: The study guarantors affirm that this manuscript is an honest, accurate, and transparent account of the study being reported; that no important aspects of the study have been omitted; and that any discrepancies from the study as planned have been explained.

Licence: The Corresponding Author has the right to grant on behalf of all authors and does grant on behalf of all authors, a worldwide licence (http://www.bmj.com/sites/default/files/BMJ%20Author%20Licence%20March%202013.doc) to the Publishers and its licensees in perpetuity, in all forms, formats and media (whether known now or created in the future), to i) publish, reproduce, distribute, display and store the Contribution, ii) translate the Contribution into other languages, create adaptations, reprints, include within collections and create summaries, extracts and/or, abstracts of the Contribution and convert or allow conversion into any format including without limitation audio, iii) create any other derivative work(s) based in whole or part on the on the Contribution, iv) to exploit all subsidiary rights that currently exist or as may exist in the future in the Contribution, v) the inclusion of electronic links from the Contribution to third party material where-ever it may be located; and, vi) licence any third party to do any or all of the above. All research articles will be made available on an Open Access basis (with authors being asked to pay an open access fee—see http://www.bmj.com/about-bmj/resources-authors/forms-policies-and-checklists/copyright-open-access-and-permission-reuse). The terms of such Open Access shall be governed by a Creative Commons licence—details as to which Creative Commons licence will apply to the research article are set out in our worldwide licence referred to above.

For peer review only

For peer review only - http://bmjopen.bmj.com/site/about/guidelines.xhtml

REFERENCES

1 Academy of Medical Sciences. *The tenure-track clinical scientist: a new career pathway to promote recruitment into clinical academic medicine*. London: AMS, 2000.

2 Academy of Medical Sciences. *Clinical academic medicine in jeopardy: recommendations for change.* London: AMS, 2002 https://www.acmedsci.ac.uk/viewFile/publicationDownloads/pclinaca.pdf (accessed 7 Dec 2016).

3 Milewicz DM, Lorenz RG, Dermody TS, *et al.* Rescuing the physician-scientist workforce: the time for action is now. *J Clin Invest* 2015;125(10):3742-47. doi:10.1172/JCI84170

4 Windsor J, Searle J, Hanney R, *et al.* Building a sustainable clinical academic workforce to meet the future healthcare needs of Australia and New Zealand: report from the first summit meeting. *Intern Med J* 2015;45(9):e965:e971. doi:10.1111/imj.12854

5 Modernising Medical Careers. *Medically- and dentally-qualified academic staff: recommendations for training the researchers and educators of the future. Report of the Academic Careers Sub-Committee of Modernising Medical Careers and the UK Clinical Research Collaboration*. London: UK CRC, 2005 http://www.ukcrc.org/wp-content/uploads/2014/03/Medically_and_Dentally-qualified_Academic_Staff_Report.pdf (accessed 7 Dec 2016).

6 Funston G, Cera C, Kirkham D, *et al.* The road to a clinical academic career. *BMJ Careers 2015*. http://careers.bmj.com/careers/advice/The_road_to_a_clinical_academic_career

7 Medical Schools Council. *A survey of staffing levels of medical clinical academics in UK medical schools, as at 31 July 2015*. London: Medical Schools Council, 2016 http://www.medschools.ac.uk/SiteCollectionDocuments/MSC-survey-2016.pdf (accessed 9 Mar 2017).

8 Edmunds LD, Ovseiko PV, Shepperd S, *et al.* Why do women choose or reject careers in academic medicine? A narrative review of empirical evidence. *Lancet* 2016;Apr19. doi:10.1016/S0140-6736(15)01091-0 (accessed 28 Apr 2016).

9 Medical Research Council, Academy of Medical Sciences, British Heart Foundation, et al. *A cross-funder review of early-career clinical academics: enablers and barriers to progression*. London: MRC, 2015 https://www.mrc.ac.uk/documents/pdf/review-of-early-career-clinical-academics/ (accessed 26 Jan 2017).

10 Ranieri V, Barratt H, Fulop N, *et al.* Factors that influence career progression among postdoctoral clinical academics: a scoping review of the literature. *BMJ Open* 2016;6:e013523. doi:10.1136/bmjopen-2016-013523

For peer review only - http://bmjopen.bmj.com/site/about/guidelines.xhtml

11 Ranieri V, Barratt H, Fulop N, *et al.* Clinical academics' postdoctoral career development. *BMJ* 2015;351:h6945. doi:10.1136/bmj.h6945 (Published 30 December 2015).

12 Lyons OTA, Smith C, Winston JS, *et al.* Impact of UK academic foundation programmes on aspirations to pursue a career in academia. *Med Educ* 2010;44(10):996-1005. doi: 10.1111/j.1365-2923.2010.03787.x

13 Best D, Lopes J, Pugh C. It's the duty of every doctor to get involved with research. *BMJ Online* [eLetter] 27 November 2015. doi:10.1136/bmj.h6329 http://www.bmj.com/content/351/bmj.h6329/rapid-responses (accessed 9 Mar 2017).

14 von Elm E, Altman DG, Egger M, *et al.* The strengthening the reporting of observational studies in epidemiology (STROBE) statement: guidelines for reporting observational studies. *BMJ* 2007;335(7624):806-808. doi: 10.1136/bmj.39335.541782.AD http://www.bmj.com/content/335/7624/806 (accessed 2 May 2017).

15 Hodsdon L, Buckley A. *Postgraduate research experience survey: 2011 results.* Higher Education Academy 2011 www.heacademy.ac.uk/resources/detail/postgraduate/PRES_2011_report (accessed 6 Jul 2016).

16 Goldacre MJ, Lambert T, Goldacre R, *et al.* Career plans and views of trainees in the academic clinical fellowship programme in England. *Med Teach* 2011;33:11:e637-e643. doi: 10.3109/0142159X.2011.610842

17 Ajzen I. The theory of planned behaviour. *Organ Behav Hum Decis Process* 1991;50(2):e179-211.

18 Hsieh H-F, Shannon SE. Three approaches to qualitative content analysis. *Qual Health Res* 2005;18(9):e1277-1288.

19 Bland M. *An introduction to medical statistics*. Oxford: Oxford University Press 2015:193–197.

20 de Leeuw, ED. To mix or not to mix data collection modes in surveys. *J Off Stat* 2005;21(2)e233–255.

21 Levine RB, Lin F, Kern DE, *et al.* Stories from early-career women physicians who have left academic medicine: a qualitative study at a single institution. *Acad Med* 2011;86:6:e752-e758.

22 Ng THW, Sorensen KL, Eby LT, *et al.* Determinants of job mobility: a theoretical integration and extension. *J Occup Organ Psychol* 2007;80: e363-e386. doi:10.1348/096317906X130582

23 Levinson W, Kaufman K, Clark B, *et al.* Mentors and role models for women in academic medicine. *West J Med* 1991;154:423-426.

For peer review only - http://bmjopen.bmj.com/site/about/guidelines.xhtml

24 Borges NJ, Navarro AM, Grover A, *et al.* How, when, and why do physicians choose careers in academic medicine? A literature review. *Acad Med* 2010;85(4):680-6. doi: 10.1097/ACM.0b013e3181d29cb9

25 Department of Health. *NHS constitution for England*. 27 July 2015. www.gov.uk/government/publications/the-nhs-constitution-for-england (accessed 2 Nov 2016).

26 Ozdemir BA, Karthikesalingam A, Sinha S, *et al.* Research activity and the association with mortality. *PLoS ONE* 2015;10(2):e0118253. doi:10.1371/journal.pone.0118253

27 Boaz A, Hanney S, Jones T, *et al.* Does the engagement of clinicians and organisations in research improve healthcare performance: a three-stage review. *BMJ Open* 2015;5:e009415. doi:10.1136/bmjopen-2015-009415

For peer review only

The clinical academic workforce of the future: a cross-sectional study of factors influencing career decision-making amongst clinical PhD students at two research-intensive UK universities

SUPPLEMENTARY INFORMATION

For peer review only

Box 1 – STROBE diagrams: a) Oxford census survey; b) UCL census survey

Box 2 – Questionnaire development and questions used

Box 3 – Analysis of open questions

Box 4 – Additional information about respondents: a) Gender and fee status of clinical doctoral students: percentages by university and for all respondents; b) Specialty of respondents: percentages by university and for all respondents

Box 5 – Respondents' predoctoral involvement in medical research and reason for the doctorate: percentages by gender and for all respondents

Box 6 – Variables not associated with wanting a clinical academic career (CAC) *vs.* having other plans or being undecided: statistically *non*-significant X^2 tests

Box 7 – Factors influencing respondents' thinking about their long-term career: percentages for those who want a clinical academic career (CAC) and those who do not/are undecided, and for all respondents

Box 8 – Variables not associated with likelihood of seeking a CL: statistically *non*-significant X^2 tests

Box 9 – Sources of information about possible career paths: by gender for those who want a clinical academic career (CAC) and those who have other plans/are undecided, and for all respondents

For peer review only - http://bmjopen.bmj.com/site/about/guidelines.xhtml

Box 1 – STROBE diagrams

A. Oxford census survey

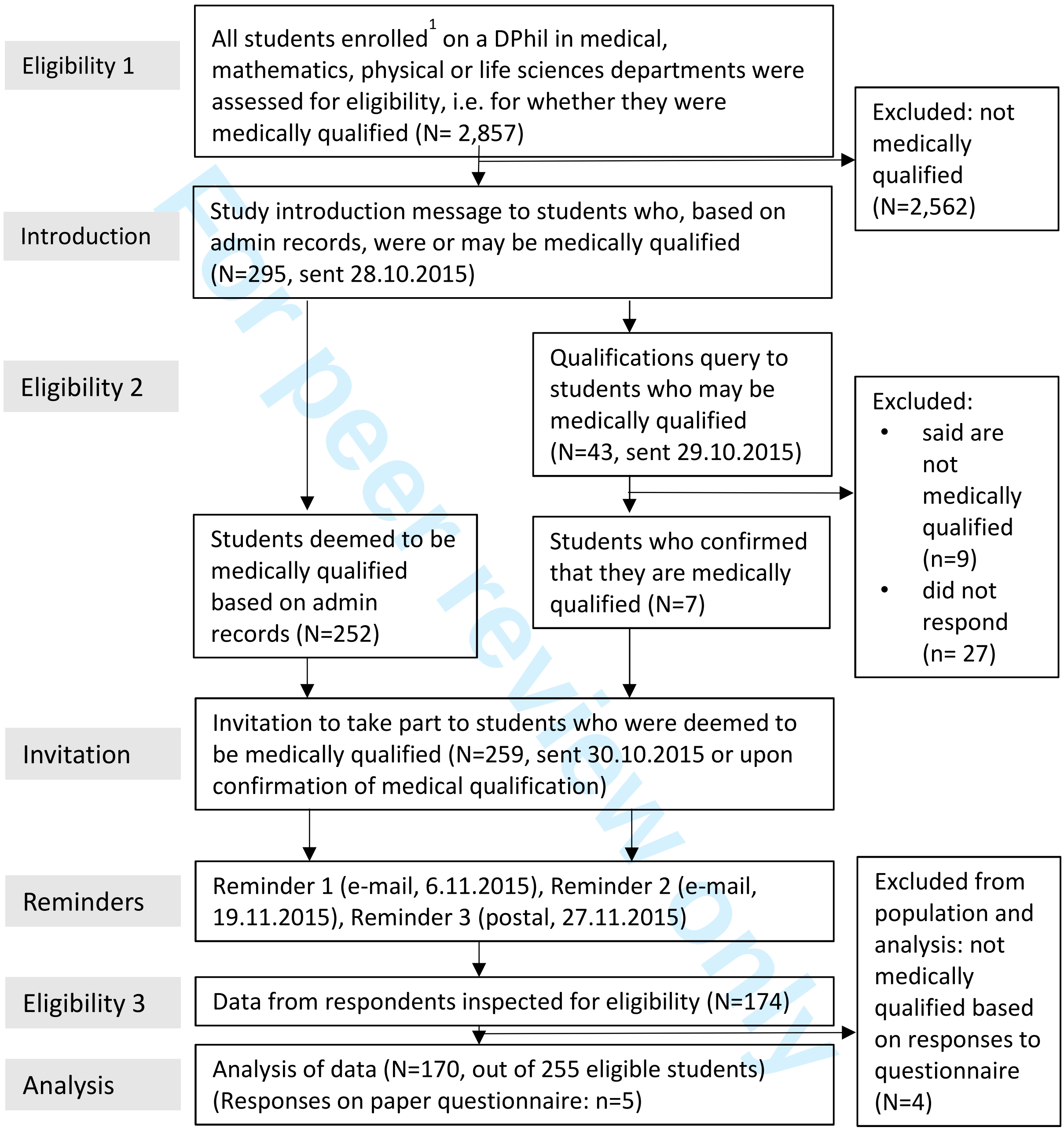

For peer review only

Box 1 (cont.)

B. UCL census survey

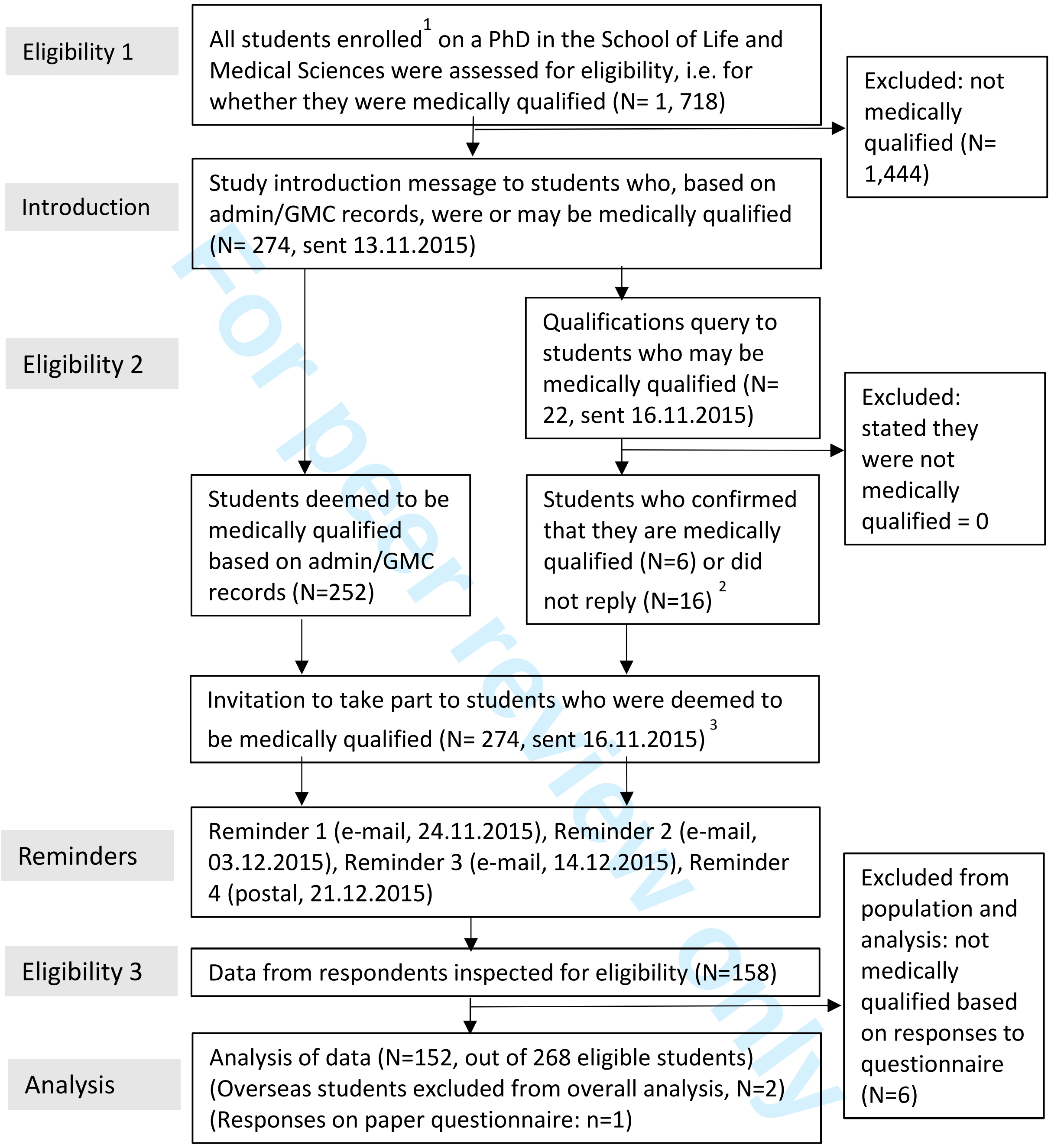

Notes

[1] All DPhil students who were enrolled on a full-time or part-time programme were assessed for eligibility (although at Oxford there were no part-time students). Suspended students were excluded as well as those who had already submitted their thesis.

[2] Both those who confirmed that they were medically qualified and those who did not reply were retained in this census survey.

[3] Due to administrative constraints, a number of students (N=33) were invited on 18.01.16 and data collection finished on 3.03.16.

For peer review only - http://bmjopen.bmj.com/site/about/guidelines.xhtml

Box 2 – Questionnaire development and questions used

OUCAGS developed the questionnaire, as part of its Clinical DPhil Paths longitudinal study (2013-2018) of the career plans of clinical doctoral students, as follows:

1) JL developed some of the questions below and adapted others from existing questionnaires.
2) The face validity of the questions was then checked by DB and CP, followed by question refinement by JL, DB and CP.
3) Further face validation was performed in a pilot exercise with three volunteer clinical lecturers who had completed PhDs. (Two volunteers completed the questionnaire online and one on paper.)
4) Questions were revised to address any wording issues which pilot volunteers raised.

Ahead of the 2015 data collection, and following analysis of the Oxford 2014 focus-group data, the question about knowledge of clinical lectureships was added. Also, the wording of the questions about qualifications was reviewed in discussion with UCL and small adjustments were made. For its survey, UCL also made minor adjustments to the wording of questions, such as using the term ''PhD'' instead of ''DPhil''.

0. Qualifications

0.A. What academic qualifications do you hold? (*Tick all that apply)*

1. BSc
2. BMedSci
3. BA
4. MB ChB (or equivalent)
5. MD (taught)
6. MD by research
7. MSc (taught)
8. MSc by research
9. MRes
10. MPhil
11. Other (please specify):

0.B. What fields are your qualifications in? (*Tick all that apply)*

1. Medicine
2. Dentistry
3. Biomedical field
4. Non-medical life, mathematical or physical science (e.g. zoology, chemistry, computer science)
5. Any other field(s) (e.g. humanities, social sciences)

Note: These questions were used to establish whether respondents were medically qualified.

For peer review only

Box 2 (cont.)

1. Medical training

1.A. Which level of training are you currently in? (*Tick one*)

1. Foundation programme (or equivalent) **[→ SKIP Questions 1.B. & 1.C.]**
2. Core training (or equivalent) **[→ SKIP Questions 1.B. & 1.C.]**
3. Acute Care Common Stem (or equivalent) **[→ SKIP Questions 1.B. & 1.C.]**
4. Specialty training (or equivalent) **[→ Go to Question 1.C.]**
5. NOT APPLICABLE: I am between levels of training **[→ Go to Question 1.B.]**
6. NOT APPLICABLE: I have left training and do not plan to return **[→ Go to Question 1.B.]**
7. NOT APPLICABLE: I have fully completed my training **[→ SKIP Questions 1.B. & 1.C.]**

1.B. What is the highest stage of clinical training which you have FULLY COMPLETED so far? (*Tick one*)

1. Medical School **[→ SKIP Question 1.C.]**
2. Foundation Programme (or equivalent) **[→ SKIP Question 1.C.]**
3. Core Training (or equivalent) **[→ SKIP Question 1.C.]**
4. Acute Care Common Stem (or equivalent) **[→ SKIP Question 1.C.]**
5. Specialty Training (or equivalent) **[→ SKIP Question 1.C.]**

1.C. Please specify your Specialty Training level. (*Enter ST or SpR, followed by a number 1 to 10*):
[text box]

1.D. What is, or will be, your medical specialty? *If you have not yet decided, please enter 'not applicable'.*
[text box]

For peer review only

Box 2 (cont.)

2. Prior involvement in research

2.A. What involvement in medical research did you have prior to your [DPhil/PhD]? (*Tick all that apply*)

1. No involvement
2. Intercalated degree
3. Research rotation as an Academic Foundation Programme doctor
4. Research block or regular release as an Academic Clinical Fellow (England, Wales, Northern Ireland)
5. Research block or regular release as a Clinical Research Fellow (Scotland)
6. Other post with time for research
7. Other (please specify)

3. DPhil / PhD

3.A. What was your MAIN reason for choosing to do a [DPhil/PhD]? (*Tick one*)

1. Interest in my area of research
2. Improving my prospects for an academic/research career
3. Improving my prospects for a clinical career outside academia
4. Other reason (please specify)

Note: This question is adapted from Hodsdon and Buckley (2011) – see References.

3.B. Overall, how much encouragement do you receive from your [DPhil/PhD] supervisor(s) to reflect on your career development needs? (*Tick one*)
Response options: A great deal; A lot; A moderate amount; A little; None at all

For peer review only

Box 2 (cont.)

4. Career paths

4.A. In your long-term career, in what type of posts do you mainly intend to work? (*Tick one*)

1. Clinical academic posts **[→ Skip Question 4.B.]**
2. Clinical service posts with some teaching and research
3. Clinical service posts with some research
4. Clinical service posts with some teaching
5. Clinical service posts without teaching or research
6. Research-only posts **[→ Skip Question 4.B.]**
7. Undecided
8. Other (please specify)

Note: This question is adapted from Goldacre *et al.* (2011) – see References.

4.B. How much would each of the following be a reason for excluding a clinical academic career from your long-term career plans? (*Tick one per row*)
Response options: A great deal; A lot; A moderate amount; A little; Not at all; Not applicable to me

1. The difficulty of obtaining research grants
2. The small number of senior academic appointments available
3. The competing pressures from service, teaching, and research
4. The limited future financial prospects compared to alternative careers
5. My perception of less favourable conditions of employment compared with NHS colleagues (in respects other than salary)
6. The possibility of a significantly longer training period to completion of training compared with NHS colleagues
7. Lack of exit paths if pursuing an academic career path and then changing my mind
8. Needing work arrangements that enable me to meet caring responsibilities (for children and/or adults)
9. Family circumstances making me unwilling to move for work
10. Realising that research is not for me
11. Other reasons (please specify)

Note: This question is adapted from Goldacre *et al.* (2011) – see References.

For peer review only - http://bmjopen.bmj.com/site/about/guidelines.xhtml

Box 2 (cont.)

4.C. How much does each of the following factors influence your thinking about your long-term career? (*Tick one per row*)
Response options: A great deal; A lot; A moderate amount; A little; Not at all

1. Wanting a career that fits my domestic circumstances
2. Future financial prospects
3. Wanting a career with acceptable working hours/working conditions
4. Advice from others
5. Wanting intellectual stimulation
6. Wanting to make a difference to patients
7. Lack of posts in other possible career paths
8. Wanting to do what I really want to do (enthusiasm/commitment)
9. Self-appraisal of own skills/aptitudes
10. Career promotion and prospects
11. Exposure to role models
12. Other (please give details)

Note: This question is adapted from Goldacre *et al.* (2011) – see References.

4.D. What might make a clinical academic career more attractive to you?
[text box]

4.E. How well do you feel your [DPhil/PhD] programme will/would prepare you for a clinical academic career, if this is/was your chosen career path? (*Tick one*)
Response options: Extremely well; Very well; Moderately well; Slightly well; Not at all well

4.F. How much information do you get about possible career paths from each of the following? (*Tick one per row*)
Response options: A great deal; A lot; A moderate amount; A little; None at all; Not applicable to me

1. My academic/research supervisor(s)
2. Senior academics in my department
3. My peers undertaking a [DPhil/PhD] at the [University of Oxford / UCL]
4. [OUCAGS / ACO]
5. Mentor(s)
6. Newsletters/publications from medical bodies (e.g. GMC, NIHR)
7. Other (please specify)

For peer review only

Box 2 (cont.)

5. Clinical Lectureships

5.A. Please tell us how accurately the following statement describes you: 'If I wanted, I could become a Clinical Lecturer in England, at an appropriate point after completing my [DPhil/PhD]'. (*Tick one*)
Response options: Extremely accurately; Very accurately; Moderately accurately; Slightly accurately; Not at all accurately

Note 1: respondents who said that, in their long-term career, they intended to work mainly in ''clinical service posts without teaching or research''@ Question 4.A. →**SKIP Question 5.B..**

Note 2: This question is intended to operationalise self-efficacy as described in Ajzen (1991) – see References.

5.B. How likely are you to seek a Clinical Lecturer post in England, at an appropriate point after you complete your [DPhil/PhD]? (*Tick one*)
Response options: Extremely likely; Very likely; Moderately likely; Slightly likely; Not at all likely

Note: This question is intended to operationalise intention (of seeking a CL post) as described in Ajzen (1991) – see References.

5.C. How much do you feel that you know about Clinical Lecturer posts in England (including how to apply and what the posts entail)? (*Tick one*)
Response options: A great deal; A lot; A moderate amount; A little; Nothing at all

6. About respondents

6.A. What is your sex? (*Tick one*)
Response options: Male; Female

Note: To check whether respondents were representative of the whole population, we used administrative data about the whole population of clinical doctoral students invited to respond to our questionnaire. At the University of Oxford, only administrative data about clinical doctoral students' sex (collected as ''male'' or ''female'') was available for use in our study. Data on gender was not available. To ensure that data from our questionnaire would be comparable to the administrative data, we asked participants about their sex rather than their gender. However, in the presentation and discussion of results, we opted to refer to data on whether respondents ticked themselves as being male or female as ''gender'' because this, not their biological sex, is the relevant issue.

6.B. In which year were you born?
[text box: ''19_______'']

For peer review only - http://bmjopen.bmj.com/site/about/guidelines.xhtml

Box 2 (cont.)

6.C. Do you have a mentor, or mentors, who you meet regularly? By mentor we mean someone who is not your line manager or supervisor, and who helps you develop your knowledge, work or thinking. (*Tick one*)

1. I have a mentor/mentors, as part of a formalised mentoring relationship
2. I have an informal mentor/mentors
3. I do not have a mentor, but would like to find one
4. I do not have a mentor and do not feel the need, or have the time, for one
5. Other (please specify)

6.D. For fees purposes, what is your normal place of residence registered as? (*Tick one*)
Response options: Home (UK); Other European Union; Non-European Union

6.E. In which calendar year did you start your [DPhil/PhD] programme? (*Tick one*)
Response options: 2011 or earlier; 2012; 2013; 2014; 2015

For peer review only

Box 3 – Analysis of open questions

A. A number of closed questions had a final open item inviting participants to add their own response. However, none of these open items yielded recurrent themes when responses were reviewed by JL (Oxford data) and VR (UCL data).

B. The question "what might make a clinical academic career more attractive to you?" did yield recurrent themes and analysis was as follows:

1) JL produced a coding frame based on the Oxford data.
2) In a face-to-face meeting, JL and VR revised the coding frame with reference to data from both universities, and then used it to double-code anonymised responses from both Oxford and UCL. Any differences in coding between the researchers were discussed and resolved jointly.

Responses centred around two themes:

- **better post availability/a better career pathway** (mentioned by 38%, n=120 of all respondents), for example:

  "*Clearer pathways for balancing clinical training and research. As it is, it is very disruptive to either part of the career to pursue a clinical academic pathway.*" (Oxford participant)

  "*Availability of suitable posts, allowing for my (slightly unusual) combination of clinical specialty and research interests.*" (Oxford participant)

  "*Clear pathway for post-doctoral funding e.g buffer period between PhD completion and establishing independence*." (UCL participant)

  "*If there were more opportunities created by universities to fund part time research integrated with clinical consultant roles; as one nears the end of training and a senior lectureship seems very very unlikely, the choice is either struggle on and ultimately fail or drop research*." (UCL participant)

- **greater career/funding security** (mentioned by 28%, n=90 of all participants), for example:

  "*Availability of longer term funding and job security*" (Oxford participant)

  "*More financial and job security - applying for grants every 5 years or losing your job is not appealing. NHS consultants have far more job security once appointed*." (UCL participant)

For peer review only

Box 4 – Additional information about respondents

A. Gender and fee status of clinical doctoral students: percentages by university and for all respondents

	Oxford (N=170)		UCL (N=150)		Total (N=320)	
**Gender**	**n**	***%***	**n**	***%***	**n**	***%***
Male	108	***64***	66	***44***	174	***54***
Female	61	***36***	81	***54***	142	***44***
No response	1	***1***	3	***2***	4	***1***
**Fee status**	**n**	***%***	**n**	***%***	**n**	***%***
UK/EU	131	***77***	148	***99***	279	***87***
Overseas	33	***19***	-	***-***	33	***10***
No response	6	***4***	2	***1***	8	***3***

Note: Due to rounding, percentages may not add to 100.

B. Specialty of respondents: percentages by university and for all respondents

	Oxford (N=170)		UCL (N=150)		Total (N=320)	
**Specialties[§]**	**n**	***%***	**n**	***%***	**n**	***%***
Medicine	70	***41***	80	***53***	150	***47***
Surgery	30	***18***	11	***7***	41	***13***
Other	54	***32***	53	***35***	107	***33***
None	16	***9***	6	***4***	22	***7***

Note: Due to rounding, percentages may not add to 100.
[§] Specialties were grouped under "medicine" and "surgery" according to the groupings used *in* GMC. *National training survey 2013: undermining* London: General Medical Council, 2013;13-14 http://www.gmc-uk.org/NTS_2013_autumn_report_undermining.pdf_54275779.pdf (accessed 11 May 2017). In addition, "infectious diseases and microbiology" was classified as "medicine"; any sub-type of "surgery" not listed in the above document (e.g. "transplant surgery" or "paediatric surgery") was classed as "surgery".

For peer review only

Box 5 – Respondents' predoctoral involvement in medical research and reason for the doctorate: percentages by gender and for all respondents

	Males‡ (N=174)		Females‡ (N=142)		Total (N=320)	
	n	%	n	%	n	%
**Previous involvement in medical research‡‡**						
High involvement level *(intercalated degree and post(s) with research*, and possibly "other" experience, too)*	34	20	28	20	62	19
Some involvement *(intercalated degree or post(s) with research*, possibly with other experience, or "other" experience)*	112	64	91	64	206	64
No involvement	22	13	15	11	38	12
No response	6	3	8	6	14	4
**Main reason for doctorate**						
Interest in area of research	85	49	60	42	146	46
Improving prospects for an academic/research career	70	40	61	43	134	42
Improving prospects for a clinical career outside academia	16	9	17	12	33	10
Other	3	2	4	3	7	2

Note: Due to rounding, column percentages may not add to 100.
‡Four respondents did not provide their gender.
‡‡ More than one answer could be given to the question about involvement in medical research prior to the DPhil/PhD – see Box 2, Question 2.A..
*The following response options were considered to be 'posts with research': research rotation as an Academic Foundation Programme doctor; research block or regular release as an Academic Clinical Fellow (England, Wales, Northern Ireland); research block or regular release as a Clinical Research Fellow (Scotland); other post with time for research.

For peer review only

Box 6 – Variables not associated with wanting a clinical academic career (CAC) *vs.* having other plans or being undecided: statistically *non*-significant X^2 tests

		Respondents wanting a CAC		X^2 test		
**Variables**	**Groupings**	**%**	**n / N**	**df**	**X^2**	**p**
**Gender**	Male	55	96 / 174	1	3.430	.064
	Female	45	63 / 141			
**Fee status**	UK/EU	50	139 / 278	1	.027	.869
	Non-EU (Overseas)	52	17 / 33			
**Age**	<30 years	49	22 / 45	2	3.370	.185
	30-35 years	55	96 / 174			
	36 years and over	40	19 / 47			
**Training stage**	Pre-specialty	63	24 / 38	2	3.072	.215
	In specialty	48	108 / 224			
	Completed	47	27 / 57			
**Encouragement from DPhil/PhD supervisor(s) to reflect on career development needs**	A great deal/A lot	53	79 / 150	2	.927	.629
	A moderate amount	47	44 / 94			
	A little/None at all	48	36 / 75			
Influences on long-term career thinking:						
**Wanting a career that fits my domestic circumstances**	A great deal/A lot	51	89 / 175	2	.733	.693
	A moderate amount	51	53 / 105			
	A little/Not at all	43	16 / 37			
**Wanting to make a difference to patients**	A great deal/ A lot	52	140 / 267	2	3.951	.139
	A moderate amount	38	15 / 40			
	A little/ Not at all	36	4 / 11			
**Wanting a career with acceptable working hours/conditions**	A great deal/A lot	46	80 / 174	2	2.494	.287
	A moderate amount	55	60 / 109			
	A little/Not at all	54	19 / 35			
**Future financial prospects**	A great deal/A lot	51	62 / 122	2	.457	.796
	A moderate amount	51	64 / 125			
	A little/Not at all	47	33 / 71			
**Advice from others**	A great deal/A lot	57	39 / 69	2	3.602	.165
	A moderate amount	51	84 / 164			
	A little/Not at all	42	35 / 84			
**Exposure to role models**	A great deal/A lot	55	85 / 156	2	2.668	.263
	A moderate amount	46	48 / 105			
	A little/Not at all	45	25 / 56			

For peer review only

Box 6 *(continued)*

		Respondents wanting a CAC		X^2 test		
**Variables**	**Groupings**	**%**	**n / N**	**df**	**X^2**	**p**
Sources of information about possible career paths (amount of information received):						
**Academic/research supervisor(s)**	A great deal/A lot	55	57 / 104	2	2.123	.346
	A moderate amount	51	55 / 109			
	A little/None at all/N/A	45	47 / 105			
**Senior academics in department**	A great deal/A lot	57	36 / 63	2	5.557	.062
	A moderate amount	56	53 / 94			
	A little/None at all/N/A	44	70 / 161			
**Peers at same university**	A great deal/A lot	55	36 / 66	2	1.388	.500
	A moderate amount	52	56 / 108			
	A little/None at all/N/A	47	67 / 144			
**Newsletters /publications**	A great deal/A lot	54	15 / 28	2	2.589	.274
	A moderate amount	59	37 / 63			
	A little/None at all/N/A	48	107 / 225			
Mentoring:						
**Formal/informal mentor, regularly met**	Yes	51	91 / 180	1	.052	.820
	No	49	67 / 136			

For peer review only

Box 7 – Factors influencing respondents' thinking about their long-term career: percentages for those who want a clinical academic career (CAC) and those who do not/are undecided, and for all respondents

	Respondents wanting a CAC‡				Respondents *not* wanting a CAC‡				Total (N=320)	
	Males (N=96)		Females (N=63)		Males (N=78)		Females (N=78)			
	n	%	n	%	n	%	n	%	n	%
**Enthusiasm/commitment**										
A great deal/A lot	94	***98***	62	***98***	71	***91***	70	***90***	301	***94***
(A great deal)*	(67)	***(70)***	(40)	***(64)***	(38)	***(49)***	(43)	***(55)***	(191)	***(60)***
A moderate amount	2	***2***	1	***2***	6	***8***	7	***9***	17	***5***
A little/Not at all	0	***0***	0	***0***	0	***0***	1	***1***	1	***<1***
No response	-	-	-	-	1	***1***	-	-	1	***<1***
**Wanting intellectual stimulation**										
A great deal/A lot	95	***99***	62	***98***	61	***78***	63	***81***	285	***89***
(A great deal)*	(60)	***(63)***	(40)	***(64)***	(28)	***(36)***	(31)	***(40)***	(160)	***(50)***
A moderate amount	1	***1***	1	***2***	15	***19***	14	***18***	32	***10***
A little/Not at all	0	***0***	0	***0***	1	***1***	1	***1***	2	***1***
No response	-	-	-	-	1	***1***	-	-	1	***<1***
**Wanting to make a difference to patients**										
A great deal/A lot	82	***85***	58	***92***	59	***76***	65	***83***	268	***84***
(A great deal)*	(52)	***(54)***	(29)	***(46)***	(30)	***(39)***	(36)	***(46)***	(148)	***(46)***
A moderate amount	11	***12***	4	***6***	14	***18***	10	***13***	40	***13***
A little/Not at all	3	***3***	1	***2***	4	***5***	3	***4***	11	***3***
No response	-	-	-	-	1	***1***	-	-	1	***<1***
**Self-appraisal of own skills/aptitudes**										
A great deal/A lot	63	***66***	44	***70***	37	***47***	46	***59***	192	***60***
A moderate amount	23	***24***	15	***24***	32	***41***	25	***32***	98	***31***
A little/Not at all	10	***10***	4	***6***	8	***10***	7	***9***	29	***9***
No response	-	-	-	-	1	***1***	-	-	1	***<1***
**Wanting a career that fits my domestic circumstances**										
A great deal/A lot	48	***50***	41	***65***	38	***49***	46	***59***	176	***55***
A moderate amount	36	***38***	17	***27***	25	***32***	26	***33***	105	***33***
A little/Not at all	11	***12***	5	***8***	14	***18***	6	***8***	37	***12***
No response	1	***1***	-	-	1	***1***	-	-	2	***1***
**Wanting a career with acceptable working hours/conditions**										
A great deal/A lot	42	***44***	38	***60***	38	***49***	54	***69***	174	***54***
A moderate amount	41	***43***	19	***30***	28	***36***	19	***24***	110	***34***
A little/Not at all	13	***14***	6	***10***	11	***14***	5	***6***	35	***11***
No response	-	-	-	-	1	***1***	-	-	1	***<1***

Box 7 (continued)

	Respondents wanting a CAC‡				Respondents *not* wanting a CAC‡				Total (N=320)	
	Males (N=96)		Females (N=63)		Males (N=78)		Females (N=78)			
	n	*%*	*n*	*%*	*n*	*%*	*n*	*%*	n	*%*
**Exposure to role models**										
A great deal/A lot	52	***54***	33	***52***	34	***44***	35	***45***	157	***49***
A moderate amount	29	***30***	19	***30***	27	***35***	28	***36***	105	***33***
A little/Not at all	14	***15***	11	***18***	16	***21***	15	***19***	56	***18***
No response	1	***1***	-	-	1	***1***	-	-	2	***1***
**Career promotion and prospects**										
A great deal/A lot	53	***55***	32	***51***	35	***45***	25	***32***	146	***46***
A moderate amount	28	***29***	23	***37***	26	***33***	38	***49***	119	***37***
A little/Not at all	15	***16***	8	***13***	16	***21***	15	***19***	54	***17***
No response	-	-	-	-	1	***1***	-	-	1	***<1***
**Future financial prospects**										
A great deal/A lot	41	***43***	21	***33***	33	***42***	25	***32***	122	***38***
A moderate amount	39	***41***	25	***40***	26	***33***	34	***44***	126	***39***
A little/Not at all	16	***17***	17	***27***	18	***23***	19	***24***	71	***22***
No response	-	-	-	-	1	***1***	-	-	1	***<1***
**Advice from others**										
A great deal/A lot	23	***24***	16	***25***	19	***24***	11	***14***	69	***22***
A moderate amount	51	***53***	33	***52***	34	***44***	42	***54***	164	***51***
A little/Not at all	21	***22***	14	***22***	24	***31***	25	***32***	84	***26***
No response	1	***1***	-	-	1	***1***	-	-	3	***1***
**Lack of posts in other possible career paths**										
A great deal/A lot	7	***7***	1	***2***	8	***10***	13	***17***	30	***9***
A moderate amount	9	***9***	10	***16***	15	***19***	19	***24***	53	***17***
A little/Not at all<br>(Not at all)*	80<br>(47)	***83***<br>***(49)***	52<br>(43)	***83***<br>***(68)***	54<br>(29)	***69***<br>***(37)***	46<br>(26)	***59***<br>***(33)***	236<br>(147)	***74***<br>***(46)***
No response	-	-	-	-	1	***1***	-	-	1	***<1***

Notes: Due to rounding, column percentages may not add to 100. A minority of respondents rated "other" factors as part of the final, open item of the question; as no recurrent themes were identified, this item is not included in the table.
‡Base: All respondents in group who provided their gender and responded to the question about their career plans (N=315).
*Some variables were considerably skewed. Where the mode for the whole group of respondents (total) was either ''a great deal'' or ''not at all'', figures for the modal response-only are provided in brackets.

For peer review only - http://bmjopen.bmj.com/site/about/guidelines.xhtml

Box 8 – Variables not associated with likelihood of seeking a CL: statistically *non*-significant X^2 tests

		Respondents extremely/very likely to seek a CL		X^2 test		
**Variables**	**Groupings**	**%**	**n / N**	**df**	**X^2**	**p**
**Gender**	Male	46	78 / 171	1	.788	.375
	Female	41	56 / 138			
**Encouragement from DPhil/PhD supervisor(s) to reflect on career development needs**	A great deal/A lot	48	70 / 147	2	2.832	.243
	A moderate amount	37	34 / 92			
	A little/None at all	41	30 / 74			
Sources of information about possible career paths (amount of information received):						
**Peers**	A great deal/A lot	44	29 / 66	2	2.626	.269
	A moderate amount	49	51 / 105			
	A little/None at all/N/A	38	54 / 141			
**Newsletters/publications**	A great deal/A lot	50	14 / 28	2	1.312	.519
	A moderate amount	47	29 / 62			
	A little/None at all/N/A	41	90 / 220			
Mentoring:						
**Formal/informal mentor, regularly met**	Yes	44	77 / 177	1	.013	.910
	No	43	57 / 133			

For peer review only

Box 9 – Sources of information about possible career paths: by gender for those who want a clinical academic career (CAC) and those who have other plans/are undecided, and for all respondents

	Respondents wanting a CACǂ				Respondents *not* wanting a CACǂ				Total (N=320)	
	Males (N=96)		Females (N=63)		Males (N=78)		Females (N=78)			
	n	%	*n*	*%*	*n*	*%*	*n*	*%*	n	*%*
**Academic/research supervisor(s)**										
Great deal/A lot	39	**41**	*18*	**29**	23	***30***	*23*	***30***	105	***33***
A moderate amount	29	**30**	*26*	**41**	32	***41***	*21*	***27***	109	***34***
A little/None/N/A	28	**29**	*19*	**30**	23	***30***	*33*	***42***	105	***33***
No response	-	-	-	-	-	-	*1*	***1***	1	***<1***
**Senior academics in my department**										
Great deal/A lot	27	**28**	9	**14**	17	***22***	9	***12***	63	***20***
A moderate amount	29	**30**	24	**38**	22	***28***	18	***23***	94	***29***
A little/None/N/A	40	**42**	30	**48**	39	***50***	50	***64***	162	***51***
No response	-	-	-	-	-	-	1	***1***	1	***<1***
**Peers undertaking a doctorate at Oxford/UCL**										
Great deal/A lot	29	**30**	7	**11**	19	***24***	11	***14***	66	***21***
A moderate amount	33	**34**	23	**37**	24	***31***	27	***35***	108	***34***
A little/None/N/A	34	**35**	33	**52**	35	***45***	39	***50***	145	***45***
No response	-	-	-	-	-	-	1	***1***	1	***<1***
**OUCAGS/ACO**										
Great deal/A lot	13	**14**	9	**14**	3	***4***	2	***3***	27	***8***
A moderate amount	21	**22**	12	**19**	11	***14***	7	***9***	52	***16***
A little/None/N/A	61	**64**	42	**67**	64	***82***	65	***83***	236	***74***
(Not applicable) *	(4)	**(4)**	(6)	**(10)**	(12)	***(15)***	(4)	***(5)***	(26)	***(8)***
No response	1	**1**	-	-	-	-	4	***5***	5	***2***
**Mentors**										
Great deal/A lot	28	**29**	22	**35**	18	***23***	10	***13***	78	***24***
A moderate amount	28	**29**	12	**19**	25	***32***	16	***21***	81	***25***
A little/None/N/A	39	**41**	29	**46**	35	***45***	49	***63***	157	***49***
(Not applicable) *	(15)	**(16)**	(15)	**(24)**	(11)	***(14)***	(17)	***(22)***	(60)	***(19)***
No response	1	**1**	-	-	-	-	3	***4***	4	***1***
**Newsletters/publications from medical bodies**										
Great deal/A lot	11	**12**	4	**6**	6	***8***	6	***8***	28	***9***
A moderate amount	23	**24**	14	**22**	11	***14***	14	***18***	64	***20***
A little/None/N/A	62	**65**	45	**71**	61	***78***	55	***71***	225	***70***
No response	-	-	-	-	-	-	3	***4***	3	***1***

Note: Due to rounding, column percentages may not add to 100.

ǂBase: All respondents in group who provided their gender and responded to the question about their career plans (N=315).

**Question attracted ''not applicable'' responses from over 3% of respondents, so ''not applicable''-only responses are provided separately in brackets.*

For peer review only - http://bmjopen.bmj.com/site/about/guidelines.xhtml

STROBE Statement—Checklist of items that should be included in reports of ***cross-sectional studies***

	Item No	Recommendation
**Title and abstract**	1	(*a*) Indicate the study's design with a commonly used term in the title or the abstract [p.1 , in *Title*; p.2, in *Abstract*]
		(*b*) Provide in the abstract an informative and balanced summary of what was done and what was found [p.2, *Abstract*]
**Introduction**		
Background/rationale	2	Explain the scientific background and rationale for the investigation being reported [pp.4-5, *Introduction*]
Objectives	3	State specific objectives, including any prespecified hypotheses [pp.4-5, *Introduction*]
**Methods**		
Study design	4	Present key elements of study design early in the paper [p.6, *Method – Design and procedures*]
Setting	5	Describe the setting, locations, and relevant dates, including periods of recruitment, exposure, follow-up, and data collection [p.6, *Method – Design and procedures*]
Participants	6	(*a*) Give the eligibility criteria, and the sources and methods of selection of participants [p.6, *Method – Design and procedures*]
Variables	7	Clearly define all outcomes, exposures, predictors, potential confounders, and effect modifiers. Give diagnostic criteria, if applicable [p.6, *Method – Instruments*]
Data sources/ measurement	8*	For each variable of interest, give sources of data and details of methods of assessment (measurement). Describe comparability of assessment methods if there is more than one group [p.6, *Method – Instruments*]
Bias	9	Describe any efforts to address potential sources of bias [p.8, *Results – Characteristics of clinical doctoral students: Demographics and training*]
Study size	10	Explain how the study size was arrived at [p.6, *Method – Design and procedures*]
Quantitative variables	11	Explain how quantitative variables were handled in the analyses. If applicable, describe which groupings were chosen and why [pp.6-7 *Method – Analysis*]
Statistical methods	12	(*a*) Describe all statistical methods, including those used to control for confounding [pp.6-7, *Method – Analysis*]
		(*b*) Describe any methods used to examine subgroups and interactions [pp.6-7, *Method – Analysis*]
		(*c*) Explain how missing data were addressed [n/a]
		(*d*) If applicable, describe analytical methods taking account of sampling strategy [n/a]
		(*e*) Describe any sensitivity analyses [n/a]

For peer review only

For peer review only - http://bmjopen.bmj.com/site/about/guidelines.xhtml

**Results**		
Participants	13*	(a) Report numbers of individuals at each stage of study—eg numbers potentially eligible, examined for eligibility, confirmed eligible, included in the study, completing follow-up, and analysed [p.6, *Method – Design and procedures* and p.8-9; *Results – Characteristics of clinical doctoral students: Response rates*; Supplement, Box 1]
		(b) Give reasons for non-participation at each stage [p.8, *Results – Characteristics of clinical doctoral students: Response rates*; Supplement, Box1]
		(c) Consider use of a flow diagram [Supplement, Box 1]
Descriptive data	14*	(a) Give characteristics of study participants (eg demographic, clinical, social) and information on exposures and potential confounders [p.8-9, *Results – Characteristics of clinical doctoral students*]
		(b) Indicate number of participants with missing data for each variable of interest [p.8-9, *Results – Characteristics of clinical doctoral students: Demographics and training*; p.10, Table 1; p.15, Table 4]
Outcome data	15*	Report numbers of outcome events or summary measures [*Results*: p.10, Table 1; p.15, Table 4]
Main results	16	(*a*) Give unadjusted estimates and, if applicable, confounder-adjusted estimates and their precision (eg, 95% confidence interval). Make clear which confounders were adjusted for and why they were included [n/a]
		(*b*) Report category boundaries when continuous variables were categorized [n/a]
		(*c*) If relevant, consider translating estimates of relative risk into absolute risk for a meaningful time period [n/a]
Other analyses	17	Report other analyses done—eg analyses of subgroups and interactions, and sensitivity analyses [*Results*: p.12, Table 2; p.16, Table 5]
**Discussion**		
Key results	18	Summarise key results with reference to study objectives [p.18, *Discussion*]
Limitations	19	Discuss limitations of the study, taking into account sources of potential bias or imprecision. Discuss both direction and magnitude of any potential bias [p.19, *Discussion – Strengths and limitations of the study*]
Interpretation	20	Give a cautious overall interpretation of results considering objectives, limitations, multiplicity of analyses, results from similar studies, and other relevant evidence [pp.18-20, *Discussion*]
Generalisability	21	Discuss the generalisability (external validity) of the study results [p.19, *Discussion –Strengths and limitations of the study*]
**Other information**		
Funding	22	Give the source of funding and the role of the funders for the present study and, if applicable, for the original study on which the present article is based [p.21]

For peer review only

For peer review only - http://bmjopen.bmj.com/site/about/guidelines.xhtml

*Give information separately for exposed and unexposed groups.

Note: An Explanation and Elaboration article discusses each checklist item and gives methodological background and published examples of transparent reporting. The STROBE checklist is best used in conjunction with this article (freely available on the Web sites of PLoS Medicine at http://www.plosmedicine.org/, Annals of Internal Medicine at http://www.annals.org/, and Epidemiology at http://www.epidem.com/). Information on the STROBE Initiative is available at www.strobe-statement.org.

For peer review only

For peer review only - http://bmjopen.bmj.com/site/about/guidelines.xhtml

The clinical academic workforce of the future: a cross-sectional study of factors influencing career decision-making amongst clinical PhD students at two research-intensive UK universities

Journal:	*BMJ Open*
Manuscript ID	bmjopen-2017-016823.R2
Article Type:	Research
Date Submitted by the Author:	03-Jul-2017
Complete List of Authors:	Lopes, Joana; University of Oxford, Oxford University Clinical Academic Graduate School<br>Ranieri, Veronica; University College London, School of Life and Medical Sciences; University College London, Department of Applied Health Research<br>Lambert, Trevor; University of Oxford, Nuffield Department of Population Health<br>Pugh, Chris; University of Oxford, Oxford University Clinical Academic Graduate School<br>Barratt, Helen; University College London, Department of Applied Health Research<br>Fulop, Naomi; University College London, Department of Applied Health Research<br>Rees, Geraint; University College London, School of Life and Medical Sciences<br>Best, Denise; University of Oxford, Oxford University Clinical Academic Graduate School
**Primary Subject Heading**:	Medical education and training
Secondary Subject Heading:	Evidence based practice
Keywords:	EDUCATION & TRAINING (see Medical Education & Training), MEDICAL EDUCATION & TRAINING, Human resource management < HEALTH SERVICES ADMINISTRATION & MANAGEMENT

SCHOLARONE™
Manuscripts

The clinical academic workforce of the future: a cross-sectional study of factors influencing career decision-making amongst clinical PhD students at two research-intensive UK universities

Joana Lopes[1], Veronica Ranieri[2 3], Trevor Lambert[4], Chris Pugh[1], Helen Barratt[3], Naomi J Fulop[3], Geraint Rees[2], Denise Best[1]

[1]Oxford University Clinical Academic Graduate School, Room 3600 Medical Sciences Divisional Office, Level 3, John Radcliffe Hospital (Main Hospital), Oxford, OX3 9DU, UK
[2]School of Life and Medical Sciences, University College London, Gower Street, London WC1E 6BT, UK
[3] Department of Applied Health Research, University College London, Gower Street, London WC1E 6BT, UK
[4]Nuffield Department of Population Health, University of Oxford, Oxford OX3 7LF, UK

Correspondence to: Joana Lopes; joana.lopes@medsci.ox.ac.uk; T: 01865 289 465

Word count: 4,068

For peer review only

ABSTRACT

Objectives To examine clinical doctoral students' demographic and training characteristics, career intentions, career preparedness, and what influences them as they plan their future careers.

Design and setting Online cross-sectional census surveys at two research-intensive medical schools in England in 2015-16.

Participants All medically qualified PhD students (N=523) enrolled at the University of Oxford and University College London were invited to participate. We report on data from 320 participants (54% male and 44% female), who were representative by gender of the invited population.

Main outcome measures Career intentions.

Results Respondents were mainly in specialty training, including close to training completion (25%, n=80), and 18% (n=57) had completed training. Half (50%, n=159) intended to pursue a clinical academic career (CAC) and 62% (n=198) were at least moderately likely to seek a clinical lectureship (CL). However, 51% (n=163) had little or no knowledge about CL posts. Those wanting a CAC tended to have the most predoctoral medical research experience [X^2 (2, N=305) = 22.19, p=.0005]. Key reasons cited for not pursuing a CAC were the small number of senior academic appointments available, the difficulty of obtaining research grants, and work-life balance.

Conclusions Findings suggest that urging predoctoral clinicians to gain varied research experience whilst ensuring availability of opportunities, and introducing more flexible recruitment criteria for CL appointments, would foster CACs. As CL posts are often only open to those still in training, the many postdoctoral clinicians who have completed training, or nearly done so, do not currently gain the opportunity the post offers to develop as independent researchers. Better opportunities should be accompanied by enhanced career support for clinical doctoral students (e.g. to increase knowledge of CLs). Finally, ways to increase the number of senior clinical academic appointments should be explored since their lack seems to significantly influence career decisions.

Key words: clinical academic, academic medicine, clinical lectureship, physician scientist, career development

For peer review only - http://bmjopen.bmj.com/site/about/guidelines.xhtml

Strengths and limitations of this study

- This is the first UK study to describe the career aspirations of medically qualified PhD students and what influences them as they make their postdoctoral career plans.
- Other studies about the retention of postdoctoral clinical academics are predominantly North American and reliant on retrospective data.
- The study identifies factors which are associated with clinical PhD students' decision to apply for clinical lectureships (a pivotal post in clinical academic training in England).
- As it is a cross-sectional study, causality cannot be inferred from the associations we found between career plans and other variables.
- It is not possible to say whether respondents' intentions will be implemented as planned and findings may not generalise beyond research-intensive universities in England.

For peer review only

For peer review only - http://bmjopen.bmj.com/site/about/guidelines.xhtml

INTRODUCTION

Clinical academics (also known internationally as physician scientists) are vital to educating medical students and, as they bring a clinical perspective to research, to discovering and realising new insights into human disease.[1] Establishing a stable pipeline that develops clinical academics,[2] continues to be of global concern, for example in the US and Australia.[3,4] In the UK, anxieties date back at least to the Savill report[1] in 2000 and the subsequent Modernising Medical Careers report.[5] The establishment of the National Institute for Health Research (NIHR) in 2006, and similar developments in Scotland, Wales, and Northern Ireland, supported the formation of an integrated clinical academic training (IAT) pathway[6] designed to tackle concerns about dwindling clinical academic numbers by providing clinical training posts with protected research time. In England, clinical academic training opportunities start with Academic Foundation posts, continuing to predoctoral academic clinical fellowships (ACFs), doctoral training fellowships and, finally, postdoctoral clinical lectureships (CLs).[6] Despite these efforts, the latest report from the Medical Schools Council[7] states that more than half of UK medical schools report difficulties in recruitment to some clinical academic posts. Of additional concern is the small proportion of women who pursue clinical academic careers (CACs).[8]

A pivotal point common to clinical academic training pathways irrespective of country is the PhD.[6] Interestingly, recent career tracking of doctoral clinical training fellowships awarded by major UK research funders[9] suggests that only around a third of holders progress to a junior postdoctoral CL or the more senior clinician scientist post. (CLs are open to doctors in specialty training, whereas clinician scientists have completed their training and developed research independence.) A range of barriers and enablers have been identified as contributing to this issue, including matters relating to work-life balance, funding, mentorship and careers information.[9-12] Notwithstanding, there has been no work in the UK examining the characteristics of doctors undertaking PhDs or how they view their future career options, including why those wanting a CAC may not consider applying for a postdoctoral CL.[10,13]

In 2013, the Oxford University Clinical Academic Graduate School (OUCAGS) began a longitudinal study of its clinical PhD students to help understand these issues. The part of the study reported here was undertaken in 2015-16 in collaboration with University College London's (UCL) Academic Careers Office (ACO). Our aims were to: describe the demographic and clinical-training characteristics of both institutions' clinical PhD students; determine their long-term career plans (including how many want a CAC) and factors influencing these plans; examine their views regarding

For peer review only - http://bmjopen.bmj.com/site/about/guidelines.xhtml

CL posts, since these are the first postdoctoral posts on the established IAT pathway; and explore their preparedness for making career decisions.

For peer review only

For peer review only - http://bmjopen.bmj.com/site/about/guidelines.xhtml

METHOD

In reporting our study, we followed the STROBE guidelines.[14]

Design and procedures

We conducted a census survey of all medically qualified PhD students enrolled, at the beginning of autumn 2015, at two universities in England: the University of Oxford (hereafter ''Oxford'') and UCL. Students who were either suspended or had already submitted their thesis were excluded. MD/DM students were also excluded as their higher degree programme is often less structured and the survey questions may not apply in the same way. Each survey had ethical approval from its home institution and collected its data via its own online questionnaire between the end of October 2015 and the beginning of March 2016. Participants received: an email from senior academics introducing the study; an email invitation which offered an £8 Amazon voucher for participation; two email reminders at Oxford, three at UCL; and one postal reminder inviting them to complete either the online questionnaire or a paper version. The target population was identified from administrative records. Where we were unclear about students' qualifications, after the introductory email we sent an email checking qualifications and excluded those who responded that they were not medically qualified. A total of 523 eligible students were asked to participate (see STROBE diagrams in Supplement Box 1).

Instruments

OUCAGS developed the questionnaire in 2013, as part of its Clinical DPhil Paths longitudinal study (2013-2018) of the career plans of clinical PhD students. (''DPhil'' is the term for an Oxford PhD.) Questions were either newly created or adapted from existing questionnaires.[15,16] They covered respondents' characteristics and career plans, including: demographic variables, medical training, predoctoral involvement in research, reason for entering the doctorate, doctoral experience, intention to pursue a CAC and a CL in England (where the NIHR funds a large number of posts), and views regarding CL posts (including how attainable they think these posts are[17]). (See Box 2 in Supplement for details of questionnaire development, the questions reported below, and minor adaptations by UCL (e.g. use of ''PhD'' instead of ''DPhil'')).

Analysis

We merged anonymised Oxford and UCL datasets and analysed the data using SPSS 22. We used frequencies to profile respondents (e.g. age, fee status) as well as to compare respondents by gender and intention towards a CAC. For fee status, we grouped UK and EU respondents together as both were eligible for ''home'' fees and have equal employment rights within the UK. This also

For peer review only - http://bmjopen.bmj.com/site/about/guidelines.xhtml

preserved EU respondents' anonymity in file sharing as their number was small (n= 12 and 9 respondents, from Oxford and UCL, respectively).

We analysed responses to open questions using conventional content analysis[18] to identify themes and counted the number of participants mentioning each recurrent theme. Responses were double-coded by JL and VR in a face-to-face meeting, and any coding inconsistencies were discussed and resolved jointly (Box 3 in Supplement).

As a first step, the data were examined in detail. Then variables with data collected on five- or six-point scales were summarised into three categories (e.g. ''a great deal/a lot'', ''a moderate amount'', ''a little/none at all/not applicable'' – see Results tables), with the full set of response categories referred to where relevant (e.g. for very skewed variables). Using three categories meant that data could be more clearly presented and, in most cases, items from a same question could be entered into Chi-square tests with responses categorised in the same manner. Categories were further collapsed, where necessary, to meet the requirements of Chi-square tests for expected frequencies.[19]

We conducted Chi-square tests of association to determine which of a range of background, doctoral experience, and attitude variables were associated (at the .05 level of statistical significance) with two key career outcomes:

a) intention to pursue a CAC in the long-term (respondents saying CAC *vs.* those with other plans or undecided), and
b) likelihood of seeking a CL in England (extremely or very likely to seek such a post *vs.* not at all to moderately likely).

For statistically significant Chi-square tests, we referred to adjusted standardised residuals to assist with interpreting results. The higher a residual's value, the more a cell contributes to the Chi-square value.

For peer review only - http://bmjopen.bmj.com/site/about/guidelines.xhtml

RESULTS

Characteristics of clinical PhD students

Response rates

Response rates were 67% and 57% for the Oxford and the UCL surveys, respectively, giving 322 respondents in total. A more resource-intensive approach (e.g. telephone reminders/interviews) may have yielded higher response rates.[20] Only two Overseas (i.e. non-UK/EU) students responded to the UCL questionnaire and, to preserve their anonymity, their data were not shared with Oxford. This paper therefore reports on data from 320 clinical PhD students, of which 170 (53%) were enrolled at Oxford and 150 (47%) at UCL.

Demographics and training

Of the 320 respondents, 54% (n=174) were male, 44% (n=142) female, and 1% (n=4) did not specify. Analysis for non-response bias by gender showed no bias needed to be accounted for in the analyses. Indeed, respondents were representative of the combined populations of clinical PhD students of Oxford and UCL as, based on administrative data, 57% (n=299) of students invited to complete the questionnaire were male and 43% (n=224) were female.

Most respondents were aged 30-35 years (Table 1). Figures for UK/EU respondents only (N=279) are comparable to figures for all respondents as 52% (n=146) were male and only 10% (n=28) were under 30 years. However, amongst the Overseas (Oxford) students (N=33), more respondents were male (70%, n=23) and under 30 (49%, n=16) (see Box 4 in Supplement for additional information).

Most participants were in specialty training years 3 to 8 (ST3-8), i.e. had done four or more years of clinical training after medical school and entered a specialty; this included 80 respondents (25% of the total) in years 6 to 8 (ST6-8), who were close to completing training. Approximately one-fifth had completed their training (Table 1).

The majority (84%, n=268) of participants had been involved in medical research prior to their doctorate, but over one-tenth (12%, n=38) had no involvement (see Box 5 in Supplement for additional information). This was similar at Oxford and UCL. The most common type of predoctoral medical research experience was an intercalated degree, that is, a research degree taken during the undergraduate years (48% of respondents, n=154). Also, over one-fifth (21%, n=67) of participants had held a predoctoral fellowship (i.e. an ACF in England, Wales, or Northern Ireland (n=65), or a clinical research fellowship (CRF) in Scotland (n=2)). Additionally, some respondents had: completed an Academic Foundation Programme (19%, n=60), held other posts with time for research (12%,

For peer review only - http://bmjopen.bmj.com/site/about/guidelines.xhtml

n=37), and/or had "other" research-related experiences, such as research towards an MSc thesis or a research assistantship (31%, n=100).

Reason for entering a doctoral programme

Respondents' main reason for undertaking a doctorate centred upon interest in their area of research (46%, n=146) or improving their prospects for an academic/research career (42%, n=134). However, improving their prospects for a clinical career *outside* academia was also mentioned by some (10%, n=33) (see Box 5 in Supplement for additional information). Respondents who had completed their specialist training were particularly likely to cite interest in their area of research (56%, n=32), although over one-third (35%, n=20) specifically wanted to improve their prospects for an academic/research career.

For peer review only

For peer review only - http://bmjopen.bmj.com/site/about/guidelines.xhtml

Table 1 – Respondents' age, training level and long-term career plans: percentages by gender and for all respondents

	All respondentsǂ		
	**Males** (N=174) %	**Females** (N=142) %	**Total** (N=320) %
**Age**			
<30	17	11	14
30-35	56	54	55
36+	14	16	15
No response	13	20	17
**Highest training stage completed or underway***			
Pre-Foundation, in Foundation, or post-Foundation programme	14	10	12
In ST1-2, CT or ACCS, *or* completed ST1-2, CT or ACCS	12	8	10
In ST3-8	56	57	56
ST level not specified	3	4	4
Completed specialist training	16	21	18
**Long-term career plans**			
Clinical academic posts	55	44	50
Clinical service posts with some teaching and research	23	23	23
Clinical service posts with some research	9	9	9
Clinical service posts with some teaching	2	1	2
Clinical service posts *without* teaching or research	1	1	1
Research-only posts	2	3	3
Undecided	8	15	11
Other	1	4	2
No response	-	1	<1

Note: Due to rounding, column percentages may not add to 100.
ǂFour respondents did not provide their gender.
*In the UK, following medical school and a two-year Foundation programme, doctors enter specialist training (ST). The first two years of specialty training are usually referred to as ST1-2, but some specialties have, before specialist training, a core training (CT) or acute care common stem (ACCS) programme. However, this is highly dependent on specialty.

Long-term career planning

Career plans

Half of all respondents expressed a desire to pursue a CAC in the long term, and around a third intended to work in clinical posts with some research, or some teaching and research (Table 1). The figure for respondents wanting a CAC was higher amongst former ACFs/CRFs (63%, n=42). Over one-tenth of respondents, including 13% (n=9) of former ACFs/CRFs and 12% (n=27) of doctors in specialty training, were undecided about their long-term career, although very few wanted purely

For peer review only - http://bmjopen.bmj.com/site/about/guidelines.xhtml

clinical posts or clinical posts with teaching. The proportion of participants undecided or with each type of career plan was similar across doctoral year groups (Year 1 to Year 4 and above).

Chi-square tests showed that greater predoctoral involvement in medical research, and pursuing a doctorate mainly to improve academic/research-career prospects, were each associated with wanting a CAC (Table 2). In contrast, there was no statistically significant association between whether a respondent was planning a CAC and: gender, fee status (UK/EU *vs.* Overseas), age or training stage (Box 6 in Supplement).

For peer review only

For peer review only - http://bmjopen.bmj.com/site/about/guidelines.xhtml

Table 2 – Variables associated with wanting a clinical academic career (CAC) *vs.* having other plans or being undecided: statistically significant X^2 tests

Variables	Groupings	Respondents wanting a CAC %	n / N	Adjusted residuals‡	X^2 test df	X^2	p
**Predoctoral involvement in medical research**	**No involvement**	26	10 / 38	-3.0	2	22.193	.0005
	**Some involvement** *(intercalated degree or post(s) with research, possibly with other experience, or "other" experience)*§	46	95 / 205	-1.4			
	**High involvement level** *(intercalated degree and post(s) with research, and possibly "other" experience, too)*§	73	45 / 62	4.1			
**Main reason for doctorate**	Interest in my area of research	49	71 / 146	-.4	2	25.515	.0005
	Improving prospects for an academic/research career	61	82 / 134	3.5			
	Other	15	6 / 39	-4.6			
Influences on long-term career thinking:							
**Wanting intellectual stimulation***	A great deal/A lot	55	157 / 284	5.4	1	29.640	.0005
	A moderate amount/A little/Not at all	6	2 / 34	-5.4			
**Enthusiasm /commitment***	A great deal/ A lot	52	156 / 300	2.9	1	8.480	.004
	A moderate amount/ A little/ Not at all	17	3 / 18	-2.9			
**Self-appraisal of own skills/aptitudes**	A great deal/A lot	56	107 / 191	2.6	2	7.743	.021
	A moderate amount	39	38 / 98	-2.7			
	A little/Not at all	48	14 / 29	-.2			
**Career promotion and prospects**	A great deal/A lot	58	85 / 146	2.7	2	7.300	.026
	A moderate amount	43	51 / 118	-1.9			
	A little/Not at all	43	23 / 54	-1.2			
**Lack of posts in other possible career paths**	A great deal/A lot	27	8 / 30	-2.7	2	14.357	.001
	A moderate amount	36	19 / 53	-2.3			
	A little/Not at all	56	132 / 235	3.7			
CL posts:							
**CL knowledge**	A great deal/A lot	73	27 / 37	3.0	2	19.198	.0005
	A moderate amount	59	69 / 118	2.3			
	A little/Nothing at all	39	63 / 162	-4.1			
**CL attainability (accuracy of statement that CL is attainable)**	Extremely/Very accurate	63	60 / 96	2.9	2	16.079	.0005
	Moderately accurate	54	57 / 105	1.1			
	Slightly/Not at all accurate	36	42 / 117	-3.8			
Sources of information about possible career paths (amount of information received):							
**OUCAGS/ACO**	A great deal/A lot	82	22 / 27	3.4	2	18.917	.0005
	A moderate amount	65	33 / 51	2.2			
	A little/None at all/N/A	44	103 / 236	-4.1			
**Mentor(s)**	A great deal/A lot	64	50 / 78	2.8	2	8.778	.012
	A moderate amount	49	40 / 81	-.2			
	A little/None at all/N/A	44	68 / 156	-2.3			

‡ For explanation and interpretation see Method.
§The following were considered "posts with research": Academic Foundation Programme posts; Academic Clinical Fellow (England, Wales, Northern Ireland); Clinical Research Fellow (Scotland); and responses of "other post with time for research".
*Due to the small numbers selecting "A moderate amount", "A little" and "Not at all", these categories were merged in order to meet X^2 test assumptions (see Method).

For peer review only - http://bmjopen.bmj.com/site/about/guidelines.xhtml

Enthusiasm and commitment to a given career path, as well as wanting intellectual stimulation and to make a difference to patients, were major influencing factors in respondents' long-term career planning (Table 3, and Box 7 in Supplement for further details). In addition, having acceptable working hours and conditions was especially important to respondents who were undecided about their long-term career, with 78% (n=28) of this group saying this had a great deal or a lot of importance.

In Chi-square tests the following were positively associated with wanting a CAC (Table 2): wanting intellectual stimulation, enthusiasm/commitment, self-appraisal of own skills/aptitudes, and career promotion and prospects. Also, a perceived lack of posts in other possible career paths was negatively associated with planning a CAC, possibly as respondents wanting a CAC can always return to a full-time clinical career. There was no statistically significant association between planning a CAC and other long-term career planning factors examined (Box 6 in Supplement).

Table 3 – Factors influencing respondents' thinking about their long-term career: percentage saying ''a great deal'' or ''a lot'', by whether respondents want a clinical academic career (CAC) *vs.* having other plans or being undecided, and for all respondents

	**Respondents wanting a CAC‡** (N=159) **%**	**Respondents *not* wanting a CAC‡** (N=160) **%**	**Total** (N=320) **%**
Enthusiasm/commitment<br>*(A great deal)**	98<br>*(67)*	90<br>*(52)*	94<br>*(60)*
Wanting intellectual stimulation<br>*(A great deal)**	99<br>*(63)*	79<br>*(38)*	89<br>*(50)*
Wanting to make a difference to patients<br>*(A great deal)**	88<br>*(51)*	79<br>*(41)*	84<br>*(46)*
Self-appraisal of own skills/aptitudes	67	53	60
Wanting a career that fits my domestic circumstances	56	54	55
Wanting a career with acceptable working hours/conditions	50	59	54
Exposure to role models	54	44	49
Career promotion and prospects	54	38	46
Future financial prospects	39	38	38
Advice from others	25	19	22
Lack of posts in other possible career paths	5	14	9

‡Base: All participants (N=319) who responded to the question about their career plans.
*Some variables were considerably skewed. Where the mode for the whole group of respondents (total) was ''a great deal'', figures for the modal response-only are provided in brackets.

For peer review only - http://bmjopen.bmj.com/site/about/guidelines.xhtml

Drawbacks of a clinical academic career

Respondents not intending to work in either clinical academia or research-only posts (N=153) were asked how much each of a range of factors might be a reason for excluding a CAC from their long-term career plans. This group included over a third (37%, n=25) of former ACFs/CRFs. Nearly two-thirds of the group (66%, n=101) said that the difficulty of obtaining research grants had a great deal or a lot of weight in their planning. Other factors with considerable weight were (% saying ''a great deal'' or ''a lot''):

- the small number of senior academic appointments available (50%, n=76)
- the competing pressures from service, teaching and research (47%, n=72)
- the limited future financial prospects compared to alternative careers (34%, n=52)
- needing work arrangements that are compatible with their caring responsibilities (30%, n=46, including 45%, n=34, of women but only 15%, n=11, of men), and
- family circumstances making them unwilling to move for work (27%, n=42).

Responses to the open question "what might make a clinical academic career more attractive to you?", asked of all participants, confirmed that issues around obtaining research grants, securing posts, and career progression are of particular concern when considering a CAC, as responses centred around these themes (Box 3 in Supplement).

Clinical lectureships

Over three-fifths of participants (Table 4), including 65% (n=181) of UK/EU respondents, were at least moderately likely to seek a CL post in England at an appropriate point after completing their doctoral studies. This figure rose to 78% (n=52) for former ACFs/CRFs, and to 98% (n=41) amongst former ACFs/CRFs wanting a CAC. In addition, nearly two-thirds of all participants, and over four-fifths of former ACFs/CRFs (82%, n=55), perceived CL posts to be at least moderately attainable for them (Table 4). Although overall women were more cautious about the attainability of CL posts, amongst respondents wanting a CAC similar proportions of men (74%, n=71) and women (73%, n=46) felt they were at least moderately attainable.

Respondents in speciality training, and those aged 30-35, were the most likely to seek a CL post in England. This is supported by Chi-square tests (Table 5), which also revealed positive associations between likelihood of seeking a CL in England and: wanting a CAC; being from the UK/EU; predoctoral involvement in medical research; knowledge of CLs; and feeling that a CL is attainable. However, there was no statistically significant association between likelihood of seeking a CL post

For peer review only - http://bmjopen.bmj.com/site/about/guidelines.xhtml

and gender (Box 8 in Supplement.) Chi-square tests also showed a positive association between wanting a CAC and perceiving that CL posts are attainable (Table 2).

Table 4 – Respondents' likelihood of seeking a CL, and views on CL attainability and own knowledge about CLs: percentages by gender and for all respondents

	All respondentsǂ		
	Males %	Females %	Total %
**Likelihood of seeking a CL***	*N=173**	*N=141**	*N=318**
Extremely/Very likely	45	40	42
Moderately likely	17	23	20
Slightly/Not at all likely	36	36	36
No response	1	2	2
**CL attainability** *Accuracy of statement that respondent could become a CL in England, if wanted*	*N=174*	*N=142*	*N=320*
Extremely/Very accurate	37	22	30
Moderately accurate	29	36	33
Slightly/Not at all accurate	33	42	37
No response	1	-	<1
**Knowledge of CL** *Amount known*	*N=174*	*N=142*	*N=320*
A great deal/A lot	12	11	12
A moderate amount	40	34	37
A little/Nothing at all	48	54	51
No response	-	1	1

Note: Due to rounding, column percentages may not add to 100.
ǂFour respondents did not provide their gender.
*The question about likelihood of seeking a CL was not asked of two participants who stated that they intended to work in clinical posts without teaching or research.

Although many respondents thought that they may successfully apply for a CL, only a minority (Table 4) felt that they knew a great deal or a lot about CL posts in England. In fact, just over half, including one-quarter (25%, n=17) of former ACFs/CRFs, felt that they had little or no knowledge (Table 4). Also, although Chi-square tests showed a positive association between wanting a CAC and knowledge of CL posts (Table 2), two-fifths (40%, n=63) of those planning a CAC nevertheless had little or knowledge of these CL posts.

For peer review only - http://bmjopen.bmj.com/site/about/guidelines.xhtml

Table 5 – Variables associated with likelihood of seeking a CL: statistically significant X^2 tests

		Respondents extremely/very likely to seek a CL			X^2 test		
**Variables**	**Groupings**	**%**	**n / N**	**Adjusted residuals‡**	**df**	**X^2**	**p**
**Fee status**	UK/EU	47	128 / 273	3.4	1	11.383	.001
	Non-EU (Overseas)	16	5 / 32	-3.4			
**Age**	<30 years	27	12 / 44	-2.5	2	11.534	.003
	30-35 years	51	89 / 173	3.4			
	36 years and over	32	14 / 44	-1.8			
**Training stage**	Pre-specialty	22	8 / 36	-2.7	2	12.149	.002
	In specialty	49	108 / 221	3.4			
	Completed	32	18 / 56	-1.8			
**Predoctoral involvement in medical research**	**No involvement**	21	8 / 38	-2.9	2	24.502	.0005
	**Some involvement** *(intercalated degree or post(s) with research, possibly with other experience, or "other" experience)* §	39	77 / 199	-1.9			
	**High involvement level** *(intercalated degree and post(s) with research, and possibly "other" experience, too)* §	68	42 / 62	4.5			
**Long-term career plans**	CAC	67	105 / 158	8.5	3	72.557	.0005
	Clinical service with research (possibly also teaching)	18	18 / 99	-6.0			
	Undecided	23	8 / 35	-2.5			
	Other plans	15	3 / 20	-2.6			
**CL knowledge**	A great deal/ A lot	65	24 / 37	2.9	2	31.140	.0005
	A moderate amount	57	66 / 116	3.8			
	A little/Nothing at all	28	44 / 158	-5.5			
**CL attainability (accuracy of statement that CL is attainable)**	Extremely/Very accurate	70	66 / 95	6.3	2	52.138	.0005
	Moderately accurate	44	46 / 105	.2			
	Slightly/Not at all accurate	20	22 / 112	-6.2			
Sources of information about possible career paths (amount of information received):							
**Academic/ research supervisor(s)**	A great deal/A lot	48	49 / 103	1.2	2	6.443	.040
	A moderate amount	48	52 / 108	1.3			
	A little/None at all/N/A	33	33 / 101	-2.5			
**Senior academics**	A great deal/A lot	50	31 / 62	1.3	2	9.356	.009
	A moderate amount	53	48 / 91	2.2			
	A little/None at all/N/A	35	55 / 159	-3.0			
**OUCAGS/ACO**	A great deal/A lot	59	16 / 27	1.8	2	17.804	.0005
	A moderate amount	65	33 / 51	3.5			
	A little/None at all/N/A	36	82 / 230	-4.2			
**Mentor(s)**	A great deal/A lot	57	43 / 76	2.7	2	10.053	.007
	A moderate amount	46	37 / 81	.6			
	A little/None at all/N/A	35	53 / 152	-2.9			

‡ For explanation and interpretation see Method.
§The following were considered "posts with research": Academic Foundation Programme posts; Academic Clinical Fellow (England, Wales, Northern Ireland); Clinical Research Fellow (Scotland); and responses of "other post with time for research".

For peer review only - http://bmjopen.bmj.com/site/about/guidelines.xhtml

Career preparedness

Most respondents (64%, n=204) felt that their doctoral programme was preparing them extremely or very well for a possible future CAC, although this figure was higher for men (72%, n=125) than for women (55%, n=78).

A proportion (23%, n=75) of respondents had little or no encouragement from their PhD supervisor(s) to reflect on their career development needs, but most (56%, n=180) had a formal or informal mentor they met regularly. However, only 50% (n=71) of women had such a mentor, compared to 62% (n=107) of men. Also, over a third (34%, n=109) of all respondents stated that they did not have a mentor but would like to find one, and this figure increased to 41% (n=58) amongst women (*vs.* 29%, n=50, amongst men).

Respondents who wanted a CAC were more likely to say that they get a great deal or a lot of information about possible careers from mentors (31%, n=50 *vs.* 18%, n=28 for those who had other plans or were undecided). In contrast, over three-fifths of women *not* intending to pursue a CAC received little or no such information from senior academics (64%, n=50 *vs.* 50%, n=39, of men) and mentors (63%, n=49 *vs.* 45%, n=35, of men). (Box 9 in Supplement contains additional data.)

Men and women obtained similar amounts of information from OUCAGS/UCL's ACO, with 28% (n=48) of men and 22% (n=31) of women receiving at least moderate amounts of information from this source. Moreover, receiving information about careers from OUCAGS/ACO and mentor(s) each had a positive relationship with both wanting a CAC and likelihood of seeking a CL (Tables 2 and 5). The latter also was positively associated with receiving information about careers from academic/research supervisor(s) and senior academics. Interestingly, there were no statistically significant relationships between either wanting a CAC or likelihood of seeking a CL and: having a mentor, encouragement from supervisors to reflect on career needs, or information from peers and newsletters (Boxes 6 and 9 in Supplement).

For peer review only

For peer review only - http://bmjopen.bmj.com/site/about/guidelines.xhtml

DISCUSSION

The clinical PhD students in our study are aged mainly between 30-35 years, in specialty training (with a quarter close to completion), and gender balanced once Overseas students are accounted for. Encouragingly, half of our clinical PhD students want to pursue a CAC, with women as likely as men to express this career goal. Amongst those who have already completed their clinical specialist training (nearly one-fifth of our clinical PhD students) there is also interest in CACs. Indeed, training level does not seem to affect long-term plans, and neither does age. Greater predoctoral involvement in medical research is strongly associated with wanting a CAC but, unexpectedly, less than two-thirds of former ACFs/CRFs, i.e. PhD students already on the clinical academic path, are planning to pursue a CAC. Given that our cohort, who secured doctorates at Oxford/UCL, might be regarded as ‘‘high-achievers’’, it is surprising that a higher proportion did not want a CAC. On a more positive note, many PhD students, even if not planning a CAC, *are* interested in a career that combines clinical work and research, with very few wanting purely clinical posts. In common with others,[9-11] we found that uncertainties around funding and career progression influence career plans, and are perceived as drawbacks of CACs. Respondents not planning a CAC, including former ACFs/CRFs, are particularly worried about the small number of senior academic appointments available, as well as the difficulty of obtaining research grants and work-life balance. Whilst the desire for a career that fits domestic circumstances is not statistically associated with career plans, these issues are given more weight by women than men. Such factors may go on to influence women’s actual postdoctoral career choices,[21] as women may evaluate barriers encountered differently to men, leading to a change in their career intentions.[22]

We were curious about the place of CL posts in career plans. These posts might be considered a logical step in a postdoctoral CAC but a recent report[9] suggests their take-up is lower than expected. In light of this, our data are encouraging as around three-fifths of respondents were at least moderately likely to apply for a CL post – a greater proportion than were planning a CAC. This might indicate that some view CL posts as valuable for career progression, whether as part of a CAC or not. Concomitantly, however, progression to a CL may be affected by the low levels of knowledge about the posts. Additionally, we found some issues with career preparedness (also reported by others[9]). These were more prominent amongst women, who were more likely not to have a mentor but want one and, if not wanting a CAC, received less guidance from senior academics. Exposure to role models, often suggested as important[23,24] in career development, did not appear to be significant amongst our respondents, but the positive association between receiving information from OUCAGS/ACO and wanting a CAC is reassuring as it supports the benefit of such infrastructure.

For peer review only - http://bmjopen.bmj.com/site/about/guidelines.xhtml

Strengths and limitations of the study

Retention of postdoctoral clinical academics is crucial to the progress of academic medicine. However, the relevant literature is predominantly North American[8] and also tends to rely on retrospective data.[9] Ours is the first UK study to describe, based on data from two universities, the career aspirations of medically qualified PhD students and what influences them as they make their career plans. As it is a cross-sectional study, causality cannot be inferred from the associations we found between career plans and other variables. It is also not possible to say whether respondents' intentions will be implemented as planned and our findings may not generalise to other institutions as our data are from two research-intensive universities in England.

Implications of findings

Our study, which adds the perspective of UK clinical PhD students to the existing literature, suggests four areas of action that will support the development of a stable pipeline of clinical academics. Firstly, we suggest promoting increased numbers of flexible opportunities for doctors to gain medical research experience, starting from their undergraduate studies. Indeed, we found that those with the most research experience were more likely to want a CAC and, although research experience may not directly influence that choice, it may contribute to an informed career decision. Secondly, institutions should offer comprehensive career support for their clinical doctoral students. This could be via delivery of a targeted careers package by a local institutional unit similar to OUCAGS/ACO which could: address knowledge about CL posts and other postdoctoral opportunities; facilitate mentors and doctoral students to develop individualised career plans; meet the unfulfilled need for mentors; and brief senior academics on current CAC opportunities to enable them to provide up-to-date guidance. Thirdly, we suggest a need to increase the flexibility of CAC paths even further than at present. CL posts are the first postdoctoral position to afford clinicians the opportunity to develop as independent research leaders, and without this opportunity progressing further academically may be difficult. We have identified that family mobility is an issue for our clinical PhD students, but identifying a CL post in the right specialty, in the right place and at the right time (e.g. as the doctorate comes to an end) is challenging, and sometimes impossible. This is exacerbated by eligibility criteria, particularly for NIHR CL posts, requiring that applicants have at least one year of training remaining (the post ceases at completion of training), since, as we found, many clinical doctoral students have completed, or nearly completed, their training. We therefore suggest that criteria for CL posts be relaxed. Various postdoctoral points of entry into CL posts could be allowed (including after training completion) and the posts should not be specialty-specific so as to afford enhanced opportunities to all postdoctoral clinicians. Increased flexibility might also attract back postdoctoral clinicians who had originally not pursued a CAC, such as when family

For peer review only - http://bmjopen.bmj.com/site/about/guidelines.xhtml

circumstances change and work-life balance may seem more achievable. Such changes could help medical schools overcome the recruitment challenges that have been reported.[7] Finally, more senior posts are required since their lack seems to significantly influence career decisions. Universities are unlikely to be able to create sufficient fully funded senior posts, so formalising research time in a subset of NHS consultant posts, and establishing them as joint university appointments, could cost-effectively expand the definition of a CAC. Ensuring the visibility of these posts would demonstrate a clear career trajectory to encourage postdoctoral retention. Although current NHS service and funding pressures might make this seem low priority, we would argue that such joint appointments are essential as research activity is centrally positioned in the NHS constitution[25] and, crucially, it improves patient care and service quality.[26-27]

Conclusions and further research

Our findings suggest that many doctoral students with the potential to be the clinical academics of the future may never tread this path, some (former ACFs/CRFs) may choose to abandon it, and some may be excluded by inflexible eligibility criteria for postdoctoral posts. It is therefore important for those promoting CACs to provide sufficient, flexible opportunities to engage in clinical academia to all postdoctoral clinicians. This should include tackling issues around the career pathway beyond the clinical training period and building career preparedness and resilience. Our findings also suggest that an additional approach to increasing the numbers of clinical academics might be to ensure predoctoral doctors have ample opportunities to gain research experience. Further research should investigate whether clinical PhD students' intentions will be implemented as planned (e.g. will equal numbers of men and women actually develop CACs?) and seek to identify which factors contribute to actually pursing a CAC. The Clinical DPhil Paths longitudinal study is currently collecting data on this in the UK.

For peer review only

FOOTNOTES

Acknowledgements: We thank the clinical PhD students who took part in our study and Abigail Hipkin (Research Education and Training Administrator, OUCAGS), who assisted with data collection at Oxford. We are also grateful to the University of Oxford and UCL administrative services and the UCL graduate tutors who helped us identify the students to invite to participate in our study.

Contributors: JL, DB and CP developed the Clinical DPhil Paths questionnaire. JL collected the Oxford data and VR collected the UCL data. JL, VR, TL, CP, HB, NJF, GR and DB conceived the data analysis plan and later made substantive contributions to the interpretation of the findings and the writing of this article. JL conducted the quantitative data analysis and reporting, with assistance from VR and expert statistical guidance from TL. JL and VR undertook the qualitative data analysis. JL and DB wrote the first and subsequent drafts. All authors critically reviewed and edited drafts. All authors approved the final version of the manuscript. All authors had full access to all of the data (including statistical reports and tables) in the study and can take responsibility for the integrity of the data and the accuracy of the data analysis. The study guarantors are DB (for Oxford) and GR (for UCL).

Funding: Oxford – This research was funded by OUCAGS, which is part of the University of Oxford Medical School. DB is in part supported by the National Institute for Health Research (NIHR) Oxford Biomedical Research Centre (BRC). UCL – This research was funded by the National Institute for Health Research University College London Hospitals Biomedical Research Centre. NJF and HB were (in part) supported by the National Institute for Health Research (NIHR) Collaboration for Leadership in Applied Health Research and Care (CLAHRC) North Thames at Bart's Health NHS Trust. The views expressed are those of the author(s) and not necessarily those of the NHS, the NIHR or the Department of Health. The authors conducted the study with no involvement from the funders.

Competing interests: All authors have completed the ICMJE uniform disclosure form at http://www.icmje.org/coi_disclosure.pdf and declare: JL, CP and DB declare that JL and DB are employed by, and CP directs, OUCAGS, which exists to promote and advance clinical academic careers; CP declares that his salary is in part paid for by Health Education England in the Thames Valley; VR and GR declare that the former works for, and the latter manages, UCL's Academic Careers Office, which is funded by UCLH's NIHR Biomedical Research Centre, and thus the National Institute for Health Research; no other financial relationships with any organisations that might have an interest in the submitted work in the previous three years besides the support provided for the submitted work by the organisations described above under ''funding''; no other relationships or activities that could appear to have influenced the submitted work.

For peer review only - http://bmjopen.bmj.com/site/about/guidelines.xhtml

Ethical approval: Oxford survey approved by the University of Oxford's Medical Sciences Inter-Divisional Research Ethics Committee (reference MSD-IDREC-C1-2014-149). UCL survey approved by the UCL Research Ethics Committee (application 7335/002). All participants gave informed consent to take part.

Data sharing: No additional data available.

Transparency: The study guarantors affirm that this manuscript is an honest, accurate, and transparent account of the study being reported; that no important aspects of the study have been omitted; and that any discrepancies from the study as planned have been explained.

Licence: The Corresponding Author has the right to grant on behalf of all authors and does grant on behalf of all authors, a worldwide licence (http://www.bmj.com/sites/default/files/BMJ%20Author%20Licence%20March%202013.doc) to the Publishers and its licensees in perpetuity, in all forms, formats and media (whether known now or created in the future), to i) publish, reproduce, distribute, display and store the Contribution, ii) translate the Contribution into other languages, create adaptations, reprints, include within collections and create summaries, extracts and/or, abstracts of the Contribution and convert or allow conversion into any format including without limitation audio, iii) create any other derivative work(s) based in whole or part on the on the Contribution, iv) to exploit all subsidiary rights that currently exist or as may exist in the future in the Contribution, v) the inclusion of electronic links from the Contribution to third party material where-ever it may be located; and, vi) licence any third party to do any or all of the above. All research articles will be made available on an Open Access basis (with authors being asked to pay an open access fee—see http://www.bmj.com/about-bmj/resources-authors/forms-policies-and-checklists/copyright-open-access-and-permission-reuse). The terms of such Open Access shall be governed by a Creative Commons licence—details as to which Creative Commons licence will apply to the research article are set out in our worldwide licence referred to above.

For peer review only - http://bmjopen.bmj.com/site/about/guidelines.xhtml

REFERENCES

1 Academy of Medical Sciences. *The tenure-track clinical scientist: a new career pathway to promote recruitment into clinical academic medicine*. London: AMS, 2000.

2 Academy of Medical Sciences. *Clinical academic medicine in jeopardy: recommendations for change.* London: AMS, 2002 https://www.acmedsci.ac.uk/viewFile/publicationDownloads/pclinaca.pdf (accessed 7 Dec 2016).

3 Milewicz DM, Lorenz RG, Dermody TS, *et al.* Rescuing the physician-scientist workforce: the time for action is now. *J Clin Invest* 2015;125(10):3742-47. doi:10.1172/JCI84170

4 Windsor J, Searle J, Hanney R, *et al.* Building a sustainable clinical academic workforce to meet the future healthcare needs of Australia and New Zealand: report from the first summit meeting. *Intern Med J* 2015;45(9):e965:e971. doi:10.1111/imj.12854

5 Modernising Medical Careers. *Medically- and dentally-qualified academic staff: recommendations for training the researchers and educators of the future. Report of the Academic Careers Sub-Committee of Modernising Medical Careers and the UK Clinical Research Collaboration*. London: UK CRC, 2005 http://www.ukcrc.org/wp-content/uploads/2014/03/Medically_and_Dentally-qualified_Academic_Staff_Report.pdf (accessed 7 Dec 2016).

6 Funston G, Cera C, Kirkham D, *et al.* The road to a clinical academic career. *BMJ Careers 2015*. http://careers.bmj.com/careers/advice/The_road_to_a_clinical_academic_career

7 Medical Schools Council. *A survey of staffing levels of medical clinical academics in UK medical schools, as at 31 July 2015*. London: Medical Schools Council, 2016 http://www.medschools.ac.uk/SiteCollectionDocuments/MSC-survey-2016.pdf (accessed 9 Mar 2017).

8 Edmunds LD, Ovseiko PV, Shepperd S, *et al.* Why do women choose or reject careers in academic medicine? A narrative review of empirical evidence. *Lancet* 2016;Apr19. doi:10.1016/S0140-6736(15)01091-0 (accessed 28 Apr 2016).

9 Medical Research Council, Academy of Medical Sciences, British Heart Foundation, et al. *A cross-funder review of early-career clinical academics: enablers and barriers to progression*. London: MRC, 2015 https://www.mrc.ac.uk/documents/pdf/review-of-early-career-clinical-academics/ (accessed 26 Jan 2017).

10 Ranieri V, Barratt H, Fulop N, *et al.* Factors that influence career progression among postdoctoral clinical academics: a scoping review of the literature. *BMJ Open* 2016;6:e013523. doi:10.1136/bmjopen-2016-013523

For peer review only - http://bmjopen.bmj.com/site/about/guidelines.xhtml

11 Ranieri V, Barratt H, Fulop N, *et al.* Clinical academics' postdoctoral career development. *BMJ* 2015;351:h6945. doi:10.1136/bmj.h6945 (Published 30 December 2015).

12 Lyons OTA, Smith C, Winston JS, *et al.* Impact of UK academic foundation programmes on aspirations to pursue a career in academia. *Med Educ* 2010;44(10):996-1005. doi: 10.1111/j.1365-2923.2010.03787.x

13 Best D, Lopes J, Pugh C. It's the duty of every doctor to get involved with research. *BMJ Online* [eLetter] 27 November 2015. doi:10.1136/bmj.h6329 http://www.bmj.com/content/351/bmj.h6329/rapid-responses (accessed 9 Mar 2017).

14 von Elm E, Altman DG, Egger M, *et al.* The strengthening the reporting of observational studies in epidemiology (STROBE) statement: guidelines for reporting observational studies. *BMJ* 2007;335(7624):806-808. doi: 10.1136/bmj.39335.541782.AD http://www.bmj.com/content/335/7624/806 (accessed 2 May 2017).

15 Hodsdon L, Buckley A. *Postgraduate research experience survey: 2011 results.* Higher Education Academy 2011 www.heacademy.ac.uk/resources/detail/postgraduate/PRES_2011_report (accessed 6 Jul 2016).

16 Goldacre MJ, Lambert T, Goldacre R, *et al.* Career plans and views of trainees in the academic clinical fellowship programme in England. *Med Teach* 2011;33:11:e637-e643. doi: 10.3109/0142159X.2011.610842

17 Ajzen I. The theory of planned behaviour. *Organ Behav Hum Decis Process* 1991;50(2):e179-211.

18 Hsieh H-F, Shannon SE. Three approaches to qualitative content analysis. *Qual Health Res* 2005;18(9):e1277-1288.

19 Bland M. *An introduction to medical statistics*. Oxford: Oxford University Press 2015:193–197.

20 de Leeuw, ED. To mix or not to mix data collection modes in surveys. *J Off Stat* 2005;21(2)e233–255.

21 Levine RB, Lin F, Kern DE, *et al.* Stories from early-career women physicians who have left academic medicine: a qualitative study at a single institution. *Acad Med* 2011;86:6:e752-e758.

22 Ng THW, Sorensen KL, Eby LT, *et al.* Determinants of job mobility: a theoretical integration and extension. *J Occup Organ Psychol* 2007;80: e363-e386. doi:10.1348/096317906X130582

23 Levinson W, Kaufman K, Clark B, *et al.* Mentors and role models for women in academic medicine. *West J Med* 1991;154:423-426.

For peer review only - http://bmjopen.bmj.com/site/about/guidelines.xhtml

24 Borges NJ, Navarro AM, Grover A, *et al.* How, when, and why do physicians choose careers in academic medicine? A literature review. *Acad Med* 2010;85(4):680-6. doi: 10.1097/ACM.0b013e3181d29cb9

25 Department of Health. *NHS constitution for England*. 27 July 2015. www.gov.uk/government/publications/the-nhs-constitution-for-england (accessed 2 Nov 2016).

26 Ozdemir BA, Karthikesalingam A, Sinha S, *et al.* Research activity and the association with mortality. *PLoS ONE* 2015;10(2):e0118253. doi:10.1371/journal.pone.0118253

27 Boaz A, Hanney S, Jones T, *et al.* Does the engagement of clinicians and organisations in research improve healthcare performance: a three-stage review. *BMJ Open* 2015;5:e009415. doi:10.1136/bmjopen-2015-009415

For peer review only

For peer review only - http://bmjopen.bmj.com/site/about/guidelines.xhtml

The clinical academic workforce of the future: a cross-sectional study of factors influencing career decision-making amongst clinical PhD students at two research-intensive UK universities

SUPPLEMENTARY INFORMATION

For peer review only

Box 1 – STROBE diagrams: a) Oxford census survey; b) UCL census survey

Box 2 – Questionnaire development and questions used

Box 3 – Analysis of open questions

Box 4 – Additional information about respondents: a) Gender and fee status of clinical doctoral students: percentages by university and for all respondents; b) Specialty of respondents: percentages by university and for all respondents

Box 5 – Respondents' predoctoral involvement in medical research and reason for the doctorate: percentages by gender and for all respondents

Box 6 – Variables not associated with wanting a clinical academic career (CAC) *vs.* having other plans or being undecided: statistically *non*-significant X^2 tests

Box 7 – Factors influencing respondents' thinking about their long-term career: percentages for those who want a clinical academic career (CAC) and those who do not/are undecided, and for all respondents

Box 8 – Variables not associated with likelihood of seeking a CL: statistically *non*-significant X^2 tests

Box 9 – Sources of information about possible career paths: by gender for those who want a clinical academic career (CAC) and those who have other plans/are undecided, and for all respondents

Box 1 – STROBE diagrams

A. Oxford census survey

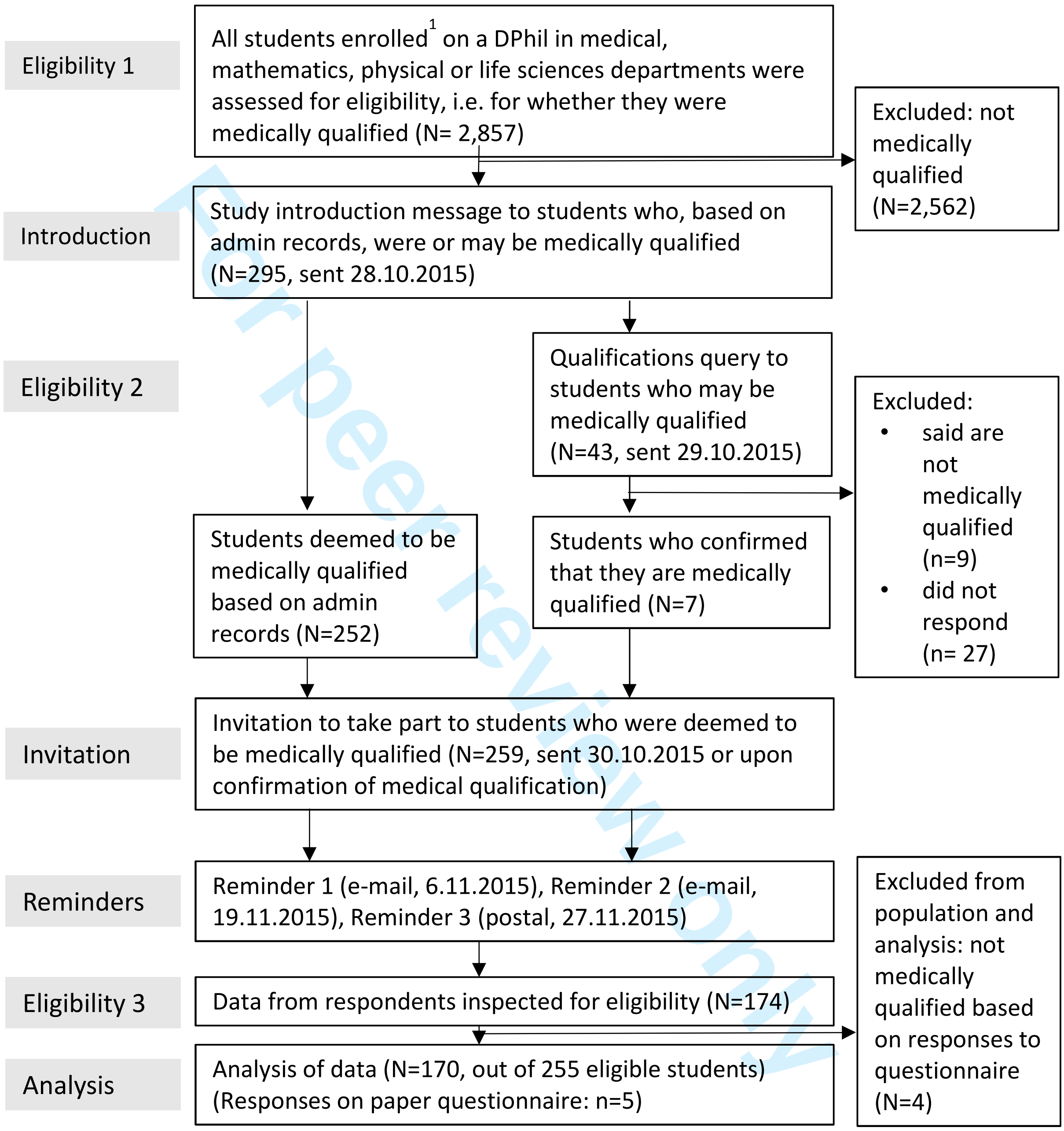

For peer review only

Box 1 (cont.)

B. UCL census survey

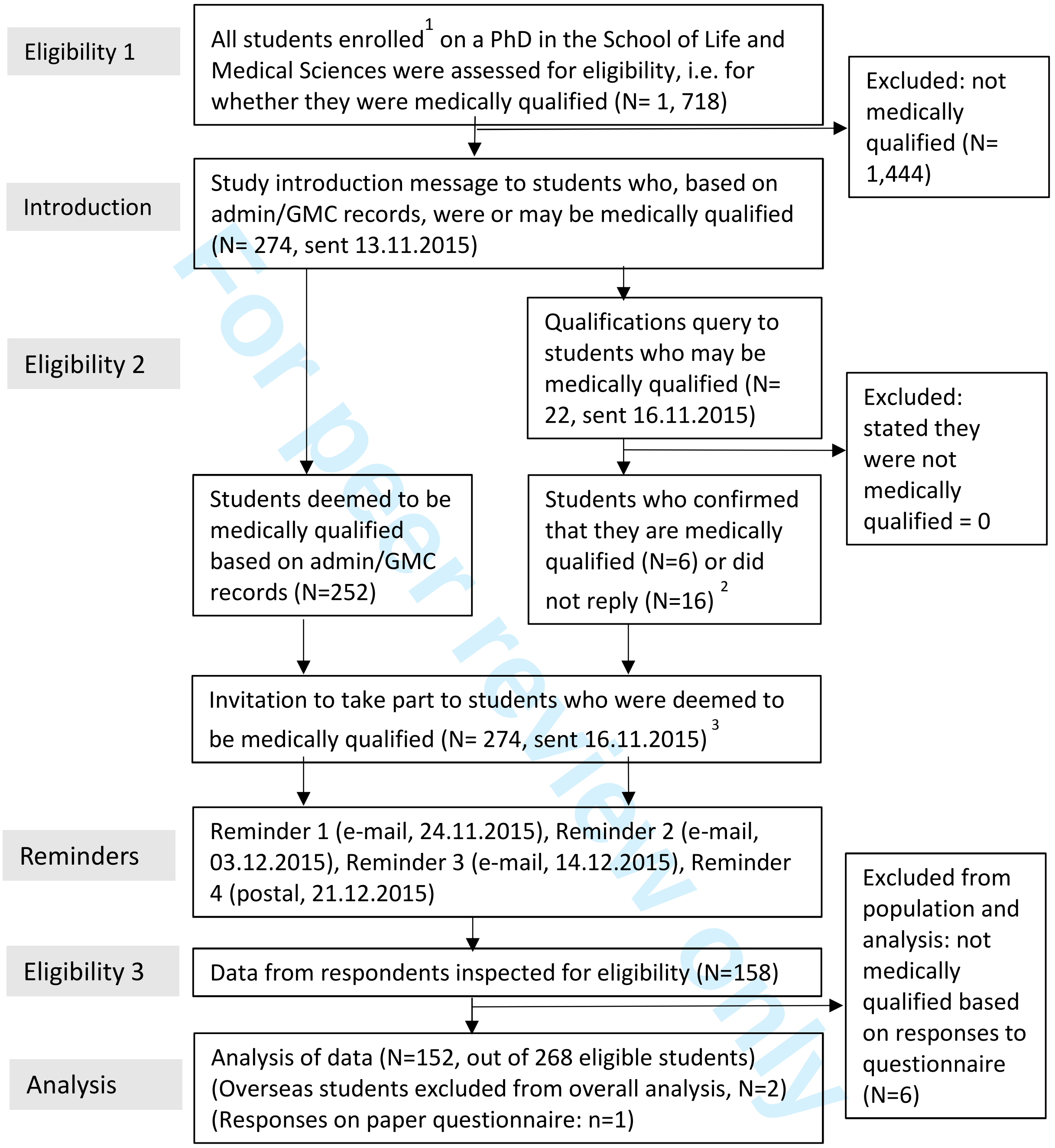

Notes

[1] All DPhil students who were enrolled on a full-time or part-time programme were assessed for eligibility (although at Oxford there were no part-time students). Suspended students were excluded as well as those who had already submitted their thesis.

[2] Both those who confirmed that they were medically qualified and those who did not reply were retained in this census survey.

[3] Due to administrative constraints, a number of students (N=33) were invited on 18.01.16 and data collection finished on 3.03.16.

For peer review only

Box 2 – Questionnaire development and questions used

OUCAGS developed the questionnaire, as part of its Clinical DPhil Paths longitudinal study (2013-2018) of the career plans of clinical doctoral students, as follows:

1) JL developed some of the questions below and adapted others from existing questionnaires.
2) The face validity of the questions was then checked by DB and CP, followed by question refinement by JL, DB and CP.
3) Further face validation was performed in a pilot exercise with three volunteer clinical lecturers who had completed PhDs. (Two volunteers completed the questionnaire online and one on paper.)
4) Questions were revised to address any wording issues which pilot volunteers raised.

Ahead of the 2015 data collection, and following analysis of the Oxford 2014 focus-group data, the question about knowledge of clinical lectureships was added. Also, the wording of the questions about qualifications was reviewed in discussion with UCL and small adjustments were made. For its survey, UCL also made minor adjustments to the wording of questions, such as using the term ''PhD'' instead of ''DPhil''.

0. Qualifications

0.A. What academic qualifications do you hold? (*Tick all that apply)*

1. BSc
2. BMedSci
3. BA
4. MB ChB (or equivalent)
5. MD (taught)
6. MD by research
7. MSc (taught)
8. MSc by research
9. MRes
10. MPhil
11. Other (please specify):

0.B. What fields are your qualifications in? (*Tick all that apply)*

1. Medicine
2. Dentistry
3. Biomedical field
4. Non-medical life, mathematical or physical science (e.g. zoology, chemistry, computer science)
5. Any other field(s) (e.g. humanities, social sciences)

Note: These questions were used to establish whether respondents were medically qualified.

For peer review only

Box 2 (cont.)

1. Medical training

1.A. Which level of training are you currently in? (*Tick one*)

1. Foundation programme (or equivalent) **[→ SKIP Questions 1.B. & 1.C.]**
2. Core training (or equivalent) **[→ SKIP Questions 1.B. & 1.C.]**
3. Acute Care Common Stem (or equivalent) **[→ SKIP Questions 1.B. & 1.C.]**
4. Specialty training (or equivalent) **[→ Go to Question 1.C.]**
5. NOT APPLICABLE: I am between levels of training **[→ Go to Question 1.B.]**
6. NOT APPLICABLE: I have left training and do not plan to return **[→ Go to Question 1.B.]**
7. NOT APPLICABLE: I have fully completed my training **[→ SKIP Questions 1.B. & 1.C.]**

1.B. What is the highest stage of clinical training which you have FULLY COMPLETED so far? (*Tick one*)

1. Medical School **[→ SKIP Question 1.C.]**
2. Foundation Programme (or equivalent) **[→ SKIP Question 1.C.]**
3. Core Training (or equivalent) **[→ SKIP Question 1.C.]**
4. Acute Care Common Stem (or equivalent) **[→ SKIP Question 1.C.]**
5. Specialty Training (or equivalent) **[→ SKIP Question 1.C.]**

1.C. Please specify your Specialty Training level. (*Enter ST or SpR, followed by a number 1 to 10*):
[text box]

1.D. What is, or will be, your medical specialty? *If you have not yet decided, please enter 'not applicable'.*
[text box]

For peer review only

Box 2 (cont.)

2. Prior involvement in research

2.A. What involvement in medical research did you have prior to your [DPhil/PhD]? (*Tick all that apply*)

1. No involvement
2. Intercalated degree
3. Research rotation as an Academic Foundation Programme doctor
4. Research block or regular release as an Academic Clinical Fellow (England, Wales, Northern Ireland)
5. Research block or regular release as a Clinical Research Fellow (Scotland)
6. Other post with time for research
7. Other (please specify)

3. DPhil / PhD

3.A. What was your MAIN reason for choosing to do a [DPhil/PhD]? (*Tick one*)

1. Interest in my area of research
2. Improving my prospects for an academic/research career
3. Improving my prospects for a clinical career outside academia
4. Other reason (please specify)

Note: This question is adapted from Hodsdon and Buckley (2011) – see References.

3.B. Overall, how much encouragement do you receive from your [DPhil/PhD] supervisor(s) to reflect on your career development needs? (*Tick one*)
Response options: A great deal; A lot; A moderate amount; A little; None at all

For peer review only

Box 2 (cont.)

4. Career paths

4.A. In your long-term career, in what type of posts do you mainly intend to work? (*Tick one*)

1. Clinical academic posts **[→ Skip Question 4.B.]**
2. Clinical service posts with some teaching and research
3. Clinical service posts with some research
4. Clinical service posts with some teaching
5. Clinical service posts without teaching or research
6. Research-only posts **[→ Skip Question 4.B.]**
7. Undecided
8. Other (please specify)

Note: This question is adapted from Goldacre *et al.* (2011) – see References.

4.B. How much would each of the following be a reason for excluding a clinical academic career from your long-term career plans? (*Tick one per row*)
Response options: A great deal; A lot; A moderate amount; A little; Not at all; Not applicable to me

1. The difficulty of obtaining research grants
2. The small number of senior academic appointments available
3. The competing pressures from service, teaching, and research
4. The limited future financial prospects compared to alternative careers
5. My perception of less favourable conditions of employment compared with NHS colleagues (in respects other than salary)
6. The possibility of a significantly longer training period to completion of training compared with NHS colleagues
7. Lack of exit paths if pursuing an academic career path and then changing my mind
8. Needing work arrangements that enable me to meet caring responsibilities (for children and/or adults)
9. Family circumstances making me unwilling to move for work
10. Realising that research is not for me
11. Other reasons (please specify)

Note: This question is adapted from Goldacre *et al.* (2011) – see References.

For peer review only - http://bmjopen.bmj.com/site/about/guidelines.xhtml

Box 2 (cont.)

4.C. How much does each of the following factors influence your thinking about your long-term career? (*Tick one per row*)
Response options: A great deal; A lot; A moderate amount; A little; Not at all

1. Wanting a career that fits my domestic circumstances
2. Future financial prospects
3. Wanting a career with acceptable working hours/working conditions
4. Advice from others
5. Wanting intellectual stimulation
6. Wanting to make a difference to patients
7. Lack of posts in other possible career paths
8. Wanting to do what I really want to do (enthusiasm/commitment)
9. Self-appraisal of own skills/aptitudes
10. Career promotion and prospects
11. Exposure to role models
12. Other (please give details)

Note: This question is adapted from Goldacre *et al.* (2011) – see References.

4.D. What might make a clinical academic career more attractive to you?
[text box]

4.E. How well do you feel your [DPhil/PhD] programme will/would prepare you for a clinical academic career, if this is/was your chosen career path? (*Tick one*)
Response options: Extremely well; Very well; Moderately well; Slightly well; Not at all well

4.F. How much information do you get about possible career paths from each of the following? (*Tick one per row*)
Response options: A great deal; A lot; A moderate amount; A little; None at all; Not applicable to me

1. My academic/research supervisor(s)
2. Senior academics in my department
3. My peers undertaking a [DPhil/PhD] at the [University of Oxford / UCL]
4. [OUCAGS / ACO]
5. Mentor(s)
6. Newsletters/publications from medical bodies (e.g. GMC, NIHR)
7. Other (please specify)

For peer review only

Box 2 (cont.)

5. Clinical Lectureships

5.A. Please tell us how accurately the following statement describes you: 'If I wanted, I could become a Clinical Lecturer in England, at an appropriate point after completing my [DPhil/PhD]'. (*Tick one*)
Response options: Extremely accurately; Very accurately; Moderately accurately; Slightly accurately; Not at all accurately

Note 1: respondents who said that, in their long-term career, they intended to work mainly in ''clinical service posts without teaching or research''@ Question 4.A. →**SKIP Question 5.B..**

Note 2: This question is intended to operationalise self-efficacy as described in Ajzen (1991) – see References.

5.B. How likely are you to seek a Clinical Lecturer post in England, at an appropriate point after you complete your [DPhil/PhD]? (*Tick one*)
Response options: Extremely likely; Very likely; Moderately likely; Slightly likely; Not at all likely

Note: This question is intended to operationalise intention (of seeking a CL post) as described in Ajzen (1991) – see References.

5.C. How much do you feel that you know about Clinical Lecturer posts in England (including how to apply and what the posts entail)? (*Tick one*)
Response options: A great deal; A lot; A moderate amount; A little; Nothing at all

6. About respondents

6.A. What is your sex? (*Tick one*)
Response options: Male; Female

Note: To check whether respondents were representative of the whole population, we used administrative data about the whole population of clinical doctoral students invited to respond to our questionnaire. At the University of Oxford, only administrative data about clinical doctoral students' sex (collected as ''male'' or ''female'') was available for use in our study. Data on gender was not available. To ensure that data from our questionnaire would be comparable to the administrative data, we asked participants about their sex rather than their gender. However, in the presentation and discussion of results, we opted to refer to data on whether respondents ticked themselves as being male or female as ''gender'' because this, not their biological sex, is the relevant issue.

6.B. In which year were you born?
[text box: "19_______"]

For peer review only - http://bmjopen.bmj.com/site/about/guidelines.xhtml

Box 2 (cont.)

6.C. Do you have a mentor, or mentors, who you meet regularly? By mentor we mean someone who is not your line manager or supervisor, and who helps you develop your knowledge, work or thinking. (*Tick one*)

1. I have a mentor/mentors, as part of a formalised mentoring relationship
2. I have an informal mentor/mentors
3. I do not have a mentor, but would like to find one
4. I do not have a mentor and do not feel the need, or have the time, for one
5. Other (please specify)

6.D. For fees purposes, what is your normal place of residence registered as? (*Tick one*)
Response options: Home (UK); Other European Union; Non-European Union

6.E. In which calendar year did you start your [DPhil/PhD] programme? (*Tick one*)
Response options: 2011 or earlier; 2012; 2013; 2014; 2015

For peer review only

Box 3 – Analysis of open questions

A. A number of closed questions had a final open item inviting participants to add their own response. However, none of these open items yielded recurrent themes when responses were reviewed by JL (Oxford data) and VR (UCL data).

B. The question "what might make a clinical academic career more attractive to you?" did yield recurrent themes and analysis was as follows:

1) JL produced a coding frame based on the Oxford data.
2) In a face-to-face meeting, JL and VR revised the coding frame with reference to data from both universities, and then used it to double-code anonymised responses from both Oxford and UCL. Any differences in coding between the researchers were discussed and resolved jointly.

Responses centred around two themes:

- **better post availability/a better career pathway** (mentioned by 38%, n=120 of all respondents), for example:

  "Clearer pathways for balancing clinical training and research. As it is, it is very disruptive to either part of the career to pursue a clinical academic pathway." (Oxford participant)

  "Availability of suitable posts, allowing for my (slightly unusual) combination of clinical specialty and research interests." (Oxford participant)

  "Clear pathway for post-doctoral funding e.g buffer period between PhD completion and establishing independence." (UCL participant)

  "If there were more opportunities created by universities to fund part time research integrated with clinical consultant roles; as one nears the end of training and a senior lectureship seems very very unlikely, the choice is either struggle on and ultimately fail or drop research." (UCL participant)

- **greater career/funding security** (mentioned by 28%, n=90 of all participants), for example:

  "Availability of longer term funding and job security" (Oxford participant)

  "More financial and job security - applying for grants every 5 years or losing your job is not appealing. NHS consultants have far more job security once appointed." (UCL participant)

For peer review only

Box 4 – Additional information about respondents

A. Gender and fee status of clinical doctoral students: percentages by university and for all respondents

	Oxford (N=170)		UCL (N=150)		Total (N=320)	
**Gender**	**n**	***%***	**n**	***%***	**n**	***%***
Male	108	***64***	66	***44***	174	***54***
Female	61	***36***	81	***54***	142	***44***
No response	1	***1***	3	***2***	4	***1***
**Fee status**	**n**	***%***	**n**	***%***	**n**	***%***
UK/EU	131	***77***	148	***99***	279	***87***
Overseas	33	***19***	-	***-***	33	***10***
No response	6	***4***	2	***1***	8	***3***

Note: Due to rounding, percentages may not add to 100.

B. Specialty of respondents: percentages by university and for all respondents

	Oxford (N=170)		UCL (N=150)		Total (N=320)	
**Specialties**[§]	**n**	***%***	**n**	***%***	**n**	***%***
Medicine	70	***41***	80	***53***	150	***47***
Surgery	30	***18***	11	***7***	41	***13***
Other	54	***32***	53	***35***	107	***33***
None	16	***9***	6	***4***	22	***7***

Note: Due to rounding, percentages may not add to 100.
[§] Specialties were grouped under "medicine" and "surgery" according to the groupings used *in* GMC. *National training survey 2013: undermining* London: General Medical Council, 2013;13-14 http://www.gmc-uk.org/NTS_2013_autumn_report_undermining.pdf_54275779.pdf (accessed 11 May 2017). In addition, "infectious diseases and microbiology" was classified as "medicine"; any sub-type of "surgery" not listed in the above document (e.g. "transplant surgery" or "paediatric surgery") was classed as "surgery".

For peer review only

Box 5 – Respondents' predoctoral involvement in medical research and reason for the doctorate: percentages by gender and for all respondents

	Males‡ (N=174)		Females‡ (N=142)		Total (N=320)	
	n	%	n	%	n	%
**Previous involvement in medical research‡‡**						
High involvement level *(intercalated degree and post(s) with research*, and possibly "other" experience, too)*	34	20	28	20	62	19
Some involvement *(intercalated degree or post(s) with research*, possibly with other experience, or "other" experience)*	112	64	91	64	206	64
No involvement	22	13	15	11	38	12
No response	6	3	8	6	14	4
**Main reason for doctorate**						
Interest in area of research	85	49	60	42	146	46
Improving prospects for an academic/research career	70	40	61	43	134	42
Improving prospects for a clinical career outside academia	16	9	17	12	33	10
Other	3	2	4	3	7	2

Note: Due to rounding, column percentages may not add to 100.
‡Four respondents did not provide their gender.
‡‡ More than one answer could be given to the question about involvement in medical research prior to the DPhil/PhD – see Box 2, Question 2.A..
*The following response options were considered to be 'posts with research': research rotation as an Academic Foundation Programme doctor; research block or regular release as an Academic Clinical Fellow (England, Wales, Northern Ireland); research block or regular release as a Clinical Research Fellow (Scotland); other post with time for research.

For peer review only

Box 6 – Variables not associated with wanting a clinical academic career (CAC) *vs.* having other plans or being undecided: statistically *non*-significant X^2 tests

		Respondents wanting a CAC		X^2 test		
**Variables**	**Groupings**	**%**	**n / N**	**df**	**X^2**	**p**
**Gender**	Male	55	96 / 174	1	3.430	.064
	Female	45	63 / 141			
**Fee status**	UK/EU	50	139 / 278	1	.027	.869
	Non-EU (Overseas)	52	17 / 33			
**Age**	<30 years	49	22 / 45	2	3.370	.185
	30-35 years	55	96 / 174			
	36 years and over	40	19 / 47			
**Training stage**	Pre-specialty	63	24 / 38	2	3.072	.215
	In specialty	48	108 / 224			
	Completed	47	27 / 57			
**Encouragement from DPhil/PhD supervisor(s) to reflect on career development needs**	A great deal/A lot	53	79 / 150	2	.927	.629
	A moderate amount	47	44 / 94			
	A little/None at all	48	36 / 75			
Influences on long-term career thinking:						
**Wanting a career that fits my domestic circumstances**	A great deal/A lot	51	89 / 175	2	.733	.693
	A moderate amount	51	53 / 105			
	A little/Not at all	43	16 / 37			
**Wanting to make a difference to patients**	A great deal/ A lot	52	140 / 267	2	3.951	.139
	A moderate amount	38	15 / 40			
	A little/ Not at all	36	4 / 11			
**Wanting a career with acceptable working hours/conditions**	A great deal/A lot	46	80 / 174	2	2.494	.287
	A moderate amount	55	60 / 109			
	A little/Not at all	54	19 / 35			
**Future financial prospects**	A great deal/A lot	51	62 / 122	2	.457	.796
	A moderate amount	51	64 / 125			
	A little/Not at all	47	33 / 71			
**Advice from others**	A great deal/A lot	57	39 / 69	2	3.602	.165
	A moderate amount	51	84 / 164			
	A little/Not at all	42	35 / 84			
**Exposure to role models**	A great deal/A lot	55	85 / 156	2	2.668	.263
	A moderate amount	46	48 / 105			
	A little/Not at all	45	25 / 56			

For peer review only - http://bmjopen.bmj.com/site/about/guidelines.xhtml

Box 6 *(continued)*

		Respondents wanting a CAC		X^2 test		
Variables	Groupings	%	n / N	df	X^2	p
Sources of information about possible career paths (amount of information received):						
**Academic/research supervisor(s)**	A great deal/A lot	55	57 / 104	2	2.123	.346
	A moderate amount	51	55 / 109			
	A little/None at all/N/A	45	47 / 105			
**Senior academics in department**	A great deal/A lot	57	36 / 63	2	5.557	.062
	A moderate amount	56	53 / 94			
	A little/None at all/N/A	44	70 / 161			
**Peers at same university**	A great deal/A lot	55	36 / 66	2	1.388	.500
	A moderate amount	52	56 / 108			
	A little/None at all/N/A	47	67 / 144			
**Newsletters /publications**	A great deal/A lot	54	15 / 28	2	2.589	.274
	A moderate amount	59	37 / 63			
	A little/None at all/N/A	48	107 / 225			
Mentoring:						
**Formal/informal mentor, regularly met**	Yes	51	91 / 180	1	.052	.820
	No	49	67 / 136			

For peer review only

Box 7 – Factors influencing respondents' thinking about their long-term career: percentages for those who want a clinical academic career (CAC) and those who do not/are undecided, and for all respondents

	Respondents wanting a CAC‡				Respondents *not* wanting a CAC‡				Total (N=320)	
	Males (N=96)		Females (N=63)		Males (N=78)		Females (N=78)			
	n	%	n	%	n	%	n	%	n	%
**Enthusiasm/commitment**										
A great deal/A lot	94	***98***	62	***98***	71	***91***	70	***90***	301	***94***
(A great deal)*	(67)	***(70)***	(40)	***(64)***	(38)	***(49)***	(43)	***(55)***	(191)	***(60)***
A moderate amount	2	***2***	1	***2***	6	***8***	7	***9***	17	***5***
A little/Not at all	0	***0***	0	***0***	0	***0***	1	***1***	1	***<1***
No response	-	-	-	-	1	***1***	-	-	1	***<1***
**Wanting intellectual stimulation**										
A great deal/A lot	95	***99***	62	***98***	61	***78***	63	***81***	285	***89***
(A great deal)*	(60)	***(63)***	(40)	***(64)***	(28)	***(36)***	(31)	***(40)***	(160)	***(50)***
A moderate amount	1	***1***	1	***2***	15	***19***	14	***18***	32	***10***
A little/Not at all	0	***0***	0	***0***	1	***1***	1	***1***	2	***1***
No response	-	-	-	-	1	***1***	-	-	1	***<1***
**Wanting to make a difference to patients**										
A great deal/A lot	82	***85***	58	***92***	59	***76***	65	***83***	268	***84***
(A great deal)*	(52)	***(54)***	(29)	***(46)***	(30)	***(39)***	(36)	***(46)***	(148)	***(46)***
A moderate amount	11	***12***	4	***6***	14	***18***	10	***13***	40	***13***
A little/Not at all	3	***3***	1	***2***	4	***5***	3	***4***	11	***3***
No response	-	-	-	-	1	***1***	-	-	1	***<1***
**Self-appraisal of own skills/aptitudes**										
A great deal/A lot	63	***66***	44	***70***	37	***47***	46	***59***	192	***60***
A moderate amount	23	***24***	15	***24***	32	***41***	25	***32***	98	***31***
A little/Not at all	10	***10***	4	***6***	8	***10***	7	***9***	29	***9***
No response	-	-	-	-	1	***1***	-	-	1	***<1***
**Wanting a career that fits my domestic circumstances**										
A great deal/A lot	48	***50***	41	***65***	38	***49***	46	***59***	176	***55***
A moderate amount	36	***38***	17	***27***	25	***32***	26	***33***	105	***33***
A little/Not at all	11	***12***	5	***8***	14	***18***	6	***8***	37	***12***
No response	1	***1***	-	-	1	***1***	-	-	2	***1***
**Wanting a career with acceptable working hours/conditions**										
A great deal/A lot	42	***44***	38	***60***	38	***49***	54	***69***	174	***54***
A moderate amount	41	***43***	19	***30***	28	***36***	19	***24***	110	***34***
A little/Not at all	13	***14***	6	***10***	11	***14***	5	***6***	35	***11***
No response	-	-	-	-	1	***1***	-	-	1	***<1***

For peer review only - http://bmjopen.bmj.com/site/about/guidelines.xhtml

Box 7 (continued)

	Respondents wanting a CAC‡				Respondents *not* wanting a CAC‡				Total (N=320)	
	Males (N=96)		Females (N=63)		Males (N=78)		Females (N=78)			
	n	%	n	%	n	%	n	%	n	%
**Exposure to role models**										
A great deal/A lot	52	54	33	52	34	44	35	45	157	49
A moderate amount	29	30	19	30	27	35	28	36	105	33
A little/Not at all	14	15	11	18	16	21	15	19	56	18
No response	1	1	-	-	1	1	-	-	2	1
**Career promotion and prospects**										
A great deal/A lot	53	55	32	51	35	45	25	32	146	46
A moderate amount	28	29	23	37	26	33	38	49	119	37
A little/Not at all	15	16	8	13	16	21	15	19	54	17
No response	-	-	-	-	1	1	-	-	1	<1
**Future financial prospects**										
A great deal/A lot	41	43	21	33	33	42	25	32	122	38
A moderate amount	39	41	25	40	26	33	34	44	126	39
A little/Not at all	16	17	17	27	18	23	19	24	71	22
No response	-	-	-	-	1	1	-	-	1	<1
**Advice from others**										
A great deal/A lot	23	24	16	25	19	24	11	14	69	22
A moderate amount	51	53	33	52	34	44	42	54	164	51
A little/Not at all	21	22	14	22	24	31	25	32	84	26
No response	1	1	-	-	1	1	-	-	3	1
**Lack of posts in other possible career paths**										
A great deal/A lot	7	7	1	2	8	10	13	17	30	9
A moderate amount	9	9	10	16	15	19	19	24	53	17
A little/Not at all	80	83	52	83	54	69	46	59	236	74
(Not at all)*	(47)	(49)	(43)	(68)	(29)	(37)	(26)	(33)	(147)	(46)
No response	-	-	-	-	1	1	-	-	1	<1

Notes: Due to rounding, column percentages may not add to 100. A minority of respondents rated "other" factors as part of the final, open item of the question; as no recurrent themes were identified, this item is not included in the table.
‡Base: All respondents in group who provided their gender and responded to the question about their career plans (N=315).
*Some variables were considerably skewed. Where the mode for the whole group of respondents (total) was either ''a great deal'' or ''not at all'', figures for the modal response-only are provided in brackets.

For peer review only - http://bmjopen.bmj.com/site/about/guidelines.xhtml

Box 8 – Variables not associated with likelihood of seeking a CL: statistically *non*-significant X^2 tests

		Respondents extremely/very likely to seek a CL		X^2 test		
**Variables**	**Groupings**	**%**	**n / N**	**df**	**X^2**	**p**
**Gender**	Male	46	78 / 171	1	.788	.375
	Female	41	56 / 138			
**Encouragement from DPhil/PhD supervisor(s) to reflect on career development needs**	A great deal/A lot	48	70 / 147	2	2.832	.243
	A moderate amount	37	34 / 92			
	A little/None at all	41	30 / 74			
Sources of information about possible career paths (amount of information received):						
**Peers**	A great deal/A lot	44	29 / 66	2	2.626	.269
	A moderate amount	49	51 / 105			
	A little/None at all/N/A	38	54 / 141			
**Newsletters/publications**	A great deal/A lot	50	14 / 28	2	1.312	.519
	A moderate amount	47	29 / 62			
	A little/None at all/N/A	41	90 / 220			
Mentoring:						
**Formal/informal mentor, regularly met**	Yes	44	77 / 177	1	.013	.910
	No	43	57 / 133			

For peer review only

Box 9 – Sources of information about possible career paths: by gender for those who want a clinical academic career (CAC) and those who have other plans/are undecided, and for all respondents

	Respondents wanting a CACǂ				Respondents *not* wanting a CACǂ				Total (N=320)	
	Males (N=96)		Females (N=63)		Males (N=78)		Females (N=78)			
	n	%	n	%	n	%	n	%	n	%
**Academic/research supervisor(s)**										
Great deal/A lot	39	41	18	29	23	30	23	30	105	33
A moderate amount	29	30	26	41	32	41	21	27	109	34
A little/None/N/A	28	29	19	30	23	30	33	42	105	33
No response	-	-	-	-	-	-	1	1	1	<1
**Senior academics in my department**										
Great deal/A lot	27	28	9	14	17	22	9	12	63	20
A moderate amount	29	30	24	38	22	28	18	23	94	29
A little/None/N/A	40	42	30	48	39	50	50	64	162	51
No response	-	-	-	-	-	-	1	1	1	<1
**Peers undertaking a doctorate at Oxford/UCL**										
Great deal/A lot	29	30	7	11	19	24	11	14	66	21
A moderate amount	33	34	23	37	24	31	27	35	108	34
A little/None/N/A	34	35	33	52	35	45	39	50	145	45
No response	-	-	-	-	-	-	1	1	1	<1
**OUCAGS/ACO**										
Great deal/A lot	13	14	9	14	3	4	2	3	27	8
A moderate amount	21	22	12	19	11	14	7	9	52	16
A little/None/N/A	61	64	42	67	64	82	65	83	236	74
(Not applicable) *	(4)	(4)	(6)	(10)	(12)	(15)	(4)	(5)	(26)	(8)
No response	1	1	-	-	-	-	4	5	5	2
**Mentors**										
Great deal/A lot	28	29	22	35	18	23	10	13	78	24
A moderate amount	28	29	12	19	25	32	16	21	81	25
A little/None/N/A	39	41	29	46	35	45	49	63	157	49
(Not applicable) *	(15)	(16)	(15)	(24)	(11)	(14)	(17)	(22)	(60)	(19)
No response	1	1	-	-	-	-	3	4	4	1
**Newsletters/publications from medical bodies**										
Great deal/A lot	11	12	4	6	6	8	6	8	28	9
A moderate amount	23	24	14	22	11	14	14	18	64	20
A little/None/N/A	62	65	45	71	61	78	55	71	225	70
No response	-	-	-	-	-	-	3	4	3	1

Note: Due to rounding, column percentages may not add to 100.
ǂBase: All respondents in group who provided their gender and responded to the question about their career plans (N=315).
**Question attracted "not applicable" responses from over 3% of respondents, so "not applicable"-only responses are provided separately in brackets.*

For peer review only - http://bmjopen.bmj.com/site/about/guidelines.xhtml

STROBE Statement—Checklist of items that should be included in reports of ***cross-sectional studies***

	Item No	Recommendation
**Title and abstract**	1	(*a*) Indicate the study's design with a commonly used term in the title or the abstract [p.1 , in *Title*; p.2, in *Abstract*]
		(*b*) Provide in the abstract an informative and balanced summary of what was done and what was found [p.2, *Abstract*]
**Introduction**		
Background/rationale	2	Explain the scientific background and rationale for the investigation being reported [pp.4-5, *Introduction*]
Objectives	3	State specific objectives, including any prespecified hypotheses [pp.4-5, *Introduction*]
**Methods**		
Study design	4	Present key elements of study design early in the paper [p.6, *Method – Design and procedures*]
Setting	5	Describe the setting, locations, and relevant dates, including periods of recruitment, exposure, follow-up, and data collection [p.6, *Method – Design and procedures*]
Participants	6	(*a*) Give the eligibility criteria, and the sources and methods of selection of participants [p.6, *Method – Design and procedures*]
Variables	7	Clearly define all outcomes, exposures, predictors, potential confounders, and effect modifiers. Give diagnostic criteria, if applicable [p.6, *Method – Instruments*]
Data sources/ measurement	8*	For each variable of interest, give sources of data and details of methods of assessment (measurement). Describe comparability of assessment methods if there is more than one group [p.6, *Method – Instruments*]
Bias	9	Describe any efforts to address potential sources of bias [p.8, *Results – Characteristics of clinical doctoral students: Demographics and training*]
Study size	10	Explain how the study size was arrived at [p.6, *Method – Design and procedures*]
Quantitative variables	11	Explain how quantitative variables were handled in the analyses. If applicable, describe which groupings were chosen and why [pp.6-7 *Method – Analysis*]
Statistical methods	12	(*a*) Describe all statistical methods, including those used to control for confounding [pp.6-7, *Method – Analysis*]
		(*b*) Describe any methods used to examine subgroups and interactions [pp.6-7, *Method – Analysis*]
		(*c*) Explain how missing data were addressed [n/a]
		(*d*) If applicable, describe analytical methods taking account of sampling strategy [n/a]
		(*e*) Describe any sensitivity analyses [n/a]

For peer review only

**Results**		
Participants	13*	(a) Report numbers of individuals at each stage of study—eg numbers potentially eligible, examined for eligibility, confirmed eligible, included in the study, completing follow-up, and analysed [p.6, *Method – Design and procedures* and p.8-9; *Results – Characteristics of clinical doctoral students: Response rates*; Supplement, Box 1]
		(b) Give reasons for non-participation at each stage [p.8, *Results – Characteristics of clinical doctoral students: Response rates;* Supplement, Box1]
		(c) Consider use of a flow diagram [Supplement, Box 1]
Descriptive data	14*	(a) Give characteristics of study participants (eg demographic, clinical, social) and information on exposures and potential confounders [p.8-9, *Results – Characteristics of clinical doctoral students*]
		(b) Indicate number of participants with missing data for each variable of interest [p.8-9, *Results – Characteristics of clinical doctoral students: Demographics and training*; p.10, Table 1; p.15, Table 4]
Outcome data	15*	Report numbers of outcome events or summary measures [*Results*: p.10, Table 1; p.15, Table 4]
Main results	16	(*a*) Give unadjusted estimates and, if applicable, confounder-adjusted estimates and their precision (eg, 95% confidence interval). Make clear which confounders were adjusted for and why they were included [n/a]
		(*b*) Report category boundaries when continuous variables were categorized [n/a]
		(*c*) If relevant, consider translating estimates of relative risk into absolute risk for a meaningful time period [n/a]
Other analyses	17	Report other analyses done—eg analyses of subgroups and interactions, and sensitivity analyses [*Results*: p.12, Table 2; p.16, Table 5]
**Discussion**		
Key results	18	Summarise key results with reference to study objectives [p.18, *Discussion*]
Limitations	19	Discuss limitations of the study, taking into account sources of potential bias or imprecision. Discuss both direction and magnitude of any potential bias [p.19, *Discussion – Strengths and limitations of the study*]
Interpretation	20	Give a cautious overall interpretation of results considering objectives, limitations, multiplicity of analyses, results from similar studies, and other relevant evidence [pp.18-20, *Discussion*]
Generalisability	21	Discuss the generalisability (external validity) of the study results [p.19, *Discussion –Strengths and limitations of the study*]
**Other information**		
Funding	22	Give the source of funding and the role of the funders for the present study and, if applicable, for the original study on which the present article is based [p.21]

For peer review only - http://bmjopen.bmj.com/site/about/guidelines.xhtml

*Give information separately for exposed and unexposed groups.

Note: An Explanation and Elaboration article discusses each checklist item and gives methodological background and published examples of transparent reporting. The STROBE checklist is best used in conjunction with this article (freely available on the Web sites of PLoS Medicine at http://www.plosmedicine.org/, Annals of Internal Medicine at http://www.annals.org/, and Epidemiology at http://www.epidem.com/). Information on the STROBE Initiative is available at www.strobe-statement.org.

For peer review only

For peer review only - http://bmjopen.bmj.com/site/about/guidelines.xhtml
